# Uniform convergence to equilibrium for a family of drift–diffusion models with trap-assisted recombination and the limiting Shockley–Read–Hall model

**DOI:** 10.1007/s41808-020-00068-8

**Published:** 2020-05-07

**Authors:** Klemens Fellner, Michael Kniely

**Affiliations:** 1 Institute of Mathematics and Scientific Computing, University of Graz, Heinrichstraße 36, 8010 Graz, Austria; 2 Institute of Science and Technology Austria (IST Austria), Am Campus 1, 3400 Klosterneuburg, Austria

**Keywords:** Drift–diffusion–recombination models, Semiconductors, Shockley–Read–Hall, Trapped states, Entropy method, Convergence to equilibrium, Exponential rate of convergence, Quasi-steady-state approximation, Fast-reaction limit, Primary 35K57, Secondary 35B40, 35B45, 82D37

## Abstract

In this paper, we establish convergence to equilibrium for a drift–diffusion–recombination system modelling the charge transport within certain semiconductor devices. More precisely, we consider a two-level system for electrons and holes which is augmented by an intermediate energy level for electrons in so-called trapped states. The recombination dynamics use the mass action principle by taking into account this additional trap level. The main part of the paper is concerned with the derivation of an entropy–entropy production inequality, which entails exponential convergence to the equilibrium via the so-called entropy method. The novelty of our approach lies in the fact that the entropy method is applied uniformly in a fast-reaction parameter which governs the lifetime of electrons on the trap level. Thus, the resulting decay estimate for the densities of electrons and holes extends to the corresponding quasi-steady-state approximation.

## Introduction and main results

The formulation and mathematical analysis of drift–diffusion type semiconductor models reach back to the middle of the last century, see e.g. [18, 20, 24] and the references therein; yet these models still form a highly relevant workhorse in the simulation of semiconductor devices and batteries.

Physically, drift–diffusion models describe the transport of charge carriers via diffusion and convection governed by electric fields. In semiconductors, charge carriers are electrons and holes (positively charged quasi-particles, which represent the absence of an electron). Pairs of electrons and holes can be “generated” and “destroyed” by recombination processes. Generation of an electron-hole pair occurs when an electron is lifted from a low-energy valence band to a high-energy conduction band, where electrons are mobile—leaving behind an equally mobile hole in the valence band. A pivotal generation–recombination model was formulated by Shockley, Read and Hall [16, 21]. Mathematically, Shockley–Read–Hall recombination introduces quadratic non-linear reaction terms into the drift–diffusion dynamics.

**Fig. 1:**
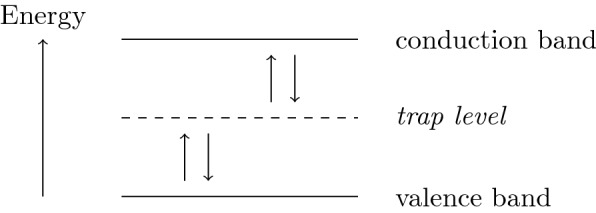
A schematic picture illustrating the allowed transitions of electrons between the various energy levels

A derivation of the Shockley–Read–Hall model considers a generation–recombination process as sketched in Fig. 1. It assumes that appropriately distributed foreign atoms in the crystal lattice of the semiconductor material facilitate the generation of electron-hole pairs by providing in-between energy levels, requiring smaller amounts of energy for each step. Since electrons are immobile at these in-between energy levels, they are called *trapped states*. Also their maximal density is limited. The Shockley–Read–Hall model of electron-hole recombination is obtained as a quasi-steady-state approximation of the trapped-state dynamics as detailed in the following.

We denote the charge densities of electrons, holes and trapped states by *n*, *p* and $n_{tr}$ and consider the following PDE–ODE drift–diffusion–recombination system:

1 $$\begin{aligned} {\left\{ \begin{array}{ll} \begin{aligned} \partial _t n &{}= \nabla \cdot J_n(n) + R_n(n,n_{tr}), \\ \partial _t p &{}= \nabla \cdot J_p(p) + R_p(p,n_{tr}), \\ \varepsilon \, \partial _t n_{tr} &{}= R_p(p, n_{tr}) - R_n(n, n_{tr}), \end{aligned} \end{array}\right. } \end{aligned}$$

with the drift–diffusion fluxes and reaction terms

$$\begin{aligned} J_n &:= \nabla n + n\nabla V_n = \mu _n \nabla \left( \frac{n}{\mu _n}\right) , & \mu _n &:= e^{-V_n}, \\ J_p &:= \nabla p + p\nabla V_p = \mu _p \nabla \left( \frac{p}{\mu _p}\right) , & \mu _p &:= e^{-V_p}, \\ R_n &:= \frac{1}{\tau _n} \left( n_{tr} - \frac{n}{n_0 \mu _n} (1 - n_{tr}) \right) , \\ R_p &:= \frac{1}{\tau _p} \left( 1 - n_{tr} - \frac{p}{p_0 \mu _p} n_{tr} \right) . \end{aligned}$$

The constants $n_0, p_0, \tau _n, \tau _p>0$ are positive recombination parameters and $\varepsilon \in (0,\varepsilon _0]$ for arbitrary $\varepsilon _0>0$ is a positive relaxation parameter. $V_n$ and $V_p$ represent external time-independent potentials.

The reaction term $R_n$ models transitions of electrons from the trap level to the conduction band (proportional to $n_{tr}$) and vice versa (proportional to $-n(1-n_{tr})$), where the maximum capacity of the trap level is normalised to one. The analogue processes with respect to the valence band are described by $R_p$. Note that the rate of hole generation is equivalent to the rate of electrons moving from the valence band to the trap level, which is proportional to ($1-n_{tr}$). Similarly, the annihilation of a hole corresponds to an electron that jumps from the trap level to the valence band, which yields a reaction rate proportional to $-pn_{tr}$. Moreover, $n_0, p_0 > 0$ represent reference levels for the charge concentrations *n* and *p*, while $\tau _n, \tau _p > 0$ are inverse reaction parameter. Note that the concentration of trapped states satisfies $n_{tr} \in [0,1]$ provided this holds true for their initial concentration (cf. Theorem 1.1).

The dynamical equation for $n_{tr}$ in (1) is an ODE in time and pointwise in space with a right hand side depending on *n*, *p* and $n_{tr}$ via $R_n$ and $R_p$. We stress that there is no drift–diffusion term for $n_{tr}$ since trapped electrons are immobile. This is due to the correlation between foreign atoms and the corresponding trap levels which are locally bound near these crystal impurities.

The parameter $\varepsilon > 0$ models the lifetime of trapped states, where lifetime refers to the expected time until an electron in a trapped state moves either to the valence or the conduction band. The Shockley–Read–Hall recombination model is obtained in the (formal) limit $\varepsilon \rightarrow 0$, where the concentration of trapped states is determined from the algebraic relation $0 = R_p(p, n_{tr}) - R_n(n, n_{tr})$:

$$\begin{aligned} n_{tr}^{qssa} = \frac{\tau _n + \tau _p \frac{n}{n_0 \mu _n}}{\tau _n + \tau _p + \tau _n \frac{p}{p_0 \mu _p} + \tau _p \frac{n}{n_0 \mu _n}}. \end{aligned}$$

In this quasi-steady-state approximation, the density of trapped states $n_{tr}$ and its evolution are eliminated from system (1), while the evolutions of the charge carriers *n* and *p* are subject to the Shockley–Read–Hall recombination terms

$$\begin{aligned} R_n\left( n, n_{tr}^{qssa}\right) = R_p\left( p, n_{tr}^{qssa}\right) = \frac{1 - \frac{n p}{n_0 p_0 \mu _n \mu _p}}{\tau _n \left( 1 + \frac{p}{p_0 \mu _p}\right) + \tau _p \left( 1 + \frac{n}{n_0 \mu _n}\right) }. \end{aligned}$$

A rigorous proof of this quasi-steady-state approximation has been performed in [15], even for more general models. See also [18] for semiconductor models with reaction terms of Shockley–Read–Hall-type.

We complete the mathematical description by considering system (1) on a bounded domain $\Omega \subset {\mathbb {R}}^m$, $m \ge 1$, with sufficiently smooth boundary $\partial \Omega$. Without loss of generality, we suppose that the volume of $\Omega$ is normalised, i.e. $|\Omega |=1$, which can be achieved by an appropriate scaling of the spatial variables.

We impose no-flux boundary conditions for $J_n$ and $J_p$,

2 $$\begin{aligned} {\hat{n}} \cdot J_n = {\hat{n}} \cdot J_p = 0 \quad \text{ on } \ \partial \Omega , \end{aligned}$$

where ${\hat{n}}$ denotes the outer unit normal vector on $\partial \Omega$, and we prescribe non-negative and bounded initial data $n_I$, $p_I$, $n_{tr,I} \in L^\infty (\Omega )$ together with $\Vert n_{tr,I} \Vert _{L^\infty (\Omega )} \le 1$. As a consequence, the following charge conservation law holds:

3 $$\begin{aligned} \int _{\Omega } (n-p + \varepsilon \, n_{tr}) \, dx = \int _{\Omega } (n_I - p_I + \varepsilon \, n_{tr,I}) \, dx =: M \end{aligned}$$

with $M \in {\mathbb {R}}$. Finally, the potentials $V_n$ and $V_p$ are assumed to satisfy

4 $$\begin{aligned} V_n, V_p \in W^{2,\infty }(\Omega ) \quad \text{ and } \quad {\hat{n}} \cdot \nabla V_n, \ {\hat{n}} \cdot \nabla V_p \ge 0 \quad \text{ on } \ \partial \Omega , \end{aligned}$$

where the last condition means that the potentials are confining.

The main goal of this paper is to prove exponential convergence to equilibrium of system (1)–(4) with explicit bounds on rates and constants, which are *independent* of the relaxation time $\varepsilon$. We therefore consider $\varepsilon \in (0, \varepsilon _0]$ for arbitrary but fixed $\varepsilon _0 > 0$. Our study also includes the limiting case $\varepsilon =0$.

The main tool in quantifying the large-time behaviour of global solutions to system (1) is the entropy functional

5 $$\begin{aligned} E(n,p,n_{tr})= & {} \int _{\Omega } \left( n \ln \frac{n}{n_0 \mu _n} - (n-n_0\mu _n) + p \ln \frac{p}{p_0 \mu _p} - (p-p_0\mu _p) \right. \nonumber \\&\quad \left. + \varepsilon \int _{1/2}^{n_{tr}} \ln \left( \frac{s}{1-s} \right) ds \right) dx. \end{aligned}$$

For *n* and *p*, we encounter contributions of the Boltzmann-entropy form $a \ln a - (a - 1) \ge 0$, whereas $n_{tr}$ enters the entropy functional via a non-negative integral term. Note that the integral $\int _{1/2}^{n_{tr}} \ln \bigl ( \frac{s}{1-s} \bigr ) ds$ is non-negative and well-defined for all $n_{tr}(x)\in [0,1]$. By introducing the entropy production functional

6 $$\begin{aligned} P := -\frac{d}{dt} E, \end{aligned}$$

it holds (formally) true along solution trajectories of system (1), (2) that

7 $$\begin{aligned} P(n,p,n_{tr})&= \int _{\Omega } \left( \frac{|J_n|^2}{n} + \frac{|J_p|^2}{p} + R_n \ln \left( \frac{n_{tr} n_0 \mu _n }{n(1-n_{tr})} \right) \right. \nonumber \\&\quad \left. + R_p \ln \left( \frac{(1-n_{tr})p_0 \mu _p }{p n_{tr}} \right) \right) dx\ge 0. \end{aligned}$$

The entropy production functional consists of two non-negative flux terms and two equally non-negative reaction terms of the form $(a-1) \ln a \ge 0$. Thus, the entropy *E* and its production *P* are non-negative functionals, which formally implies the entropy *E* to be monotonically decreasing in time.

In a rigorous proof of the entropy decay, one has to control the two reaction terms in (7), which are unbounded for $n_{tr}(t,x)\rightarrow 0,1$ or $n(t,x),p(t,x)\rightarrow 0$. Hence the entropy production is potentially unbounded even for smooth solutions.

The following Theorem 1.1 comprises sufficient existence and regularity results for solutions to satisfy the weak version of (6). We shall call a global weak solution to system (1)–(4) a triple $(n, p, n_{tr}) : [0, \infty ) \rightarrow H^1(\Omega )^2 \times L^\infty (\Omega )$ such that, first,

8 $$\begin{aligned} n, p \in W_2(0, T) {:}{=}\left\{ f \in L^2((0, T), H^1(\Omega )) \, | \, \partial _t f \in L^2((0, T), H^1(\Omega )^*) \right\} \end{aligned}$$

for all $T \in (0, \infty )$ and, second, $(n,p,n_{tr})$ solves (1) where *n* and *p* satisfy their dynamic equations and the boundary conditions (2) in the weak sense. From PDE-theory (see e.g. [3]), we further obtain the embedding $W_2(0, T) \hookrightarrow C([0, T], L^2(\Omega ))$.

### Theorem 1.1

(Time-dependent system) Let $n_0, p_0, \tau _n, \tau _p$ and $\varepsilon$ be positive constants. Assume that $V_n$ and $V_p$ satisfy (4) and that $\Omega \subset {\mathbb {R}}^m$, $m \ge 1$, is a bounded, sufficiently smooth domain.

Then, for any non-negative initial datum $(n_I, p_I, n_{tr,I}) \in L^\infty (\Omega )^3$ satisfying $\Vert n_{tr,I}\Vert _{L^\infty (\Omega )} \le 1$, there exists a unique non-negative global weak solution $(n, p, n_{tr})$ of system (1) with boundary conditions (2). More precisely, we find that for all $T \in (0, \infty )$

9 $$\begin{aligned} n, p \in W_2(0,T) \cap L^\infty ((0, T), L^\infty (\Omega )), \end{aligned}$$

and

10 $$\begin{aligned} n_{tr} \in C([0, T], L^\infty (\Omega )), \quad \partial _t n_{tr} \in C([0, T], L^2(\Omega )). \end{aligned}$$

Moreover, there exist positive constants $C_n(\Vert n_I\Vert _{L^\infty (\Omega )},V_n)$, $C_p(\Vert p_I\Vert _{L^\infty (\Omega )},V_p)$ and $K_n(V_n)$, $K_p(V_p)$ independent of $\varepsilon$ such that

11 $$\begin{aligned} \Vert n(t,\cdot )\Vert _{L^\infty (\Omega )} \le C_n + K_nt,\quad \Vert p(t,\cdot )\Vert _{L^\infty (\Omega )} \le C_p + K_pt,\quad \text {for all } t\ge 0. \end{aligned}$$

In addition, the concentration $n_{tr}(t, x)$ is bounded away from zero and one in the sense that for all times $\tau >0$ there exist positive constants $\eta = \eta (\varepsilon _0, \tau , \tau _n, \tau _p)$, $\theta = \theta (C_n,C_p,K_n,K_p)$ and a sufficiently small constant $\gamma (\tau ,C_n,C_p,K_n,K_p)>0$ such that

12 $$\begin{aligned} n_{tr}(t,x) \in \left[ \min \left\{ \eta t, \frac{\gamma }{1+\theta t}\right\} , \max \left\{ 1-\eta t,1-\frac{\gamma }{1+\theta t}\right\} \right] \, \text {for all } t\ge 0 \text { and a.e. } x\in \Omega , \end{aligned}$$

where $\eta \tau = \frac{\gamma }{1+\theta \tau }$ such that the linear and the inverse linear bound intersect at time $\tau$. As a consequence of (12), there exist positive constants $\mu$, $\Gamma >0$ (depending on $\tau$, $\eta$, $\theta$, $\gamma$, $V_n$, $V_p$) such that

13 $$\begin{aligned} n(t,x),p(t,x) \ge \min \left\{ \mu \frac{ t^2}{2}, \frac{\Gamma }{1+\theta t}\right\} \, \, \text {for all } t\ge 0 \text { and a.e. } x\in \Omega \end{aligned}$$

where $\mu \frac{\tau ^2}{2} = \frac{\Gamma }{1+\theta \tau }$ such that the quadratic and the inverse linear bound intersect at the same time $\tau$.

### Remark 1.2

(Proof of Theorem 1.1) The existence theory of Theorem 1.1 for the coupled ODE-PDE problem (1) applies standard parabolic methods and pointwise ODE estimates. It relates to previous results like [15] in assuming $L^\infty$ initial data and proving $L^\infty$-bounds in order to control non-linear terms. The proof is therefore postponed to the Appendix.

Our first main result proves exponential convergence of solutions to (1)–(4) to a unique positive equilibrium state $(n_\infty (x), p_\infty (x), n_{tr,\infty })$, which is stated in detail in Theorem 2.1.

### Theorem 1.3

(Exponential convergence to equilibrium) Let $(n,p,n_{tr})$ be a global weak solution of system (1)–(4) as given in Theorem 1.1 above corresponding to the non-negative initial data $(n_I,p_I,n_{tr,I}) \in L^\infty (\Omega )^3$ satisfying $\Vert n_{tr,I}\Vert _{L^{\infty }(\Omega )} \le 1$. Then, this solution satisfies the weak entropy production law

14 $$\begin{aligned} E(n,p,n_{tr})(t_1) + \int _{t_0}^{t_1} P(n, p, n_{tr})(s) \, ds = E(n,p,n_{tr})(t_0) \end{aligned}$$

for all $0< t_0 \le t_1 < \infty$ and the following versions of the exponential decay towards the equilibrium:

$$\begin{aligned} E(n,p,n_{tr})(t) - E_\infty \le (E_I - E_\infty ) e^{-K t}, \end{aligned}$$

where $E_I$ and $E_\infty$ denote the initial entropy and the equilibrium entropy of the system, respectively, and the equilibrium $(n_\infty , p_\infty , n_{tr,\infty })$ is given in Theorem 2.1. Moreover,

15 $$\begin{aligned} \Vert n - n_\infty \Vert _{L^1(\Omega )}^2 + \Vert p - p_\infty \Vert _{L^1(\Omega )}^2 + \varepsilon \Vert n_{tr} - n_{tr,\infty } \Vert _{L^2(\Omega )}^2 \le C (E_I - E_\infty ) e^{-K t} \end{aligned}$$

where $C := C_{\mathrm {CKP}}^{-1}$ and $K := C_{\mathrm {EEP}}^{-1}$ (see Theorem 1.5 and Proposition 6.1 for the definition of $C_\mathrm {EEP}$ and $C_\mathrm {CKP}$, respectively) are explicitly computable constants independent of $\varepsilon \in (0, \varepsilon _0]$ for arbitrary but fixed $\varepsilon _0 > 0$.

### Remark 1.4

(Theorem 1.1 proves the weak entropy production law (14)) The regularity of *n* and *p* of Theorem 1.1 as well as the lower and upper bounds (12) for $n_{tr}$ and the lower bounds (13) for *n* and *p* allow to prove that any solution of Theorem 1.1 satisfies the weak entropy production law (14).

The proof of Theorem 1.3 applies the so-called entropy method, which derives a functional inequality of the form

$$\begin{aligned} E(n,p,n_{tr}) - E(n_\infty , p_\infty , n_{tr,\infty }) \le C\,P(n,p,n_{tr}), \end{aligned}$$

where *n*, *p* and $n_{tr}$ are non-negative functions satisfying the same conservation law as solutions to (1)–(4), see below. The proof provides an explicit estimate of the constant $C>0$. Applying this entropy–entropy production (EEP) inequality to the entropy production law (14) entails exponential decay of the relative entropy via a general Gronwall-argument. A Csiszár–Kullback–Pinsker-type inequality yields then exponential convergence in $L^1$ as stated in (15).

The key step of the entropy method is to prove (as second main result) a suitable EEP functional inequality independently from solutions to (1)–(4) and independently from $\varepsilon$.

### Theorem 1.5

(Entropy–Entropy Production Inequality) Let $\varepsilon _0$, $\tau _n$, $\tau _p$, $n_0$, $p_0$ be positive constants and $M\in {\mathbb {R}}$. Let $(n_\infty , p_\infty , n_{tr,\infty })$ be the corresponding equilibrium as in Theorem 2.1. Consider an arbitrarily large positive constant $M_1>0$ and non-negative functions $(n,p,n_{tr}) \in L^1(\Omega )^3$ satisfying the $L^1$-bound $\overline{n}, \overline{p} \le M_1$, the $L^\infty$-bound $\Vert n_{tr}\Vert _{L^{\infty }(\Omega )} \le 1$, and the conservation law

$$\begin{aligned} \overline{n} - \overline{p} + \varepsilon \overline{n_{tr}} = M \end{aligned}$$

where $$\overline{f} := \int _{\Omega } f(x) \, dx$$ (recall $$|\Omega |=1$$).

Then, there exists an explicitly computable constant $C_{\mathrm {EEP}} > 0$ such that for all $\varepsilon \in (0, \varepsilon _0]$ the following functional inequality, called entropy–entropy production inequality, holds true:

16 $$\begin{aligned} E(n,p,n_{tr}) - E(n_\infty , p_\infty , n_{tr,\infty }) \le C_{\mathrm {EEP}} P(n,p,n_{tr}). \end{aligned}$$

### Remark 1.6

We point out that Theorem 1.5 derives a general functional inequality for admissible functions $(n,p,n_{tr})$, which only share few natural properties like the $L^1$-integrability, boundedness of the trapped states and the conservation law with solutions to (1)–(4). It is a nice robustness feature of the entropy method to be based on functional inequalities which can be reused in related contexts, rather than deriving solution-specific estimates. The constant $C_{\mathrm {EEP}}$ is independent of $\varepsilon \in (0, \varepsilon _0]$. It only depends on the upper bound $\varepsilon _0 > 0$, which can be chosen arbitrarily.

### Remark 1.7

We emphasise that the EEP inequality (16) does not depend on the lower and upper solution bounds (11)–(13). These bounds are only needed to prove that solutions to (1)–(4) satisfy the weak entropy production law (14), which is neither directly obvious nor part of the existence theory. Therefore, (14) implies that solutions to Theorem 1.1 may only feature singularities of *P* at time zero due to a lacking regularity of the initial data or due to initial data $n_{tr,I}(x)\in [0,1]$, $n_I(x),p_I(x)\in [0,\infty ).$

The proof of the EEP-inequality of Theorem 1.5 captures in a certain sense the entire non-linear and global dynamics of system (1)–(4). Hence, its derivation is ought to be an involved task. A key step is the proof of a functional EEP-inequality for the special cases of spatially homogeneous concentrations, which fulfil the conservation law (3) and the $L^1$-bounds (cf. Proposition 5.3). This core estimate is then extended to the case of arbitrary concentrations satisfying the same assumptions in Proposition 5.5. This extension also forces one to bound $\sqrt{n} - \overline{\sqrt{n}}$, $\sqrt{p} - \overline{\sqrt{p}}$, and $\sqrt{n_{tr}} - \overline{\sqrt{n_{tr}}}$ in $L^2(\Omega )$ by the entropy production. Due to the diffusive part in the dynamical equations for *n* and *p*, this is easily achieved for the expressions involving *n* and *p* by applying Poincaré’s inequality (see the Proof of Theorem 1.5 in Sect. 6). However, this is not possible for $n_{tr}$ as no diffusion is acting on $n_{tr}$. On the other hand, $n_{tr}$ is subject to *indirect diffusive effects*, which allow for a control on $\sqrt{n_{tr}} - \overline{\sqrt{n_{tr}}}$ in terms of a suitable functional inequality. Indirect diffusive effects occur when a reversible reaction transfers diffusive behaviour from a diffusive species to a non-diffusive species. A first functional inequality which quantifies an indirect diffusion effect was proven in [4] with significant generalisations to reaction–diffusion systems in [8, 12], volume–surface reaction–diffusion systems [11] and reaction–diffusion systems with non-linear diffusion [13]. Here, the corresponding estimate is proven in Proposition 5.6 and might also be of independent interest.

Our two last results on system (1)–(4) combine the exponential convergence to equilibrium as proven in Theorem 1.3 with the solution bounds of Theorem 1.1. This entails uniform-in-time solution bounds for *n* and *p* as well as exponential convergence to equilibrium in $L^\infty (\Omega )$ for *n*, *p*, and $n_{tr}$. As opposed to (15), the convergence result for $n_{tr}$ in Corollary 1.9 holds true without the coefficient $\varepsilon$.

### Corollary 1.8

The solutions *n* and *p* of Theorem 1.1 are uniformly-in-time bounded in $L^{\infty }$, i.e. there exists a constant $Z > 0$ independent of $\varepsilon \in (0, \varepsilon _0]$ such that

17 $$\begin{aligned} \Vert n(t,\cdot )\Vert _{L^\infty (\Omega )} , \Vert p(t,\cdot )\Vert _{L^\infty (\Omega )}\le Z \quad \text {for all}\quad t\ge 0. \end{aligned}$$

Moreover, the bounds (17) allow to improve the bounds (12), (13) and to obtain uniform-in-time bounds in the sense that for all $\tau >0$, there exist sufficiently small and $\varepsilon$-independent constants $\eta , \gamma , \mu , \Gamma > 0$ such that

18 $$\begin{aligned} n_{tr}(t,x) \in \left[ \min \bigl \{\eta t, \gamma \bigr \}, \max \bigl \{1-\eta t,1-\gamma \bigr \}\right] \end{aligned}$$

and

19 $$\begin{aligned} n(t,x),p(t,x) \ge \min \Bigl \{\mu \frac{ t^2}{2}, \Gamma \Bigr \} \end{aligned}$$

for all $t\ge 0$ and a.e. $x\in \Omega$ where $\eta t$ and $\gamma$ as well as $\mu t^2/2$ and $\Gamma$ intersect at time $\tau > 0$.

### Corollary 1.9

Under the hypotheses of Theorem 1.3, there exist constants $0< C, K < \infty$ independent of $\varepsilon \in (0, \varepsilon _0]$ such that

$$\begin{aligned} \Vert n - n_\infty \Vert _{L^\infty (\Omega )} + \Vert p - p_\infty \Vert _{L^\infty (\Omega )} + \Vert n_{tr} - n_{tr,\infty } \Vert _{L^\infty (\Omega )} \le C e^{-Kt} \end{aligned}$$

is valid for all $t \ge 0$.

The final topic of this paper considers the limit $\varepsilon \rightarrow 0$, which recovers the well-known Shockley–Read–Hall drift–diffusion–recombination model (see [15, 18]):

20 $$\begin{aligned} {\left\{ \begin{array}{ll} \begin{aligned} \partial _t n &{}= \nabla \cdot J_n(n) + R(n,p), & J_n &{}= \nabla n + n\nabla V_n, \\ \partial _t p &{}= \nabla \cdot J_p(p) + R(n,p), & J_p &{}= \nabla p + p\nabla V_p, \end{aligned} \end{array}\right. } \end{aligned}$$

where

$$\begin{aligned} R(n, p) = \frac{1 - \frac{n p}{n_0 p_0 \mu _n \mu _p}}{\tau _n \left( 1 + \frac{p}{p_0 \mu _p}\right) + \tau _p \left( 1 + \frac{n}{n_0 \mu _n}\right) }. \end{aligned}$$

Remarking that the entropy–entropy production inequality derived in Theorem 1.5 holds uniformly in the fast-reaction parameter $0<\varepsilon \le \varepsilon _0$, one intuitively expects the entropy method and the convergence result of Theorem 1.3 to extend to system (20). Here, we are interested to make this conjecture rigorous also in view of a better general understanding of the equilibration of systems which are derived as fast-reaction limits or quasi-steady-state approximations. One technical point is how to bypass the $\varepsilon$-dependency of the conservation law (3). The details of this singular limit are subject of the last Sect. 7. Altogether, we prove for system (20) the Theorems 7.3, 7.2 and Corollary 7.5 as corresponding versions of Theorems 1.3, 1.5 and Corollary 1.8.

Up to our knowledge, this is a first result in performing the entropy method in a non-linear reaction–diffusion-type system uniformly in a fast-reaction limit. Note that our approach yields global convergence to equilibrium for all initial data rather than just exponential stability of equilibria as proven, for instance, in a related 1D Poisson–Nernst–Planck system uniformly in the permittivity entering Poisson’s equation [17].

The rest of the paper is organised in the following manner. Section 2 proves the existence of a unique equilibrium (Theorem 2.1) as well as uniform-in-$\varepsilon$ bounds of $n_\infty$, $p_\infty$ and $n_{tr,\infty }$. In Sect. 3, we collect a couple of technical lemmata, and within Sect. 4, we state a preliminary proposition which serves as a first result towards an EEP-inequality. An abstract version of the EEP-estimate is proven in Sect. 5, first for constant concentrations and based on that also for general concentrations. Section 6 is concerned with the proofs of the EEP-inequality from Theorem 1.5, the announced exponential convergence from Theorem 1.3 and the uniform $L^\infty$-bounds from Corollary 1.8, whereas Sect. 7 is devoted to the same issues in the situation $\varepsilon \rightarrow 0$. Finally, the proof of Theorem 1.1 is contained in the Appendix.

## Properties of the equilibrium

We prove the existence of a unique positive equilibrium $(n_\infty , p_\infty , n_{tr,\infty })$ of system (1)–(3) in a suitable (and natural) function space. Note that uniqueness is only satisfied once the total charge *M* in (3) is fixed. This equilibrium can either be seen as the unique solution of the below stationary system (21) or as the unique state for which the entropy production (7) vanishes.

### Theorem 2.1

(Stationary system and uniformly bounded equilibrium) Let $M \in {\mathbb {R}}$, $\varepsilon \in (0,\varepsilon _0]$ for arbitrary $\varepsilon _0>0$ and $(n_\infty , p_\infty , n_{tr,\infty }) \in X$ where *X* is defined via

$$\begin{aligned} X:= & {} \left\{ (n, p, n_{tr}) \in H^1(\Omega )^2 \times L^\infty (\Omega ) \, \big | \, \overline{n} - \overline{p} + \varepsilon \overline{n_{tr}} = M \right. \\&\quad \left. \wedge\ (\exists \, \gamma > 0)\ n, p \ge \gamma \, \text{ a.e. } \wedge n_{tr} \in [\gamma , 1-\gamma ] \, \text{ a.e. }\right\} . \end{aligned}$$

Then, the following statements are equivalent.

1. $(n_\infty , p_\infty , n_{tr,\infty })\in X$ is a solution of the stationary system
  21a $$\begin{aligned} \nabla \cdot J_n(n_\infty ) + R_n(n_\infty ,n_{tr,\infty })&= 0, \end{aligned}$$
  21b $$\begin{aligned} \nabla \cdot J_p(p_\infty ) + R_p(p_\infty ,n_{tr,\infty })&= 0, \end{aligned}$$
  21c $$\begin{aligned} R_p(p_\infty ,n_{tr,\infty }) - R_n(n_\infty ,n_{tr,\infty })&= 0. \end{aligned}$$
2. $$P(n_\infty , p_\infty , n_{tr,\infty }) = 0$$.
3. $$J_n(n_\infty ) = J_p(p_\infty ) = R_n(n_\infty ,n_{tr,\infty }) = R_p(p_\infty ,n_{tr,\infty }) = 0$$ a.e. in $$\Omega$$.
4. The state $(n_\infty , p_\infty , n_{tr,\infty })$ satisfies
  22 $$\begin{aligned} n_\infty = n_*e^{-V_n}, \quad p_\infty = p_*e^{-V_p}, \quad n_{tr,\infty } = \frac{n_*}{n_*+ n_0} = \frac{p_0}{p_*+ p_0} \end{aligned}$$
  where the positive constants $n_*, p_*>0$ are uniquely determined by the condition
  23 $$\begin{aligned} n_*p_*= n_0 p_0 \end{aligned}$$
  and the conservation law
  24 $$\begin{aligned} n_*\overline{\mu _n} - p_*\overline{\mu _p} + \varepsilon \, n_{tr,\infty } = M. \end{aligned}$$

Consequently, the unique positive equilibrium $(n_\infty , p_\infty , n_{tr,\infty }) \in X$ is given by (22)–(24), and

25 $$\begin{aligned} n_ {tr,\infty } = \frac{n_*}{n_0}(1 - n_{tr,\infty }), \quad 1 - n_{tr,\infty } = \frac{p_*}{p_0} n_{tr,\infty }. \end{aligned}$$

Finally, for all $M\in {\mathbb {R}}$ and for $\varepsilon \in (0,\varepsilon _0]$, there exist two constants $\gamma \in (0, 1/2)$ and $\Gamma \in (1/2, \infty )$ depending only on $\varepsilon _0$, $n_0$, $p_0$, *M*, $V:= \max ( \Vert V_n \Vert _{L^\infty (\Omega )}, \Vert V_p \Vert _{L^\infty (\Omega )})$ such that

26 $$\begin{aligned}&n_\infty (x), p_\infty (x) \in [\gamma , \Gamma ] \quad \text {for a.e.\ } x \in \Omega \quad \text {and}\nonumber \\&n_*, p_*\in [\gamma , \Gamma ], \quad n_{tr,\infty } \in \left[ \gamma , 1-\gamma \right] . \end{aligned}$$

### Proof of Theorem 2.1

We shall prove the equivalence of the statements in the theorem by a circular reasoning. Assume that $(n_\infty , p_\infty , n_{tr,\infty }) \in X$ is a solution of the stationary system (21). In this case,

$$\begin{aligned} J_n(n_\infty ), \, J_p(p_\infty ), \, R_n(n_\infty ,n_{tr,\infty }), \, R_p(p_\infty ,n_{tr,\infty }) \in L^2(\Omega ). \end{aligned}$$

We test Eq. (21a) with $\ln (n_\infty /(n_0 \mu _n))$. Due to $n_\infty \in H^1(\Omega )$ and $n_\infty \ge \gamma$ a.e. in $\Omega$, the test function $\ln (n_\infty /(n_0 \mu _n))$ belongs to $H^1(\Omega )$. We find

$$\begin{aligned} 0 = \int _{\Omega } \left( \frac{|J_n(n_\infty )|^2}{n_\infty } - R_n(n_\infty ,n_{tr,\infty }) \ln \left( \frac{n_\infty }{n_0 \mu _n} \right) \right) dx. \end{aligned}$$

In the same way, we test Eq. (21b) with $\ln (p_\infty /(p_0 \mu _p)) \in H^1(\Omega )$. This yields

$$\begin{aligned} 0 = \int _{\Omega } \left( \frac{|J_p(p_\infty )|^2}{p_\infty } - R_p(p_\infty ,n_{tr,\infty }) \ln \left( \frac{p_\infty }{p_0 \mu _p} \right) \right) dx. \end{aligned}$$

Moreover, we multiply (21c) with $\ln (n_{tr,\infty }/(1-n_{tr,\infty })) \in L^2(\Omega )$, integrate over $\Omega$ and obtain

$$\begin{aligned} 0 = \int _{\Omega } \left( \left( R_n(n_\infty ,n_{tr,\infty }) - R_p(p_\infty ,n_{tr,\infty }) \right) \ln \left( \frac{n_{tr,\infty }}{1-n_{tr,\infty }} \right) \right) dx. \end{aligned}$$

Taking the sum of the three expressions above, we arrive at

$$\begin{aligned} P(n_\infty , p_\infty , n_{tr,\infty })= & {} \int _{\Omega } \Bigg (\frac{|J_n(n_\infty )|^2}{n_\infty } + \frac{|J_p(p_\infty )|^2}{p_\infty } \\&- \underbrace{R_n(n_\infty ,n_{tr,\infty }) \ln \left( \frac{n_\infty (1-n_{tr,\infty })}{n_0 \mu _n n_{tr,\infty }} \right) }_{\le 0} \\&- \underbrace{R_p(p_\infty ,n_{tr,\infty }) \ln \left( \frac{p_\infty n_{tr,\infty }}{p_0 \mu _p (1-n_{tr,\infty })} \right) }_{\le 0} \Bigg ) dx = 0. \end{aligned}$$

By the non-negativity of the terms in the last two lines, equality holds if and only if $R_n(n_\infty ,n_{tr,\infty }) = 0=R_p(p_\infty ,n_{tr,\infty })$. Hence, $P(n_\infty , p_\infty , n_{tr,\infty }) = 0$ readily implies $J_n(n_\infty ) = J_p(p_\infty ) = R_n(n_\infty ,n_{tr,\infty }) = R_p(p_\infty ,n_{tr,\infty }) = 0$ a.e. in $\Omega$.

Because of $J_n(n_\infty ) = \mu _n \nabla \bigl (\frac{n_\infty }{\mu _n}\bigr ) = 0 = J_p(p_\infty ) = \mu _p \nabla \bigl (\frac{p_\infty }{\mu _p}\bigr )$, we know that

$$\begin{aligned} n_\infty (x) = n_*e^{-V_n}, \quad p_\infty (x) = p_*e^{-V_p} \end{aligned}$$

with constants $n_*, p_*$. Moreover, $R_n(n_\infty ,n_{tr,\infty }) = R_p(p_\infty ,n_{tr,\infty }) = 0$ gives rise to

$$\begin{aligned} n_ {tr,\infty } = \frac{n_*}{n_0}(1 - n_{tr,\infty }), \quad 1 - n_{tr,\infty } = \frac{p_*}{p_0} n_{tr,\infty }. \end{aligned}$$

Consequently, $n_*p_*= n_0 p_0>0$, which implies $n_*, p_*>0$ and

$$\begin{aligned} n_{tr,\infty } = \frac{n_*}{n_*+ n_0} = \frac{p_0}{p_*+ p_0} \in (0,1). \end{aligned}$$

Moreover, for *M* fixed, the constants $n_*$ and $p_*$ are uniquely determined by the conservation law

$$\begin{aligned} n_*\overline{\mu _n} - p_*\overline{\mu _p} + \varepsilon \, n_{tr,\infty } = M, \end{aligned}$$

where the uniqueness follows from the strict monotonicity of $f(n_*) {:}{=}n_*\overline{\mu _n} - \frac{n_0 p_0 }{n_*}\overline{\mu _p} + \varepsilon \, \frac{n_*}{n_*+ n_0}$ on $(0, \infty )$ and the asymptotics $f(n_*) \rightarrow -\infty$ for $n_*\rightarrow 0^+$ and $f(n_*) \rightarrow \infty$ for $n_*\rightarrow \infty$.

Finally, concluding the circular reasoning $1. \rightarrow 2. \rightarrow 3. \rightarrow 4. \rightarrow 1.$, we observe that

$$\begin{aligned} n_\infty = n_*e^{-V_n}, \quad p_\infty = p_*e^{-V_p}, \quad n_{tr,\infty } = \frac{n_*}{n_*+ n_0} = \frac{p_0}{p_*+ p_0} \end{aligned}$$

obviously satisfies $J_n(n_\infty ) = J_p(p_\infty ) = R_n(n_\infty ,n_{tr,\infty }) = R_p(p_\infty ,n_{tr,\infty }) = 0$ a.e. in $\Omega$ which proves $(n_\infty , p_\infty , n_{tr, \infty })$ to be a solution of the stationary system.

In order to prove the bounds (26), we observe

$$\begin{aligned} n_*\overline{\mu _n} - \frac{n_0 p_0 }{n_*}\overline{\mu _p} = M - \varepsilon n_{tr,\infty } = M - \varepsilon \, \frac{n_*}{n_*+ n_0}, \end{aligned}$$

the left hand side is strictly monotone increasing from $-\infty$ to $+\infty$ as $n_*\in (0,\infty )$, while the right hand side is strictly monotone decreasing and bounded between $(M,M-\varepsilon _0)$ as $n_*\in (0,\infty )$. Both monotonicity and unboundedness/boundedness imply uniform positive lower and upper bounds for $n_*$ as explicitly proven in the following: First, we derive that

27 $$\begin{aligned} n_*= \frac{M - \varepsilon n_{tr,\infty }}{2 \overline{\mu _n}} + \sqrt{ \frac{(M - \varepsilon n_{tr,\infty })^2}{4 \overline{\mu _n}^2} + \frac{n_0 p_0 \overline{\mu _p}}{\overline{\mu _n}}} > 0 \end{aligned}$$

for all $\varepsilon \in (0,\varepsilon _0]$. Note that (27) is not an explicit representation of $n_*$ since $n_{tr,\infty }$ depends itself on $n_*$. Because of $n_{tr,\infty } \in (0, 1)$, we further observe that

$$\begin{aligned} n_*\le & {} \frac{|M - \varepsilon n_{tr,\infty }|}{2\overline{\mu _n}} + \sqrt{ \frac{(M - \varepsilon n_{tr,\infty })^2}{4\overline{\mu _n}^2}} + \sqrt{\frac{n_0 p_0 \overline{\mu _p}}{\overline{\mu _n}}} \\\le & {} \frac{|M| + \varepsilon _0}{\overline{\mu _n}} + \sqrt{\frac{n_0 p_0 \overline{\mu _p}}{\overline{\mu _n}}} \le \beta < \infty , \end{aligned}$$

where $\beta =\beta (\varepsilon _0,n_0,p_0,M,V)$. And as a result of the elementary inequality $\sqrt{a + b} \ge \sqrt{a} + \frac{b}{2 \sqrt{a} + \sqrt{b}}$ for $a \ge 0$ and $b > 0$, we also conclude that

$$\begin{aligned} n_*\ge & {} \frac{M - \varepsilon n_{tr,\infty }}{2 \overline{\mu _n}} + \frac{|M - \varepsilon n_{tr,\infty }|}{2 \overline{\mu _n}} + \frac{\frac{n_0 p_0 \overline{\mu _p}}{\overline{\mu _n}}}{\frac{|M - \varepsilon n_{tr,\infty }|}{\overline{\mu _n}} + \sqrt{\frac{n_0 p_0 \overline{\mu _p}}{\overline{\mu _n}}}} \\\ge & {} \frac{\frac{n_0 p_0 \overline{\mu _p}}{\overline{\mu _n}}}{\frac{|M| + \varepsilon _0}{\overline{\mu _n}} + \sqrt{\frac{n_0 p_0 \overline{\mu _p}}{\overline{\mu _n}}}} \ge \alpha > 0 \end{aligned}$$

where $\alpha =\alpha (\varepsilon _0,n_0,p_0,M,V)$. Similar arguments show that corresponding bounds $\alpha$ and $\beta$ are also available for $p_*$. Hence,

$$\begin{aligned} n_{tr,\infty } \in \left[ \frac{\alpha }{\alpha + n_0}, \frac{\beta }{\beta + n_0} \right] . \end{aligned}$$

Due to $n_\infty = n_*e^{-V_n}$, $p_\infty = p_*e^{-V_p}$ and the $L^\infty$-bounds on $V_n$ and $V_p$, the claim of the proposition follows. $\square$

## Some technical lemmata

A particularly useful relation between the concentrations *n*, *p* and $n_{tr}$ is the following Lemma.

### Lemma 3.1

The conservation law $\overline{n} - \overline{p} + \varepsilon \, \overline{n_{tr}} = M$ and the equilibrium condition (25) imply

28 $$\begin{aligned}&\forall \, t \ge 0: \quad (\overline{n} - \overline{n_\infty }) \ln \left( \frac{n_*}{n_0} \right) + (\overline{p} - \overline{p_\infty }) \ln \left( \frac{p_*}{p_0} \right) \nonumber \\&\quad = \varepsilon (\overline{n_{tr}} - n_{tr,\infty }) \ln \left( \frac{1-n_{tr,\infty }}{n_{tr,\infty }} \right) . \end{aligned}$$

### Proof

With $\overline{n_\infty } - \overline{p_\infty } + \varepsilon \, n_{tr,\infty } = M$ (note that $n_{tr,\infty } = \overline{n_{tr,\infty }}$ is constant), we have $\overline{p} - \overline{p_\infty } = \overline{n} - \overline{n_\infty } + \varepsilon (\overline{n_{tr}} - n_{tr,\infty })$. We employ this relation to replace $\overline{p} - \overline{p_\infty }$ on the left hand side of (28) and calculate

$$\begin{aligned}&(\overline{n} - \overline{n_\infty }) \ln \left( \frac{n_*}{n_0} \right) + (\overline{p} - \overline{p_\infty }) \ln \left( \frac{p_*}{p_0} \right) \\&\quad = (\overline{n} - \overline{n_\infty }) \ln \left( \frac{n_*p_*}{n_0 p_0} \right) + \varepsilon (\overline{n_{tr}} - n_{tr,\infty }) \ln \left( \frac{p_*}{p_0} \right) . \end{aligned}$$

Now, the first term on the right hand side vanishes due to $n_*p_*= n_0 p_0$ while we use $p_*/p_0 = (1-n_{tr,\infty }) / n_{tr,\infty }$ for the second term and obtain

$$\begin{aligned} (\overline{n} - \overline{n_\infty }) \ln \left( \frac{n_*}{n_0} \right) + (\overline{p} - \overline{p_\infty }) \ln \left( \frac{p_*}{p_0} \right) = \varepsilon (\overline{n_{tr}} - n_{tr,\infty }) \ln \left( \frac{1-n_{tr,\infty }}{n_{tr,\infty }} \right) \end{aligned}$$

as claimed above. $\square$

### Lemma 3.2

(Relative Entropy) The entropy relative to the equilibrium reads

$$\begin{aligned}&E(n,p,n_{tr}) - E(n_\infty , p_\infty , n_{tr,\infty }) \\&\quad = \int _{\Omega } \left( n \ln \frac{n}{n_\infty } - (n-n_\infty ) + p \ln \frac{p}{p_\infty } - (p-p_\infty ) \right. \\&\qquad \left. + \varepsilon \int _{n_{tr,\infty }}^{n_{tr}(x)} \left( \ln \left( \frac{s}{1-s} \right) - \ln \left( \frac{n_{tr,\infty }}{1-n_{tr,\infty }} \right) \right) ds \right) dx. \end{aligned}$$

### Proof

By the definition of $E(n,p,n_{tr})$ in (5), we note that

$$\begin{aligned}&E(n,p,n_{tr}) - E(n_\infty , p_\infty , n_{tr,\infty }) \\&\quad = \int _\Omega \bigg ( n \ln \left( \frac{n}{n_0 \mu _n} \right) - n_\infty \ln \left( \frac{n_\infty }{n_0 \mu _n} \right) - (n - n_\infty ) \\&\qquad + p \ln \left( \frac{p}{p_0 \mu _p} \right) - p_\infty \ln \left( \frac{p_\infty }{p_0 \mu _p} \right) - (p - p_\infty ) \\&\qquad + \varepsilon \int _{n_{tr,\infty }}^{n_{tr}(x)} \ln \left( \frac{s}{1-s} \right) ds \bigg ) dx. \end{aligned}$$

We expand the first integrand as $n \ln \bigl ( \frac{n}{n_0 \mu _n} \bigr ) = n \ln \bigl ( \frac{n}{n_\infty } \bigr ) + n \ln \bigl ( \frac{n_\infty }{n_0 \mu _n} \bigr ).$ Thus, with $n_\infty / \mu _n = n_*$, we get

$$\begin{aligned}&\int _\Omega \bigg ( n \ln \left( \frac{n}{n_0 \mu _n} \right) - n_\infty \ln \left( \frac{n_\infty }{n_0 \mu _n} \right) - (n - n_\infty ) \bigg ) dx \\&\quad = \int _\Omega \bigg ( n \ln \left( \frac{n}{n_\infty } \right) - (n - n_\infty ) \bigg ) dx + (\overline{n} - \overline{n_\infty }) \ln \left( \frac{n_*}{n_0} \right) . \end{aligned}$$

Together with an analogous calculation of the *p*-terms, we obtain

$$\begin{aligned}&E(n,p,n_{tr}) - E(n_\infty , p_\infty , n_{tr,\infty }) \\&\quad = \int _\Omega \bigg ( n \ln \left( \frac{n}{n_\infty } \right) - (n - n_\infty ) + p \ln \left( \frac{p}{p_\infty } \right) - (p - p_\infty ) \bigg ) dx \\&\qquad + (\overline{n} - \overline{n_\infty }) \ln \left( \frac{n_*}{n_0} \right) + (\overline{p} - \overline{p_\infty }) \ln \left( \frac{p_*}{p_0} \right) \\&\qquad + \varepsilon \int _\Omega \int _{n_{tr,\infty }}^{n_{tr}(x)} \ln \left( \frac{s}{1-s} \right) ds \, dx. \end{aligned}$$

Lemma 3.1 allows us to reformulate the last two lines as

$$\begin{aligned}&(\overline{n} - \overline{n_\infty }) \ln \left( \frac{n_*}{n_0} \right) + (\overline{p} - \overline{p_\infty }) \ln \left( \frac{p_*}{p_0} \right) + \varepsilon \int _\Omega \int _{n_{tr,\infty }}^{n_{tr}(x)} \ln \left( \frac{s}{1-s} \right) ds \, dx \\&\quad = \varepsilon (\overline{n_{tr}} - n_{tr,\infty }) \ln \left( \frac{1-n_{tr,\infty }}{n_{tr,\infty }} \right) + \varepsilon \int _\Omega \int _{n_{tr,\infty }}^{n_{tr}(x)} \ln \left( \frac{s}{1-s} \right) ds \, dx \\&\quad = \varepsilon \int _\Omega \int _{n_{tr,\infty }}^{n_{tr}(x)} \left( \ln \left( \frac{s}{1-s} \right) - \ln \left( \frac{n_{tr,\infty }}{1 - n_{tr,\infty }} \right) \right) ds \, dx, \end{aligned}$$

which proves the claim. $\square$

### Lemma 3.3

(Csiszár–Kullback–Pinsker inequality) Let $f, g: \Omega \rightarrow {\mathbb {R}}$ be non-negative measurable functions. Then,

$$\begin{aligned} \int _\Omega \left( f \ln \Big ( \frac{f}{g} \Big ) - (f - g) \right) dx \ge \frac{3}{2 \overline{f} + 4 \overline{g}} \Vert f - g \Vert _{L^1(\Omega )}^2. \end{aligned}$$

### Proof

Following a proof by Pinsker, we start with the elementary inequality $3 (x-1)^2 \le (2x + 4) (x \ln x - (x-1))$. This allows us to derive the following Csiszár–Kullback–Pinsker-type inequality:

$$\begin{aligned} \Vert f - g \Vert _{L^1(\Omega )}&= \int _\Omega g \left| \frac{f}{g} - 1\right| \, dx \le \int _\Omega g \sqrt{\frac{2}{3} \frac{f}{g} + \frac{4}{3}} \, \sqrt{\frac{f}{g} \ln \Big ( \frac{f}{g} \Big ) - \Big (\frac{f}{g} - 1 \Big )} \, dx \\&= \int _\Omega \sqrt{\frac{2}{3} f + \frac{4}{3} g} \, \sqrt{f \ln \Big ( \frac{f}{g} \Big ) - (f - g)} \, dx \\&\le \sqrt{\frac{2}{3} \overline{f} + \frac{4}{3} \overline{g}} \, \sqrt{\int _\Omega \left( f \ln \Big ( \frac{f}{g} \Big ) - (f - g) \right) dx} \end{aligned}$$

where we applied Hölder’s inequality in the last step. $\square$

The subsequent lemma provides $L^1$-bounds for *n* and *p* in terms of the initial entropy of the system and some further constants.

### Lemma 3.4

($L^1$-bounds) Due to the monotonicity of the entropy functional, any entropy producing solution of (1) satisfies

$$\begin{aligned} \forall \, t \ge 0: \quad \overline{n}, \, \overline{p} \le \frac{5}{2} \max \{ n_0 \overline{\mu _n}, p_0 \overline{\mu _p} \} + \frac{3}{4} E(n(0), p(0), n_{tr}(0)) =: M_1. \end{aligned}$$

### Proof

Employing Lemma 3.3 and Young’s inequality, we find

$$\begin{aligned} \overline{n}&\le n_0 \overline{\mu _n} + \Vert n - n_0 \mu _n \Vert _{L^1(\Omega )} \\&\le n_0 \overline{\mu _n} + \sqrt{\frac{2}{3} \overline{n} + \frac{4}{3} n_0 \overline{\mu _n}} \, \sqrt{\int _\Omega \left( n \ln \Big ( \frac{n}{n_0 \mu _n} \Big ) - (n - n_0 \mu _n) \right) dx} \\&\le n_0 \overline{\mu _n} + \frac{1}{3} \overline{n} + \frac{2}{3} n_0 \overline{\mu _n} + \frac{1}{2} \int _\Omega \left( n \ln \Big ( \frac{n}{n_0 \mu _n} \Big ) - (n - n_0 \mu _n) \right) dx. \end{aligned}$$

Solving this inequality for $\overline{n}$ yields

$$\begin{aligned} \overline{n} \le \frac{5}{2} n_0 \overline{\mu _n} + \frac{3}{4} \int _\Omega \left( n \ln \left( \frac{n}{n_0 \mu _n} \right) - (n - n_0 \mu _n) \right) dx. \end{aligned}$$

Therefore, we arrive at

$$\begin{aligned} \overline{n} \le \frac{5}{2} n_0 \overline{\mu _n} + \frac{3}{4} E(n, p, n_{tr}) \le \frac{5}{2} \max \{n_0 \overline{\mu _n}, p_0 \overline{\mu _p}\} + \frac{3}{4} E(n(0), p(0), n_{tr}(0)) \end{aligned}$$

where we used the monotonicity of the entropy functional in the last step. In the same way, we may bound $\overline{p}$ from above. $\square$

At certain points, we will have to estimate the difference between terms like $\overline{n/n_\infty }$ and $\overline{n} / \overline{n_{\infty }}$. Using Lemma 3.5 below, we can bound this difference by the $J_n$-flux term and, hence, by the entropy production.

### Lemma 3.5

Let $f \in L^1(\Omega )$ and $g \in L^\infty (\Omega )$ such that $f \ge 0$, $g \ge \gamma > 0$ a.e. on $\Omega$ and *f*/*g* is weakly differentiable. Then, there exists an explicit constant $C(\Vert f\Vert _{L^1(\Omega )}, \Vert g\Vert _{L^\infty (\Omega )}, \gamma ) > 0$ such that

$$\begin{aligned} \bigg ( \frac{\overline{f}}{\overline{g}} - \overline{\left( \frac{f}{g} \right) } \bigg )^2 \le C \int _\Omega \left| \nabla \sqrt{ \frac{f}{g} } \, \right| ^2 dx. \end{aligned}$$

### Proof

We define $\delta := \frac{f}{g} - \overline{\left( \frac{f}{g} \right) }$ and obtain $f = g \left( \overline{\left( \frac{f}{g} \right) } + \delta \right)$ and

$$\begin{aligned} \frac{\overline{f}}{\overline{g}} = \int _\Omega \frac{f}{\overline{g}} \, dx = \int _\Omega \frac{g}{\overline{g}} \left( \overline{\left( \frac{f}{g} \right) } + \delta \right) dx = \overline{\left( \frac{f}{g} \right) } + \int _\Omega \frac{g}{\overline{g}} \, \delta \, dx \end{aligned}$$

by utilising $|\Omega | = 1$. Therefore,

$$\begin{aligned} \left| \frac{\overline{f}}{\overline{g}} - \overline{\left( \frac{f}{g} \right) } \right| \le \frac{\Vert g\Vert _{L^\infty (\Omega )}}{\overline{g}} \Vert \delta \Vert _{L^1(\Omega )} \le C_P \frac{\Vert g\Vert _{L^\infty (\Omega )}}{\overline{g}} \left\| \nabla \left( \frac{f}{g} \right) \right\| _{L^1(\Omega )} \end{aligned}$$

by applying Poincaré’s inequality to $\delta$ with $\overline{\delta }= 0$ and some constant $C_P(\Omega ) > 0$. As $g \ge \gamma > 0$ is uniformly positive on $\Omega$ and $\overline{g} \ge \gamma$, we arrive at

$$\begin{aligned} \left| \frac{\overline{f}}{\overline{g}} - \overline{\left( \frac{f}{g} \right) } \right| \le C_P \frac{\Vert g\Vert _{L^\infty (\Omega )}}{\gamma ^2} \left\| g \, \nabla \left( \frac{f}{g} \right) \right\| _{L^1(\Omega )}. \end{aligned}$$

Finally, we deduce

$$\begin{aligned}&\left( \frac{\overline{f}}{\overline{g}} - \overline{\left( \frac{f}{g} \right) } \right) ^2 \le \left( C_P \frac{\Vert g\Vert _{L^\infty (\Omega )}}{\gamma ^2} \right) ^2 \left\| \sqrt{fg} \sqrt{\frac{g}{f}} \nabla \left( \frac{f}{g} \right) \right\| _{L^1(\Omega )}^2 \\&\quad \le 4 \overline{fg} \left( C_P \frac{\Vert g\Vert _{L^\infty (\Omega )}}{\gamma ^2} \right) ^2 \int _\Omega \left| \nabla \sqrt{ \frac{f}{g} } \, \right| ^2 dx \end{aligned}$$

employing Hölder’s inequality in the second step. $\square$

## Two preliminary propositions

### Notation 4.1

For arbitrary functions *f*, we define the normalised quantity

$$\begin{aligned} \widetilde{f} := \frac{\, f \,}{\overline{f}}. \end{aligned}$$

The following logarithmic Sobolev inequality on bounded domains was proven in [6] by following an argument of Stroock [22].

### Lemma 4.2

(Logarithmic Sobolev inequality on bounded domains) Let $\Omega$ be a bounded domain in ${\mathbb {R}}^{m}$, $m \ge 1$, such that the Poincaré (–Wirtinger) and Sobolev inequalities

29 $$\begin{aligned}&\Vert \phi - \int _{\Omega } \phi \, dx\Vert _{L^2(\Omega )}^2 \le P(\Omega ) \, \Vert \nabla \phi \Vert _{L^2(\Omega )}^2, \end{aligned}$$

30 $$\begin{aligned}&\Vert \phi \Vert _{L^q(\Omega )}^2 \le C_1(\Omega ) \, \Vert \nabla \phi \Vert _{L^2(\Omega )}^2 + C_2(\Omega )\, \Vert \phi \Vert _{L^2(\Omega )}^2, \quad \frac{1}{q} = \frac{1}{2}-\frac{1}{m},\quad m\ge 3, \end{aligned}$$

hold with $q=\infty$ for $m=1$ and any $q<\infty$ for $m=2$. Then, the logarithmic Sobolev inequality

31 $$\begin{aligned} \int _{\Omega } \phi ^2 \ln \left( \frac{\phi ^2}{\Vert \phi \Vert _{L^2(\Omega )}^2}\right) dx \le L(\Omega ,m) \, \Vert \nabla \phi \Vert _{L^2(\Omega )}^2 \end{aligned}$$

holds (for some constant $L(\Omega ,m)>0$).

The Log-Sobolev inequality allows to bound an appropriate part of the entropy functional by the flux parts of the entropy production. The normalised variables on the left hand side of the subsequent inequality naturally arise when reformulating the flux terms on the right hand side in such a way that we can apply the Log-Sobolev inequality on $\Omega$.

### Proposition 4.3

Recall the assumptions $\Vert V_n\Vert _{L^\infty (\Omega )}, \Vert V_p\Vert _{L^\infty (\Omega )}\le V$. Then, there exists a constant $C(V)>0$ such that

$$\begin{aligned} \int _{\Omega } \left( n \ln \bigg ( \frac{\widetilde{n}}{\widetilde{\mu _n}} \bigg ) + p \ln \bigg ( \frac{\widetilde{p}}{\widetilde{\mu _p}} \bigg ) \right) dx \le C \int _{\Omega } \left( \frac{|J_n|^2}{n} + \frac{|J_p|^2}{p} \right) dx. \end{aligned}$$

### Proof

From the definition of $J_n$ one obtains

$$\begin{aligned} \int _{\Omega } \frac{|J_n|^2}{n} \, dx= & {} \int _{\Omega } \frac{\mu _n}{n} \left| \nabla \left( \frac{n}{\mu _n}\right) \right| ^2 \mu _n \, dx = 4 \, \overline{n} \int _{\Omega } \frac{\mu _n}{\overline{n}} \left| \nabla \sqrt{\frac{n}{\mu _n}}\right| ^2 \, dx \\= & {} 4\, \overline{n} \int _{\Omega } \frac{\mu _n}{\overline{\mu _n}} \left| \nabla \sqrt{\frac{\widetilde{n}}{\widetilde{\mu _n}}}\right| ^2 \, dx. \end{aligned}$$

We set

$$\begin{aligned} \phi (x) := \sqrt{\frac{\widetilde{n}}{\widetilde{\mu _n}}}, \quad \alpha {:}{=}\int _{\Omega } \phi (x)^2 \, dx \end{aligned}$$

and observe that $\alpha =\frac{\overline{\mu _n}}{\overline{n}} \int _{\Omega } \frac{n}{\mu _n}\,dx \le \overline{\mu _n} e^V$ is bounded independently of *n*. Next, we introduce the rescaled variable $y {:}{=}\alpha ^{-\frac{1}{m}} x$ where $m \ge 1$ denotes the space dimension. Note that $\Vert \phi \Vert _{L^2(dx)}$ is in general different from one, whereas $\Vert \phi \Vert _{L^2(dy)} = 1$. We now estimate with $\Vert V_n\Vert _{L^\infty (\Omega )}\le V$ and the logarithmic Sobolev inequality (31)

$$\begin{aligned} \int _{\Omega } | \nabla _{\!x} \phi |^2 \, dx= & {} \int _{\Omega } | \alpha ^{-\frac{1}{m}} \nabla _{\!y} \phi |^2 \, \alpha \, dy = \alpha ^{1 - \frac{2}{m}} \int _{\Omega } | \nabla _{\!y} \phi |^2 \, dy \\\ge & {} \alpha ^{1 - \frac{2}{m}} \frac{1}{L} \int _{\Omega } \phi ^2 \ln ( \phi ^2) \, dy =\alpha ^{1 - \frac{2}{m}} \frac{1}{L} \int _{\Omega } \frac{\widetilde{n}}{\widetilde{\mu _n}} \ln \left( \frac{\widetilde{n}}{\widetilde{\mu _n}} \right) \, dy \\= & {} \alpha ^{-\frac{2}{m}} \frac{1}{L} \frac{\overline{\mu _n}}{\overline{n}} \int _{\Omega } \frac{n}{\mu _n} \ln \left( \frac{\widetilde{n}}{\widetilde{\mu _n}} \right) \, dx. \end{aligned}$$

The corresponding estimate involving $J_n$ reads

$$\begin{aligned} \int _{\Omega } \frac{|J_n|^2}{n} \, dx&\ge 4\, \frac{\overline{n}}{\overline{\mu _n}} e^{-V} \int _{\Omega } \left| \nabla _{\!x} \phi \right| ^2 \, dx \ge \frac{4}{L} \alpha ^{-\frac{2}{m}} e^{-2V} \int _{\Omega } n \ln \Big ( \frac{\widetilde{n}}{\widetilde{\mu _n}} \Big ) \, dx. \end{aligned}$$

The same arguments apply to the terms involving *p*. $\square$

The following proposition contains the first step towards an entropy–entropy production inequality. The relative entropy can be controlled by the flux part of the entropy production and three additional terms, which mainly consist of square roots of averaged quantities. The proof that the entropy production also serves as an upper bound for these terms will be the subject of the next section.

### Proposition 4.4

There exists an explicit constant $C(\gamma , \Gamma , M_1) > 0$ such that for $(n_\infty , p_\infty , n_{tr,\infty }) \in X$ from Theorem 2.1 and all non-negative functions $(n,p,n_{tr}) \in L^1(\Omega )^3$ satisfying $n_{tr} \le 1$, the conservation law

$$\begin{aligned} \overline{n} - \overline{p} + \varepsilon \overline{n_{tr}} = M \end{aligned}$$

and the $L^1$-bound

$$\begin{aligned} \overline{n}, \overline{p} \le M_1, \end{aligned}$$

the following estimate holds true:

32 $$\begin{aligned}&E(n,p,n_{tr}) - E(n_\infty , p_\infty , n_{tr,\infty }) \le C \, \left( \int _\Omega \left( \frac{|J_n|^2}{n} + \frac{|J_p|^2}{p} \right) dx \right. \nonumber \\&\quad \left. + \left( \sqrt{\overline{\left( \frac{n}{\mu _n}\right) }} - \sqrt{n_*} \right) ^2 + \left( \sqrt{\overline{\left( \frac{p}{\mu _p}\right) }} - \sqrt{p_*} \right) ^2 + \varepsilon \int _\Omega \left( \sqrt{n_{tr}} - \sqrt{n_{tr,\infty }} \right) ^2 \, dx \right) . \end{aligned}$$

(Note that the right hand side of (32) vanishes at the equilibrium $(n_\infty , p_\infty , n_{tr,\infty })$.)

### Proof

According to Lemma 3.2, we have

$$\begin{aligned}&E(n,p,n_{tr}) - E(n_\infty , p_\infty , n_{tr,\infty }) \\&\quad = \int _{\Omega } \left( n \ln \frac{n}{n_\infty } - (n-n_\infty ) + p \ln \frac{p}{p_\infty } - (p-p_\infty ) \right. \\&\qquad \left. + \varepsilon \int _{n_{tr,\infty }}^{n_{tr}} \left( \ln \Big ( \frac{s}{1-s} \Big ) - \ln \Big ( \frac{n_{tr,\infty }}{1-n_{tr,\infty }} \Big ) \right) ds \right) dx. \end{aligned}$$

Recall that $n = \widetilde{n}\,\overline{n}$, $n_\infty = \widetilde{n_\infty }\,\overline{n_\infty }$ and $\widetilde{n_\infty } = \widetilde{\mu _n}$. Using these relations, we rewrite the first two integrands as

$$\begin{aligned} n \ln \Big ( \frac{n}{n_\infty } \Big ) - (n - n_\infty ) = n \ln \Big ( \frac{\widetilde{n}}{\widetilde{\mu _n}} \Big ) + n \ln \Big ( \frac{\overline{n}}{\overline{n_\infty }} \Big ) - (n - n_\infty ) \end{aligned}$$

and analogously for the *p*-terms. This results in

33 $$\begin{aligned}&E(n,p,n_{tr}) - E(n_\infty , p_\infty , n_{tr,\infty }) \nonumber \\&\quad = \int _\Omega \bigg ( n \ln \Big ( \frac{\widetilde{n}}{\widetilde{\mu _n}} \Big ) + p \ln \Big ( \frac{\widetilde{p}}{\widetilde{\mu _p}} \Big ) \bigg ) dx \nonumber \\&\qquad + \, \overline{n_\infty } \left( \frac{\overline{n}}{\overline{n_\infty }} \ln \Big ( \frac{\overline{n}}{\overline{n_\infty }} \Big ) - \Big ( \frac{\overline{n}}{\overline{n_\infty }} - 1 \Big ) \right) \nonumber \\&\qquad + \overline{p_\infty } \left( \frac{\overline{p}}{\overline{p_\infty }} \ln \Big ( \frac{\overline{p}}{\overline{p_\infty }} \Big ) - \Big ( \frac{\overline{p}}{\overline{p_\infty }} - 1 \Big ) \right) \nonumber \\&\qquad + \varepsilon \int _\Omega \int _{n_{tr,\infty }}^{n_{tr}} \left( \ln \Big ( \frac{s}{1-s} \Big ) - \ln \Big ( \frac{n_{tr,\infty }}{1-n_{tr,\infty }} \Big ) \right) ds \, dx. \end{aligned}$$

The terms in the second line of (33) can be estimated using the Log-Sobolev inequality of Proposition 4.3. Moreover, the elementary inequality $x \ln x - (x-1) \le (x-1)^2$ for $x>0$ gives rise to

$$\begin{aligned}&\overline{n_\infty } \left( \frac{\overline{n}}{\overline{n_\infty }} \ln \Big ( \frac{\overline{n}}{\overline{n_\infty }} \Big ) - \Big ( \frac{\overline{n}}{\overline{n_\infty }} - 1 \Big ) \right) \le \overline{n_\infty } \left( \frac{\overline{n}}{\overline{n_\infty }} - 1 \right) ^2 \\&\quad \le 2 \overline{n_\infty } \left[ \bigg ( \overline{\Big (\frac{n}{n_\infty }\Big )} - 1 \bigg )^2 + \bigg ( \frac{\overline{n}}{\overline{n_\infty }} - \overline{\Big (\frac{n}{n_\infty }\Big )} \bigg )^2 \right] \end{aligned}$$

and an analogous estimate for the corresponding expressions involving *p*. The second term on the right hand side of the previous line can be bounded from above by applying Lemma 3.5, which guarantees a constant $C(\gamma , \Gamma , M_1) > 0$ such that

$$\begin{aligned}&\bigg ( \frac{\overline{n}}{\overline{n_\infty }} - \overline{\Big (\frac{n}{n_\infty }\Big )} \bigg )^2 \le C \int _\Omega \left| \nabla \sqrt{ \frac{n}{n_\infty } } \right| ^2 dx \\&\quad \le \frac{C}{4 \inf _\Omega n_\infty } \int _\Omega \frac{1}{n} \left| n_\infty \, \nabla \Big ( \frac{n}{n_\infty } \Big ) \right| ^2 dx \le c_1 \int _\Omega \frac{|J_n|^2}{n} \, dx \end{aligned}$$

for some constant $c_1(\gamma , \Gamma , M_1) > 0$. Besides,

$$\begin{aligned} \bigg ( \overline{\Big (\frac{n}{n_\infty }\Big )} - 1 \bigg )^2= & {} \frac{1}{n_*^2} \bigg ( \overline{\Big (\frac{n}{\mu _n}\Big )} - n_*\bigg )^2 = \frac{1}{n_*^2} \left( \sqrt{\overline{\Big (\frac{n}{\mu _n}\Big )}} + \sqrt{n_*} \right) ^2 \left( \sqrt{\overline{\Big (\frac{n}{\mu _n}\Big )}} - \sqrt{n_*} \right) ^2 \\= & {} \frac{1}{n_*} \left( \sqrt{\overline{\Big (\frac{n}{n_\infty }\Big )}} + 1 \right) ^2 \left( \sqrt{\overline{\Big (\frac{n}{\mu _n}\Big )}} - \sqrt{n_*} \right) ^2 \\\le & {} C(\gamma , M_1) \left( \sqrt{\overline{\Big (\frac{n}{\mu _n}\Big )}} - \sqrt{n_*} \right) ^2. \end{aligned}$$

See (26) and Lemma 3.4 for the bounds on $n_*$, $n_\infty$ and $\overline{n}$. We have thus verified that

$$\begin{aligned} \overline{n_\infty } \left( \frac{\overline{n}}{\overline{n_\infty }} \ln \Big ( \frac{\overline{n}}{\overline{n_\infty }} \Big ) - \Big ( \frac{\overline{n}}{\overline{n_\infty }} - 1 \Big ) \right) \le c_2 \left( \int _\Omega \frac{|J_n|^2}{n} \, dx + \bigg ( \sqrt{\overline{\Big (\frac{n}{\mu _n}\Big )}} - \sqrt{n_*} \bigg )^2 \right) \end{aligned}$$

with some $c_2(\gamma , \Gamma , M_1) > 0$. A similar estimate holds true for the corresponding part of (33) involving *p*.

Considering the last line in (33), we further know that for all $x \in \Omega$ there exists some mean value

$$\begin{aligned} \theta (x) \in \left( \min \{n_{tr}(x), n_{tr,\infty }\}, \max \{n_{tr}(x), n_{tr,\infty }\}\right) \end{aligned}$$

such that

34 $$\begin{aligned} \int _{n_{tr,\infty }}^{n_{tr}(x)} \ln \left( \frac{s}{1-s} \right) ds = \ln \left( \frac{\theta (x)}{1-\theta (x)} \right) (n_{tr}(x) - n_{tr,\infty }). \end{aligned}$$

Consequently,

$$\begin{aligned} \varepsilon \int _\Omega \int _{n_{tr,\infty }}^{n_{tr}(x)} \ln \left( \frac{s}{1-s} \right) ds \, dx = \varepsilon \int _\Omega \ln \left( \frac{\theta (x)}{1-\theta (x)} \right) \left( n_{tr}(x) - n_{tr,\infty }\right) \, dx. \end{aligned}$$

In fact, we will prove that there even exists some constant $\xi \in (0,1/2)$ such that

$$\begin{aligned} \theta (x) \in (\xi , 1-\xi ) \end{aligned}$$

for all $x \in \Omega$. Thus, the function $\theta (x)$ is uniformly bounded away from 0 and 1 on $\Omega$. To see this, we first note that $n_{tr,\infty } \in [\gamma , 1-\gamma ]$ using the constant $\gamma \in (0, 1/2)$ from (26). In addition,

$$\begin{aligned} \left| \int _{n_{tr,\infty }}^{n_{tr}(x)} \ln \left( \frac{s}{1-s} \right) ds \right| \le \int _{0}^1 \left| \ln \left( \frac{s}{1-s} \right) \right| ds = 2\ln (2) \end{aligned}$$

for all $x \in \Omega$. Together with (34), this estimate implies

$$\begin{aligned} \left| \ln \left( \frac{\theta (x)}{1-\theta (x)} \right) \right| \big | n_{tr}(x) - n_{tr,\infty } \big | \le 2 \ln (2). \end{aligned}$$

We now choose an arbitrary $x \in \Omega$ and distinguish two cases. If $| n_{tr}(x) - n_{tr,\infty } | \ge \gamma /2$, then

$$\begin{aligned} \left| \ln \left( \frac{\theta (x)}{1-\theta (x)} \right) \right| \le \frac{2\ln (2)}{| n_{tr}(x) - n_{tr,\infty } |} \le \frac{4\ln (2)}{\gamma }. \end{aligned}$$

As a consequence of $\ln (s/(1-s)) \rightarrow \infty$ for $s \rightarrow 1^-$ and $\ln (s/(1-s)) \rightarrow -\infty$ for $s \rightarrow 0^+$, there exists some constant $\xi \in (0,\gamma )$ depending only on $\gamma$ such that $\theta (x) \in (\xi , 1-\xi )$. If $| n_{tr}(x) - n_{tr,\infty } | < \gamma /2$, then $n_{tr,\infty } \in [\gamma , 1 - \gamma ]$ implies $n_{tr}(x) \in (\gamma /2, 1-\gamma /2)$ and, hence, $\theta (x) \in (\gamma /2, 1-\gamma /2)$. Again the constant $\xi$ depends only on $\gamma$.

As a result of the calculations above, we may rewrite the last line in (33) as

$$\begin{aligned}&\varepsilon \int _\Omega \int _{n_{tr,\infty }}^{n_{tr}(x)} \left( \ln \left( \frac{s}{1-s} \right) - \ln \left( \frac{n_{tr,\infty }}{1-n_{tr,\infty }} \right) \right) ds \, dx \\&\quad = \varepsilon \int _\Omega \left( \ln \left( \frac{\theta (x)}{1-\theta (x)} \right) - \ln \left( \frac{n_{tr,\infty }}{1-n_{tr,\infty }} \right) \right) (n_{tr}(x) - n_{tr,\infty }) \, dx. \end{aligned}$$

Applying the mean-value theorem to the expression in brackets and observing that

$$\begin{aligned} \frac{d}{ds} \ln \left( \frac{s}{1-s} \right) = \frac{1}{s(1-s)}, \end{aligned}$$

we find

$$\begin{aligned}&\varepsilon \int _\Omega \left( \ln \left( \frac{\theta (x)}{1-\theta (x)} \right) - \ln \left( \frac{n_{tr,\infty }}{1-n_{tr,\infty }} \right) \right) (n_{tr}(x) - n_{tr,\infty }) \, dx \\&\quad = \varepsilon \int _\Omega \frac{1}{\sigma (x)(1-\sigma (x))} (\theta (x) - n_{tr,\infty }) (n_{tr}(x) - n_{tr,\infty }) \, dx \end{aligned}$$

with some $\sigma (x) \in (\min \{\theta (x), n_{tr,\infty }\}, \max \{\theta (x), n_{tr,\infty }\})$. Since both $\theta (x), n_{tr,\infty } \in (\xi , 1-\xi )$ for all $x \in \Omega$, we also know that $\sigma (x) \in (\xi , 1-\xi )$ for all $x \in \Omega$. Thus, $(\sigma (x)(1-\sigma (x)))^{-1}$ is bounded uniformly in $\Omega$ in terms of $\xi = \xi (\gamma )$. Consequently,

$$\begin{aligned}&\varepsilon \int _\Omega \int _{n_{tr,\infty }}^{n_{tr}(x)} \left( \ln \left( \frac{s}{1-s} \right) - \ln \left( \frac{n_{tr,\infty }}{1-n_{tr,\infty }} \right) \right) ds \, dx \\&\quad \le \varepsilon c_3 \int _\Omega |\theta (x) - n_{tr,\infty }| |n_{tr}(x) - n_{tr,\infty }| \, dx \le \varepsilon c_3 \int _\Omega (n_{tr} - n_{tr,\infty })^2 \, dx \\&\quad = \varepsilon c_3 \int _\Omega \left( \sqrt{n_{tr}} + \sqrt{n_{tr,\infty }}\right) ^2 \left( \sqrt{n_{tr}} - \sqrt{n_{tr,\infty }}\right) ^2 \, dx \le 4 \varepsilon c_3 \int _\Omega \left( \sqrt{n_{tr}} - \sqrt{n_{tr,\infty }} \right) ^2 \, dx \end{aligned}$$

with a constant $c_3(\gamma ) > 0$ after applying the estimate $|\theta (x) - n_{tr,\infty }| \le |n_{tr}(x) - n_{tr,\infty }|$ for all $x \in \Omega$. Finally, we arrive at

$$\begin{aligned}&E(n,p,n_{tr}) - E(n_\infty , p_\infty , n_{tr,\infty }) \le C \, \left( \int _\Omega \left( \frac{|J_n|^2}{n} + \frac{|J_p|^2}{p} \right) dx\right. \\&\quad \left. + \left( \sqrt{\overline{\left( \frac{n}{\mu _n}\right) }} - \sqrt{n_*} \right) ^2 + \left( \sqrt{\overline{\left( \frac{p}{\mu _p}\right) }} - \sqrt{p_*} \right) ^2 + \varepsilon \int _\Omega \left( \sqrt{n_{tr}} - \sqrt{n_{tr,\infty }} \right) ^2 \, dx \right) \end{aligned}$$

with a constant $$C(\gamma , \Gamma , M_1) > 0$$. $$\square$$

## Abstract versions of the EEP-inequality

### Notation 5.1

We set

$$\begin{aligned} n_{tr}' := 1 - n_{tr}, \quad n_{tr, \infty }' := 1 - n_{tr, \infty } \end{aligned}$$

and define the positive constants

$$\begin{aligned} \nu _\infty:= & {} \sqrt{\frac{n_*}{n_0}} = \sqrt{\frac{n_\infty }{n_0 \mu _n}}, \quad \pi _\infty := \sqrt{\frac{p_*}{p_0}} = \sqrt{\frac{p_\infty }{p_0 \mu _p}}, \\ \nu _{tr, \infty }:= & {} \sqrt{n_{tr, \infty }}, \quad \nu _{tr, \infty }' := \sqrt{n_{tr, \infty }'}. \end{aligned}$$

The motivation for introducing the additional variable $n_{tr}'$ is the possibility to symmetrise expressions like $(n(1-n_{tr}) - n_{tr})^2 + (pn_{tr} - (1-n_{tr}))^2$ as $(nn_{tr}' - n_{tr})^2 + (pn_{tr} - n_{tr}')^2$. Similar terms will appear frequently within the subsequent calculations.

### Remark 5.2

We may consider $n_{tr}'$ as a fourth independent variable within our model. In this case, the reaction–diffusion system features the following two independent conservation laws:

$$\begin{aligned} \overline{n} - \overline{p} + \varepsilon \,\overline{n_{tr}}= & {} n_0 \, \overline{\mu _n \left( \frac{n}{n_0 \mu _n}\right) } - p_0 \, \overline{\mu _p \left( \frac{p}{p_0 \mu _p}\right) } + \varepsilon \, \overline{n_{tr}} = M \in {\mathbb {R}}, \\ n_{tr}(x) + n_{tr}'(x)= & {} 1 \ \text{ for } \text{ all } \ x \in \Omega . \end{aligned}$$

The special formulation of the first conservation law will become clear when looking at the following two Propositions. There, we derive relations for general variables *a*, *b*, *c* and *d*, which correspond to $\sqrt{n/(n_0 \mu _n)}$, $\sqrt{p/(p_0 \mu _p)}$, $\sqrt{n_{tr}}$ and $\sqrt{n_{tr}'}$, respectively.

In addition, we have the following $L^1$-bound (cf. Lemma 3.4):

$$\begin{aligned} \overline{n}, \overline{p} \le M_1. \end{aligned}$$

The following Proposition 5.3 establishes an upper bound for the terms in the second line of (32) in the case of *constant* concentrations *a*, *b*, *c* and *d*. This result is then generalised in Proposition 5.5 to *non-constant* states *a*, *b*, *c*, *d*.

### Proposition 5.3

(Homogeneous concentrations) Let $a, b, c, d \ge 0$ be constants such that their squares satisfy the conservation laws

$$\begin{aligned} n_0 \overline{\mu _n} a^2 - p_0 \overline{\mu _p} b^2 + \varepsilon \, c^2= & {} M = n_0 \overline{\mu _n} \nu _\infty ^2 - p_0 \overline{\mu _p} \pi _\infty ^2 + \varepsilon \, \nu _{tr,\infty }^2, \\ c^2 + d^2= & {} 1 = \nu _{tr,\infty }^2 + \nu _{tr,\infty }'^{\, 2} \end{aligned}$$

for any $\varepsilon \in (0,\varepsilon _0]$ and arbitrary $\varepsilon _0>0$. Moreover, assume

$$\begin{aligned} a^2, b^2 \le C(n_0, p_0, M_1, V). \end{aligned}$$

Then, there exists an explicitly computable constant $C(\varepsilon _0, n_0, p_0, M, M_1, V) > 0$ such that

35 $$\begin{aligned} (a - \nu _{\infty })^2 + (b - \pi _\infty )^2 + (c - \nu _{tr,\infty })^2 \le C \left( (ad - c)^2 + (bc - d)^2 \right) \end{aligned}$$

for all $$\varepsilon \in (0,\varepsilon _0]$$.

### Proof

We first introduce the following change of variable: Due to the non-negativity of the concentrations *a*, *b*, *c*, *d*, we define constants $\mu _1, \mu _2, \mu _3, \mu _4 \in [-1,\infty )$ such that

$$\begin{aligned} a = \nu _\infty (1 + \mu _1), \quad b = \pi _\infty (1 + \mu _2), \quad c = \nu _{tr,\infty } (1 + \mu _3), \quad d = \nu _{tr,\infty }' (1 + \mu _4), \end{aligned}$$

where $\nu _\infty$, $\pi _\infty$, $\nu _{tr,\infty }$ and $\nu _{tr,\infty }'$ are uniformly positive and bounded for all $\varepsilon \in (0,\varepsilon _0]$ in terms of $\varepsilon _0, n_0, p_0, M$ and *V* by (26). Thus, the boundedness of *a*, *b*, *c*, *d* implies the existence of a constant $K(\varepsilon _0, n_0, p_0, M, M_1, V)>0$, such that $\mu _i \in [-1,K]$ for all $1 \le i \le 4$. The left hand side of (35) expressed in terms of the $\mu _i$ rewrites as

$$\begin{aligned} (a - \nu _{\infty })^2 + (b - \pi _\infty )^2 + (c - \nu _{tr,\infty })^2 = \nu _\infty ^2 \mu _1^2 + \pi _\infty ^2 \mu _2^2 + \nu _{tr,\infty }^2 \mu _3^2. \end{aligned}$$

Employing the equilibrium conditions (25), we also find

$$\begin{aligned} ad - c= & {} \nu _\infty \nu _{tr,\infty }' (1 + \mu _1)(1 + \mu _4) - \nu _{tr,\infty }(1 + \mu _3) \\= & {} \nu _{tr,\infty } \left[ (1+\mu _1)(1+\mu _4) - (1+\mu _3) \right] \end{aligned}$$

and

$$\begin{aligned} bc - d= & {} \pi _\infty \nu _{tr,\infty } (1 + \mu _2)(1 + \mu _3) - \nu _{tr,\infty }'(1 + \mu _4) \\= & {} \nu _{tr,\infty }' \left[ (1+\mu _2)(1+\mu _3) - (1+\mu _4) \right] . \end{aligned}$$

Moreover, the two conservation laws from the hypotheses rewrite as

36 $$\begin{aligned} n_0 \overline{\mu _n} \nu _\infty ^2 \mu _1 (2 + \mu _1) - p_0 \overline{\mu _p} \pi _\infty ^2 \mu _2 (2 + \mu _2) + \varepsilon \, \nu _{tr,\infty }^2 \mu _3(2 + \mu _3)&= 0, \end{aligned}$$

37 $$\begin{aligned} \nu _{tr,\infty }^2 \mu _3 (2 + \mu _3) + \nu _{tr,\infty }'^{\, 2} \mu _4 (2 + \mu _4)&= 0. \end{aligned}$$

The relations (36), (37) allow to express $\varepsilon \mu _3$ and $\varepsilon \mu _4$ in terms of $\mu _1$ and $\mu _2$, although not explicitly:

38 $$\begin{aligned} \varepsilon \mu _3&= - \frac{n_0 \overline{\mu _n} \nu _\infty ^2}{\nu _{tr,\infty }^2} \frac{2 + \mu _1}{2 + \mu _3}\, \mu _1 + \frac{p_0 \overline{\mu _p} \pi _\infty ^2}{\nu _{tr,\infty }^2} \frac{2 + \mu _2}{2 + \mu _3} \, \mu _2 \nonumber \\&=: - f_{1,3}(\mu _1, \mu _3) \mu _1 + f_{2,3}(\mu _2, \mu _3) \mu _2, \end{aligned}$$

39 $$\begin{aligned} \varepsilon \mu _4&= - \frac{\nu _{tr,\infty }^2}{\nu _{tr,\infty }'^{\, 2}} \frac{2 + \mu _3}{2 + \mu _4} \,\varepsilon \mu _3 =: - f_{3,4}(\mu _3, \mu _4)\, \varepsilon \mu _3 \nonumber \\&=: f_{1,4}(\mu _1, \mu _3,\mu _4) \mu _1 - f_{2,4}(\mu _2, \mu _3, \mu _4) \mu _2, \end{aligned}$$

where the last definition follows from inserting the previous expression (38) for $\varepsilon \mu _3$ while the factor $2+\mu _3$ is bounded in $[1,K+2]$ since $\mu _i \in [-1,K]$ for all $1 \le i \le 4$. Therefore, all the terms $f_{i,j}$ are uniformly positive as well as bounded from above:

$$\begin{aligned} 0&< \underline{C_{1,3}} \le f_{1,3} \le \overline{C_{1,3}}< \infty , \quad 0< \underline{C_{2,3}} \le f_{2,3} \le \overline{C_{2,3}}< \infty , \\ 0&< \underline{C_{3,4}} \le f_{3,4} \le \overline{C_{3,4}}< \infty , \quad 0< \underline{C_{1,4}} \le f_{1,4} \le \overline{C_{1,4}}< \infty , \\ 0&< \underline{C_{2,4}} \le f_{2,4} \le \overline{C_{2,4}} < \infty . \end{aligned}$$

All constants $\underline{C_{i,j}}$ and $\overline{C_{i,j}}$ only depend on $\varepsilon _0$, $n_0$, $p_0$, *M*, $M_1$ and *V*, and there exist corresponding bounds ${\underline{C}} > 0$ and $\overline{C} > 0$ such that for all *i*, *j*

$$\begin{aligned} {\underline{C}} \le \underline{C_{i,j}}, \overline{C_{i,j}} \le \overline{C}. \end{aligned}$$

In order to prove (35), we show that under the constraints of the conservation laws (36), (37), respectively, the relations (38), (39), there exists a constant $C(\varepsilon _0, n_0, p_0, M, {\underline{C}}, \overline{C}) > 0$ for all $\varepsilon \in (0,\varepsilon _0]$ such that

$$\begin{aligned} \frac{(a - \nu _{\infty })^2 + (b - \pi _\infty )^2 + (c - \nu _{tr,\infty })^2}{(ad - c)^2 + (bc - d)^2} \le C, \end{aligned}$$

which is equivalent to

40 $$\begin{aligned} \frac{\nu _\infty ^2 \mu _1^2 + \pi _\infty ^2 \mu _2^2 + \nu _{tr,\infty }^2 \mu _3^2}{\nu _{tr,\infty }^2 \left[ (1+\mu _1)(1+\mu _4) - (1+\mu _3) \right] ^2 + \nu _{tr,\infty }'^{\, 2} \left[ (1+\mu _2)(1+\mu _3) - (1+\mu _4) \right] ^2} \le C. \end{aligned}$$

Recall that $\nu _\infty ^2 \le \Gamma /n_0$, $\pi _\infty ^2 \le \Gamma /p_0$ and $\nu _{tr,\infty }^2, \nu _{tr,\infty }'^{\, 2} \in [\gamma , 1-\gamma ]$ with $\gamma \in (0, 1/2)$ and $\Gamma \in (1/2, \infty )$ depending on $\varepsilon _0$, $n_0$, $p_0$ and *M* for all $\varepsilon \in (0,\varepsilon _0]$ (cf. the proof of Proposition 26). Since numerator and denominator of (40) are sums of quadratic terms, it is sufficient to bound the denominator from below in terms of its numerator omitting the prefactors $\nu _\infty ^2$, $\pi _\infty ^2$, $\nu _{tr,\infty }^2$ and $\nu _{tr,\infty }'^{\,2}$, i.e. to prove that

41 $$\begin{aligned} (*):= & {} \left[ (1+\mu _1)(1+\mu _4) - (1+\mu _3) \right] ^2 + \left[ (1+\mu _2)(1+\mu _3) - (1+\mu _4) \right] ^2 \nonumber \\\ge & {} C \left( \mu _1^2 + \mu _2^2 + \mu _3^2\right) . \end{aligned}$$

More precisely, we will prove that there exists a constant $c(\varepsilon _0, {\underline{C}}, \overline{C}) > 0$ for all $\varepsilon \in (0,\varepsilon _0]$ such that

$$\begin{aligned} (*) = \left( \mu _1 + \mu _4 + \mu _1 \mu _4 - \mu _3 \right) ^2 + \left( \mu _2 + \mu _3 + \mu _2 \mu _3 - \mu _4 \right) ^2 \ge c \,(\mu _1^2 + \mu _2^2) \end{aligned}$$

and that

$$\begin{aligned} (*) = \left( \mu _1 + \mu _4 + \mu _1 \mu _4 - \mu _3 \right) ^2 + \left( \mu _2 + \mu _3 + \mu _2 \mu _3 - \mu _4 \right) ^2 \ge \mu _3^2. \end{aligned}$$

For this reason, we distinguish four cases and we shall frequently use estimates like

$$\begin{aligned} \mu _i + \mu _i \mu _j = \mu _i (1 + \mu _j) \ge 0 \quad \text{ iff } \quad \mu _i \ge 0 \quad \text {for all} \quad 1 \le j \le 4, \end{aligned}$$

since $\mu _j \ge -1$ for all $1 \le j \le 4$. We mention already here that all subsequent constants $c_1$, $c_2$ are strictly positive and depend only on $\varepsilon _0$, ${\underline{C}}$ and $\overline{C}$ uniformly for $\varepsilon \in (0,\varepsilon _0]$.

- *Case 1*: $\mu _1 \ge 0 \wedge \mu _2 \ge 0$: If $\mu _3 \ge 0$, then (37) implies $\mu _4 \le 0$ and $\mu _2 + \mu _3 + \mu _2 \mu _3 - \mu _4 \ge \mu _2$. Moreover, $\mu _3 \ge 0$ yields
  $$\begin{aligned} f_{2,3} \mu _2 \ge f_{1,3} \mu _1 \ \Rightarrow \ \overline{C_{2,3}} \mu _2 \ge \underline{C_{1,3}} \mu _1 \ \Rightarrow \ \mu _2 \ge \underline{C_{1,3}} / \overline{C_{2,3}} \, \mu _1 \end{aligned}$$
  and
  $$\begin{aligned} \mu _2 + \mu _3 + \mu _2 \mu _3 - \mu _4 \ge \mu _2 \ge \mu _2 / 2 + \underline{C_{1,3}} / ( 2 \, \overline{C_{2,3}} ) \mu _1 \ge c_1 (\mu _1 + \mu _2). \end{aligned}$$
  Hence, $(*) \ge \left( \mu _2 + \mu _3 + \mu _2 \mu _3 - \mu _4 \right) ^2 \ge c_2 (\mu _1^2 + \mu _2^2)$. Besides, $(*) \ge \left( \mu _2 + \mu _3 + \mu _2 \mu _3 - \mu _4 \right) ^2 \ge \mu _3^2$. If $\mu _3 < 0$, (37) yields $\mu _4 > 0$ and $\mu _1 + \mu _4 + \mu _1 \mu _4 - \mu _3 \ge \mu _1$. Since $\mu _3 < 0$, (38) implies
  $$\begin{aligned} f_{1,3} \mu _1 \ge f_{2,3} \mu _2 \ \Rightarrow \ \overline{C_{1,3}} \mu _1 \ge \underline{C_{2,3}} \mu _2 \ \Rightarrow \ \mu _1 \ge \underline{C_{2,3}} / \overline{C_{1,3}} \, \mu _2 \end{aligned}$$
  and
  $$\begin{aligned} \mu _1 + \mu _4 + \mu _1 \mu _4 - \mu _3 \ge \mu _1 \ge \mu _1 / 2 + \underline{C_{2,3}} / ( 2 \, \overline{C_{1,3}} ) \mu _2 \ge c_1 (\mu _1 + \mu _2). \end{aligned}$$
  As above, $(*) \ge c_2 (\mu _1^2 + \mu _2^2)$. The signs $\mu _3 \le 0 \le \mu _1,\mu _4$ yield $(*) \ge \left( \mu _1 + \mu _4 + \mu _1 \mu _4 - \mu _3 \right) ^2 \ge \mu _3^2$.
- *Case 2*: $\mu _1 \ge 0 \wedge \mu _2 < 0$: Equations (38) and (39) imply $\mu _3 \le 0$ and $\mu _4 \ge 0$, and we deduce for all $\varepsilon \in (0,\varepsilon _0]$
  $$\begin{aligned}&\mu _1 + \mu _4 + \mu _1 \mu _4 - \mu _3 \ge \mu _4 - \mu _3 \\&\quad = \varepsilon ^{-1}(f_{1,3} + f_{1,4}) \mu _1 - \varepsilon ^{-1}(f_{2,3} + f_{2,4}) \mu _2 \\&\quad \ge \varepsilon _0^{-1}(\underline{C_{1,3}} + \underline{C_{1,4}}) |\mu _1| + \varepsilon _0^{-1}(\underline{C_{2,3}} + \underline{C_{2,4}}) |\mu _2| \ge c_1 (|\mu _1| + |\mu _2|) \end{aligned}$$
  and, thus, $(*) \ge (\mu _1 + \mu _4 + \mu _1 \mu _4 - \mu _3)^2 \ge c_2 (\mu _1^2 + \mu _2^2)$. Since $\mu _2, \mu _3 \le 0 \le \mu _4$, we have
  $$\begin{aligned} (*) \ge \left( \mu _4 - \mu _3 - \mu _2 (1 + \mu _3) \right) ^2 \ge \mu _3^2. \end{aligned}$$
- *Case 3*: $\mu _1 < 0 \wedge \mu _2 \ge 0$: Here, $\mu _3 \ge 0$ due to (38) and, thus, $\mu _4 \le 0$ by (39), which yields for all $\varepsilon \in (0,\varepsilon _0]$
  $$\begin{aligned}&\mu _2 + \mu _3 + \mu _2 \mu _3 - \mu _4 \ge \mu _3 - \mu _4 = \varepsilon ^{-1}(f_{2,3} + f_{2,4}) \mu _2 - \varepsilon ^{-1}(f_{1,3} + f_{1,4}) \mu _1 \\&\quad \ge \varepsilon _0^{-1}(\underline{C_{1,3}} + \underline{C_{1,4}}) |\mu _1| + \varepsilon _0^{-1}(\underline{C_{2,3}} + \underline{C_{2,4}}) |\mu _2| \ge c_1 (|\mu _1| + |\mu _2|) \end{aligned}$$
  and $(*) \ge (\mu _2 + \mu _3 + \mu _2 \mu _3 - \mu _4)^2 \ge c_2 (\mu _1^2 + \mu _2^2)$. And as $\mu _1,\mu _4 \le 0\le \mu _3$, one has
  $$\begin{aligned} (*) \ge \left( \mu _3 - \mu _4 - \mu _1 (1 + \mu _4) \right) ^2 \ge \mu _3^2. \end{aligned}$$
- *Case 4*: $\mu _1< 0 \wedge \mu _2 < 0$: Supposing that $\mu _3 \ge 0$ and thus $\mu _4 \le 0$ by (39), we observe
  $$\begin{aligned} |\mu _1 + \mu _4 + \mu _1 \mu _4 - \mu _3| = \mu _3 - \mu _1 - \mu _4(1 + \mu _1) \ge -\mu _1. \end{aligned}$$
  Furthermore, $\mu _3 \ge 0$ enables us to estimate
  $$\begin{aligned} f_{1,3} \mu _1 \le f_{2,3} \mu _2 \ \Rightarrow \ \overline{C_{1,3}} \mu _1 \le \underline{C_{2,3}} \mu _2 \ \Rightarrow \ -\mu _1 \ge -\underline{C_{2,3}} / \overline{C_{1,3}} \, \mu _2. \end{aligned}$$
  and
  $$\begin{aligned} |\mu _1 + \mu _4 + \mu _1 \mu _4 - \mu _3| \ge -\mu _1 \ge -\mu _1 / 2 - \underline{C_{2,3}} / ( 2 \, \overline{C_{1,3}} ) \mu _2 \ge c_1 (|\mu _1| + |\mu _2|). \end{aligned}$$
  Hence, $(*) \ge (\mu _1 + \mu _4 + \mu _1 \mu _4 - \mu _3)^2 \ge c_2 (\mu _1^2 + \mu _2^2)$. The second estimate in terms of $\mu _3^2$ follows with $\mu _1, \mu _4 \le 0 \le \mu _3$ from
  $$\begin{aligned} (*) \ge \left( \mu _3 - \mu _4 - \mu _1 (1 + \mu _4) \right) ^2 \ge \mu _3^2. \end{aligned}$$
  In the opposite case that $\mu _3 < 0$ and thus $\mu _4 \ge 0$ due to (39), we estimate
  $$\begin{aligned} |\mu _2 + \mu _3 + \mu _2 \mu _3 - \mu _4| = \mu _4 - \mu _2 - \mu _3 (1+\mu _2) \ge -\mu _2 \end{aligned}$$
  and
  $$\begin{aligned} f_{2,3} \mu _2 \le f_{1,3} \mu _1 \ \Rightarrow \ \overline{C_{2,3}} \mu _2 \le \underline{C_{1,3}} \mu _1 \ \Rightarrow \ -\mu _2 \ge -\underline{C_{1,3}} / \overline{C_{2,3}} \, \mu _1. \end{aligned}$$
  We, thus, arrive at
  $$\begin{aligned} |\mu _2 + \mu _3 + \mu _2 \mu _3 - \mu _4| \ge -\mu _2 \ge -\mu _2 / 2 - \underline{C_{1,3}} / ( 2 \, \overline{C_{2,3}} ) \mu _1 \ge c_1 (|\mu _1| + |\mu _2|) \end{aligned}$$
  and $(*) \ge (\mu _2 + \mu _3 + \mu _2 \mu _3 - \mu _4)^2 \ge c_2 (\mu _1^2 + \mu _2^2)$. The corresponding inequality for $\mu _3$ reads
  $$\begin{aligned} (*) \ge \left( \mu _4 - \mu _3 - \mu _2 (1 + \mu _3) \right) ^2 \ge \mu _3^2, \end{aligned}$$
  which follows from $\mu _2,\mu _3 \le 0 \le \mu _4$.

The proof of the proposition is now complete. $\square$

### Notation 5.4

From now on, $\Vert \cdot \Vert$ without further specification shall always denote the $L^2$-norm in $\Omega$.

Within the subsequent Proposition 5.5, the expressions $(ad-c)^2$ and $(bc-d)^2$ on the right hand side of (35) will be generalised to $\Vert ad-c\Vert ^2$ and $\Vert bc-d\Vert ^2$ in (42). We will later show in the proof of Theorem 1.5 that $\Vert ad-c\Vert ^2$ (and also $\Vert bc-d\Vert ^2$) can be estimated from above via the reaction terms within the entropy production (7) when using the special choices $\sqrt{n/(n_0 \mu _n)}$, $\sqrt{p/(p_0 \mu _p)}$, $\sqrt{n_{tr}}$, and $\sqrt{n_{tr}'}$ for *a*, *b*, *c*, and *d*.

### Proposition 5.5

(Inhomogeneous concentrations) Let $a, b, c, d: \Omega \rightarrow {\mathbb {R}}$ be measurable, non-negative functions such that their squares satisfy the conservation laws

$$\begin{aligned} n_0 \overline{\mu _n a^2} - p_0 \overline{\mu _p b^2} + \varepsilon \, \overline{c^2}= & {} M = n_0 \overline{\mu _n} \nu _\infty ^2 - p_0 \overline{\mu _p} \pi _\infty ^2 + \varepsilon \, \nu _{tr,\infty }^2, \\ \overline{c^2} + \overline{d^2}= & {} 1 = \nu _{tr,\infty }^2 + \nu _{tr,\infty }'^{\, 2} \end{aligned}$$

for any $\varepsilon \in (0,\varepsilon _0]$ and arbitrary $\varepsilon _0>0$. In addition, we assume

$$\begin{aligned} \overline{a^2}, \, \overline{b^2} \le C(n_0, p_0, M_1, V). \end{aligned}$$

Then, there exists an explicitly computable constant $C(\varepsilon _0, n_0, p_0, M, M_1, V) > 0$ such that

42 $$\begin{aligned}&\left( \sqrt{\overline{a^2}} - \nu _\infty \right) ^2 + \left( \sqrt{\overline{b^2}} - \pi _\infty \right) ^2 + \Vert c - \nu _{tr,\infty } \Vert ^2 \nonumber \\&\quad \le C \, \left( \Vert ad - c \Vert ^2 + \Vert bc - d \Vert ^2 + \Vert \nabla a \Vert ^2 + \Vert \nabla b \Vert ^2 \right. \nonumber \\&\qquad \left. + \Vert a - \overline{a} \Vert ^2 + \Vert b - \overline{b} \Vert ^2 + \Vert c - \overline{c} \Vert ^2 + \Vert d - \overline{d} \Vert ^2 \right) \end{aligned}$$

for all $$\varepsilon \in (0,\varepsilon _0]$$.

### Proof

We divide the proof into two steps. In the first part, we shall derive lower bounds for the reaction terms $\Vert ad - c \Vert ^2 + \Vert bc - d \Vert ^2$ involving $( \overline{a} \, \overline{d} - \overline{c} )^2 + ( \overline{b} \, \overline{c} - \overline{d} )^2$. This will allow us to apply Proposition 5.3 in the second step.

*Step 1:* We show

$$\begin{aligned} \Vert ad - c \Vert ^2 \ge \frac{1}{2} \big (\overline{a} \, \overline{d} - \overline{c} \big )^2 - c_1 \left( \Vert a - \overline{a} \Vert ^2 + \Vert b - \overline{b} \Vert ^2 + \Vert c - \overline{c} \Vert ^2 + \Vert d - \overline{d} \Vert ^2 \right) , \end{aligned}$$

and

$$\begin{aligned} \Vert bc - d \Vert ^2 \ge \frac{1}{2} \big (\overline{b} \, \overline{c} - \overline{d} \big )^2 - c_1 \left( \Vert a - \overline{a} \Vert ^2 + \Vert b - \overline{b} \Vert ^2 + \Vert c - \overline{c} \Vert ^2 + \Vert d - \overline{d} \Vert ^2 \right) \end{aligned}$$

with some explicitly computable constant $c_1>0$. For this reason, we define

$$\begin{aligned} \delta _1 := a - \overline{a}, \quad \delta _2 := b - \overline{b}, \quad \delta _3 := c - \overline{c}, \quad \delta _4 := d - \overline{d} \end{aligned}$$

and note that $\overline{\delta _1} = \overline{\delta _2} = \overline{\delta _3} = \overline{\delta _4} = 0$. Moreover,

$$\begin{aligned} | \overline{a} \, \overline{d} - \overline{c} |, \, | \overline{b} \, \overline{c} - \overline{d} | \le C(n_0, p_0, M_1, V) \end{aligned}$$

due to Young’s inequality, $\overline{a^2}$, $\overline{b^2} \le C(n_0, p_0, M_1, V)$ and $\overline{c^2}$, $\overline{d^2} \le 1$.

We now define

$$\begin{aligned} S := \big \{ x \in \Omega \, \big | \, |\delta _1| \le 1 \wedge |\delta _2| \le 1 \wedge |\delta _3| \le 1 \wedge |\delta _4| \le 1 \big \} \end{aligned}$$

and split the squares of the $L^2(\Omega )$-norm as

43 $$\begin{aligned} \Vert ad - c \Vert ^2 = \int _S (ad - c)^2 \, dx + \int _{\Omega \backslash S} (ad - c)^2 \, dx \end{aligned}$$

and

$$\begin{aligned} \Vert bc - d \Vert ^2 = \int _S (bc - d)^2 \, dx + \int _{\Omega \backslash S} (bc - d)^2 \, dx, \end{aligned}$$

respectively. In order to estimate the first integral in (43) from below, we write

$$\begin{aligned} ad = (\overline{a} + \delta _1)(\overline{d} + \delta _4) = \overline{a} \overline{d} + \overline{a} \delta _4 + \overline{d} \delta _1 + \delta _1 \delta _4, \quad c = \overline{c} + \delta _3. \end{aligned}$$

This yields

$$\begin{aligned} \int _S (ad - c)^2 \, dx= & {} \int _S (\overline{a} \overline{d} - \overline{c})^2 \, dx + 2 \int _S (\overline{a} \overline{d} - \overline{c})(\overline{a} \delta _4 + \overline{d} \delta _1 + \delta _1 \delta _4 - \delta _3) \, dx \\&+ \int _S (\overline{a} \delta _4 + \overline{d} \delta _1 + \delta _1 \delta _4 - \delta _3)^2 \, dx \\\ge & {} \frac{1}{2} \int _S (\overline{a} \overline{d} - \overline{c})^2 \, dx - \int _S (\overline{a} \delta _4 + \overline{d} \delta _1 + \delta _1 \delta _4 - \delta _3)^2 \, dx\\\ge & {} \frac{1}{2} \int _S (\overline{a} \overline{d} - \overline{c})^2 \, dx - C(n_0, p_0, M_1, V) \left( \overline{\delta _1^2} + \overline{\delta _3^2} + \overline{\delta _4^2} \right) \end{aligned}$$

where we used Young’s inequality $2xy \ge -x^2/2 - 2y^2$ for $x,y \in {\mathbb {R}}$ in the second step and the boundedness of $\delta _i$, $1 \le i \le 4$, in the last step. Similarly, we deduce

$$\begin{aligned} \int _S (bc - d)^2 \, dx \ge \frac{1}{2} \int _S (\overline{b} \overline{c} - \overline{d})^2 \, dx - C(n_0, p_0, M_1, V) \left( \overline{\delta _2^2} + \overline{\delta _3^2} + \overline{\delta _4^2} \right) . \end{aligned}$$

The second integral in (43) is mainly estimated by deriving an upper bound for the measure of $\Omega \backslash S$. For all $i \in \{1,\ldots ,4\}$ we have

$$\begin{aligned} \left| \left\{ \delta _i^2> 1 \right\} \right| = \int _{\left\{ \delta _i^2 > 1 \right\} } 1 \, dx \le \int _{\Omega }\delta _i^2 \, dx = \overline{\delta _i^2} \end{aligned}$$

and, hence,

$$\begin{aligned} |\Omega \backslash S| \le \sum _{i=1}^4 \left| \left\{ \delta _i^2 > 1 \right\} \right| \le \overline{\delta _1^2} + \overline{\delta _2^2} + \overline{\delta _3^2} + \overline{\delta _4^2}. \end{aligned}$$

As a consequence of $| \overline{a} \, \overline{d} - \overline{c} | \le C(n_0, p_0, M_1, V)$, we obtain

$$\begin{aligned} \int _{\Omega \backslash S} ( \overline{a} \, \overline{d} - \overline{c} )^2 \, dx\le & {} C(n_0, p_0, M_1, V) \, |\{\Omega \backslash S\}|\nonumber \\\le & {} C(n_0, p_0, M_1, V) \left( \overline{\delta _1^2} + \overline{\delta _2^2} + \overline{\delta _3^2} + \overline{\delta _4^2} \right) . \end{aligned}$$

This implies

$$\begin{aligned} \int _{\Omega \backslash S} (ad - c)^2 \, dx\ge & {} 0 \ge \frac{1}{2} \int _{\Omega \backslash S} ( \overline{a} \, \overline{d} - \overline{c} )^2 \, dx \nonumber \\&- C(n_0, p_0, M_1, V) \left( \overline{\delta _1^2} + \overline{\delta _2^2} + \overline{\delta _3^2} + \overline{\delta _4^2} \right) \end{aligned}$$

and, analogously,

$$\begin{aligned} \int _{\Omega \backslash S} (bc - d)^2 \, dx \ge 0\ge & {} \frac{1}{2} \int _{\Omega \backslash S} ( \overline{b} \, \overline{c} - \overline{d} )^2 \, dx \nonumber \\&- C(n_0, p_0, M_1, V) \left( \overline{\delta _1^2} + \overline{\delta _2^2} + \overline{\delta _3^2} + \overline{\delta _4^2} \right) . \end{aligned}$$

Taking the sum of both contributions to (43), we finally arrive at

44 $$\begin{aligned} \Vert ad-c\Vert ^2 \ge \frac{1}{2} \big (\overline{a} \, \overline{d} - \overline{c} \big )^2 - c_1(n_0, p_0, M_1, V) \left( \overline{\delta _1^2} + \overline{\delta _2^2} + \overline{\delta _3^2} + \overline{\delta _4^2} \right) \end{aligned}$$

and

45 $$\begin{aligned} \Vert bc-d\Vert ^2 \ge \frac{1}{2} \big (\overline{b} \, \overline{c} - \overline{d} \big )^2 - c_1(n_0, p_0, M_1, V) \left( \overline{\delta _1^2} + \overline{\delta _2^2} + \overline{\delta _3^2} + \overline{\delta _4^2} \right) . \end{aligned}$$

*Step 2:* We introduce constants $\mu _i \ge -1$, $1 \le i \le 4$, such that

$$\begin{aligned} \overline{a^2}= & {} \nu _\infty ^2 (1 + \mu _1)^2, \quad \overline{b^2} = \pi _\infty ^2 (1 + \mu _2)^2, \nonumber \\ \overline{c^2}= & {} \nu _{tr,\infty }^2 (1 + \mu _3)^2, \quad \overline{d^2} = \nu _{tr,\infty }'^{\, 2} (1 + \mu _4)^2. \end{aligned}$$

We recall that (26) guarantees the uniform positivity and boundedness of $\nu _\infty$, $\pi _\infty$, $\nu _{tr,\infty }$, and $\nu _{tr,\infty }'$ for all $\varepsilon \in (0, \varepsilon _0]$ in terms of $\varepsilon _0$, $n_0$, $p_0$, *M*, and *V*. Therefore, the bounds $\overline{a^2}$, $\overline{b^2} \le C(n_0, p_0, M_1, V)$ and $\overline{c^2}$, $\overline{d^2} \le 1$ give rise to a constant $K(\varepsilon _0, n_0, p_0, M, M_1, V) > 0$ such that $\mu _i \in [-1,K]$ for all $1 \le i \le 4$ uniformly for $\varepsilon \in (0, \varepsilon _0]$.

We now want to derive a formula for $\overline{a}$ in terms of $\delta _1$ and $\mu _1$. Since $\overline{a^2} - \overline{a}^2 = \Vert a - \overline{a}\Vert ^2 = \Vert \delta _1\Vert ^2 = \overline{\delta _1^2}$, one finds

46 $$\begin{aligned} \overline{a} = \sqrt{\overline{a^2}} - \frac{\overline{\delta _1^2}}{\sqrt{\overline{a^2}} + \overline{a}} = \nu _\infty (1 + \mu _1) - \frac{\overline{\delta _1^2}}{\sqrt{\overline{a^2}} + \overline{a}} \end{aligned}$$

and analogous expressions for $\overline{b}$, $\overline{c}$ and $\overline{d}$:

$$\begin{aligned} \overline{b} &= \pi _\infty (1 + \mu _2) - \frac{\overline{\delta _2^2}}{\sqrt{\overline{b^2}} + \overline{b}}, \quad \overline{c} = \nu _{tr,\infty } (1 + \mu _3) - \frac{\overline{\delta _3^2}}{\sqrt{\overline{c^2}} + \overline{c}}, \\ \overline{d} &= \nu _{tr,\infty }' (1 + \mu _4) - \frac{\overline{\delta _4^2}}{\sqrt{\overline{d^2}} + \overline{d}}. \end{aligned}$$

Furthermore,

$$\begin{aligned} \left( \sqrt{\overline{a^2}} - \nu _\infty \right) ^{2} = \nu _\infty ^2 \mu _1^2, \quad \left( \sqrt{\overline{b^2}} - \pi _\infty \right) ^{2} = \pi _\infty ^2 \mu _2^2 \end{aligned}$$

and, similarly,

$$\begin{aligned} \Vert c - \nu _{tr,\infty } \Vert ^2 &= \overline{c^2} - 2 \overline{c} \nu _{tr,\infty } + \nu _{tr,\infty }^2 \\ &= \nu _{tr,\infty }^2 (1 + \mu _3)^2 - 2 \nu _{tr,\infty }^2 (1 + \mu _3) + \frac{2 \nu _{tr,\infty } \overline{\delta _3^2}}{\sqrt{\overline{c^2}} + \overline{c}} + \nu _{tr,\infty }^2 \\ &= \nu _{tr,\infty }^2 \mu _3^2 + \frac{2 \nu _{tr,\infty }}{\sqrt{\overline{c^2}} + \overline{c}} \, \overline{\delta _3^2}. \end{aligned}$$

One observes that the expansions above in terms of $\overline{\delta _i^2}$ are singular if, e.g., $\overline{a^2}$ is zero. We therefore distinguish the following two cases.

*Case 1:* $\overline{a^2} \ge \kappa ^2 \wedge \overline{b^2} \ge \kappa ^2 \wedge \overline{c^2} \ge \kappa ^2 \wedge \overline{d^2} \ge \kappa ^2$: The constant $\kappa > 0$ will be chosen according to the calculations in the other Case 2. Here, we have

$$\begin{aligned} \frac{1}{\sqrt{\overline{a^2}} + \overline{a}}, \quad \frac{1}{\sqrt{\overline{b^2}} + \overline{b}}, \quad \frac{1}{\sqrt{\overline{c^2}} + \overline{c}}, \quad \frac{1}{\sqrt{\overline{d^2}} + \overline{d}} \ \le \frac{1}{\kappa } \end{aligned}$$

and

$$\begin{aligned} \frac{\nu _{tr,\infty }'}{\sqrt{\overline{a^2}} + \overline{a}}, \quad \frac{\nu _{tr,\infty }}{\sqrt{\overline{b^2}} + \overline{b}}, \quad \frac{\pi _\infty }{\sqrt{\overline{c^2}} + \overline{c}}, \quad \frac{\nu _\infty }{\sqrt{\overline{d^2}} + \overline{d}} \ \le C(\kappa , \varepsilon _0, n_0, p_0, M, V) \end{aligned}$$

for all $\varepsilon \in (0, \varepsilon _0]$ due to the bounds on $\nu _\infty$ and $\pi _\infty$ from (26). Equation (46) further implies

$$\begin{aligned} (\overline{a} \overline{d} - \overline{c})^2= & {} \left( \nu _\infty \nu _{tr,\infty }'(1 + \mu _1)(1 + \mu _4) - \frac{\nu _\infty (1 + \mu _1)}{\sqrt{\overline{d^2}} + \overline{d}} \, \overline{\delta _4^2} - \frac{\nu _{tr,\infty }' (1 + \mu _4)}{\sqrt{\overline{a^2}} + \overline{a}} \, \overline{\delta _1^2}\right. \\&\left. + \frac{1}{(\sqrt{\overline{a^2}} + \overline{a})(\sqrt{\overline{d^2}} + \overline{d})} \, \overline{\delta _1^2} \, \overline{\delta _4^2} - \nu _{tr,\infty } (1 + \mu _3) + \frac{\overline{\delta _3^2}}{\sqrt{\overline{c^2}} + \overline{c}} \right) ^2 \\\ge & {} \nu _{tr,\infty }^2 \big ((1 + \mu _1)(1 + \mu _4) - (1 + \mu _3) \big )^2 \\&- c_2(\kappa , \varepsilon _0, n_0, p_0, M, M_1, V) \big ( \overline{\delta _1^2} + \overline{\delta _3^2} + \overline{\delta _4^2} \big ) \end{aligned}$$

with an explicit constant $c_2$ thanks to $\nu _\infty \nu _{tr,\infty }' = \nu _{tr,\infty }$ (cf. (25)) and $|\mu _i|, \overline{\delta _i^2} \le c_1(\varepsilon _0, n_0, p_0, M, M_1, V)$. In a similar fashion using $\pi _\infty \nu _{tr,\infty } = \nu _{tr,\infty }'$, one obtains

$$\begin{aligned} (\overline{b} \overline{c} - \overline{d})^2= & {} \left( \pi _\infty \nu _{tr,\infty }(1 + \mu _2)(1 + \mu _3) - \frac{\pi _\infty (1 + \mu _2)}{\sqrt{\overline{c^2}} + \overline{c}} \, \overline{\delta _3^2} - \frac{\nu _{tr,\infty } (1 + \mu _3)}{\sqrt{\overline{b^2}} + \overline{b}} \, \overline{\delta _2^2} \right. \\&\left. + \frac{1}{(\sqrt{\overline{b^2}} + \overline{b})(\sqrt{\overline{c^2}} + \overline{c})} \, \overline{\delta _2^2} \, \overline{\delta _3^2} - \nu _{tr,\infty }' (1 + \mu _4) + \frac{\overline{\delta _4^2}}{\sqrt{\overline{d^2}} + \overline{d}} \right) ^2 \\\ge & {} \nu _{tr,\infty }'^{\, 2} \big ((1 + \mu _2)(1 + \mu _3) - (1 + \mu _4) \big )^2 \\&- c_2(\kappa , \varepsilon _0, n_0, p_0, M, M_1, V) \big ( \overline{\delta _2^2} + \overline{\delta _3^2} + \overline{\delta _4^2} \big ). \end{aligned}$$

In order to finish the proof, it is—according to Step 1—sufficient to show that

$$\begin{aligned}&\nu _\infty ^2 \mu _1^2 + \pi _\infty ^2 \mu _2^2 + \nu _{tr,\infty }^2 \mu _3^2 + \frac{2 \nu _{tr, \infty }}{\sqrt{\overline{c^2}} + \overline{c}} \, \overline{\delta _3^2} \le C_1 \, \bigg ( \Vert \nabla a \Vert ^2 + \Vert \nabla b \Vert ^2 \\&\quad + \frac{1}{2} \big (\overline{a} \, \overline{d} - \overline{c} \big )^2 + \frac{1}{2} \big (\overline{b} \, \overline{c} - \overline{d} \big )^2 - 2\,c_1(n_0, p_0, M_1, V) \left( \overline{\delta _1^2} + \overline{\delta _2^2} + \overline{\delta _3^2} + \overline{\delta _4^2} \right) \bigg ) \\&\quad + C_2 \left( \overline{\delta _1^2} + \overline{\delta _2^2} + \overline{\delta _3^2} + \overline{\delta _4^2} \right) \end{aligned}$$

for appropriate constants $C_1, C_2 > 0$. But due to Step 2 it is sufficient to show that for suitable constants $C_1, C_2 > 0$,

$$\begin{aligned}&\nu _\infty ^2 \mu _1^2 + \pi _\infty ^2 \mu _2^2 + \nu _{tr,\infty }^2 \mu _3^2 + \frac{2 \nu _{tr, \infty }}{\sqrt{\overline{c^2}} + \overline{c}} \, \overline{\delta _3^2} \le C_1 \, \bigg ( \Vert \nabla a \Vert ^2 + \Vert \nabla b \Vert ^2 \\&\quad + \frac{\nu _{tr,\infty }^2}{2} \big ((1 + \mu _1)(1 + \mu _4) - (1 + \mu _3) \big )^2 + \frac{\nu _{tr,\infty }'^{\, 2}}{2} \big ((1 + \mu _2)(1 + \mu _3) - (1 + \mu _4) \big )^2 \\&\quad - c_3(\kappa , \varepsilon _0, n_0, p_0, M, M_1, V) \left( \overline{\delta _1^2} + \overline{\delta _2^2} + \overline{\delta _3^2} + \overline{\delta _4^2} \right) \bigg ) + C_2 \left( \overline{\delta _1^2} + \overline{\delta _2^2} + \overline{\delta _3^2} + \overline{\delta _4^2} \right) . \end{aligned}$$

Collecting all $\overline{\delta _i^2}$-terms on the right hand side, one only has to prove that

$$\begin{aligned}&\nu _\infty ^2 \mu _1^2 + \pi _\infty ^2 \mu _2^2 + \nu _{tr,\infty }^2 \mu _3^2 \le C_1 \bigg ( \Vert \nabla a \Vert ^2 + \Vert \nabla b \Vert ^2 \\&\quad + \nu _{tr,\infty }^2 \big ((1 + \mu _1)(1 + \mu _4) - (1 + \mu _3) \big )^2 + \nu _{tr,\infty }'^{\, 2} \big ((1 + \mu _2)(1 + \mu _3) - (1 + \mu _4) \big )^2 \Bigg ) \\&\quad + \Big (C_2 - C(C_1, \kappa , \varepsilon _0, n_0, p_0, M, M_1, V) \Big ) \left( \overline{\delta _1^2} + \overline{\delta _2^2} + \overline{\delta _3^2} + \overline{\delta _4^2} \right) \end{aligned}$$

or, equivalently,

47 $$\begin{aligned}&\left( \sqrt{\overline{a^2}} - \nu _\infty \right) ^2 + \left( \sqrt{\overline{b^2}} - \pi _\infty \right) ^2 + \left( \sqrt{\overline{c^2}} - \nu _{tr,\infty } \right) ^2 \nonumber \\&\quad \le C_1 \left( \left( \sqrt{\overline{a^2}} \sqrt{\overline{d^2}} - \sqrt{\overline{c^2}} \right) ^2 + \left( \sqrt{\overline{b^2}} \sqrt{\overline{c^2}} - \sqrt{\overline{d^2}} \right) ^2 + \Vert \nabla a \Vert ^2 + \Vert \nabla b \Vert ^2 \right) \nonumber \\&\qquad + \left( C_2 - C(C_1, \kappa , \varepsilon _0, n_0, p_0, M, M_1, V) \right) \left( \overline{\delta _1^2} + \overline{\delta _2^2} + \overline{\delta _3^2} + \overline{\delta _4^2} \right) . \end{aligned}$$

In order to verify (47), we start with the estimate

$$\begin{aligned} \left( \sqrt{\overline{a^2}} - \nu _\infty \right) ^2 \le 2 \left[ \left( \sqrt{\frac{\overline{\mu _n a^2}}{\overline{\mu _n}}} - \nu _\infty \right) ^{\! 2} + \left( \sqrt{\frac{\overline{\mu _n a^2}}{\overline{\mu _n}}} - \sqrt{\overline{a^2}} \right) ^{\! 2} \, \right] \end{aligned}$$

and a corresponding one involving *b*. The last term on the right hand side satisfies

$$\begin{aligned} \left( \sqrt{\frac{\overline{\mu _n a^2}}{\overline{\mu _n}}} - \sqrt{\overline{a^2}} \right) ^{\! 2}= & {} \frac{\left( \frac{\overline{\mu _n a^2}}{\overline{\mu _n}} - \overline{a^2} \right) ^{\! 2}}{\left( \sqrt{\frac{\overline{\mu _n a^2}}{\overline{\mu _n}}} + \sqrt{\overline{a^2}} \right) ^{\! 2}} \le \frac{1}{\kappa ^2} \left( \frac{\overline{\mu _n a^2}}{\overline{\mu _n}} - \overline{a^2} \right) ^{\! 2} \\\le & {} c \int _\Omega \left| \nabla \sqrt{a^2} \right| ^2 dx = c \, \Vert \nabla a \Vert ^2 \end{aligned}$$

due to Lemma 3.5 with a constant $c(\kappa , n_0, p_0, M_1, V) > 0$. Similarly,

$$\begin{aligned} \left( \sqrt{\overline{b^2}} - \pi _\infty \right) ^2 \le c(\kappa , n_0, p_0, M_1, V) \left[ \left( \sqrt{\frac{\overline{\mu _p b^2}}{\overline{\mu _p}}} - \pi _\infty \right) ^{\! 2} + \Vert \nabla b \Vert ^2 \right] . \end{aligned}$$

Proposition 5.3 (with $a^2$, $b^2$, $c^2$, and $d^2$ therein replaced by $\overline{\mu _n a^2}/\overline{\mu _n}$, $\overline{\mu _p b^2}/\overline{\mu _p}$, $\overline{c^2}$, and $\overline{d^2}$) tells us that there exists an explicitly computable constant $C(\varepsilon _0, n_0, p_0, M, M_1, V)>0$ such that

48 $$\begin{aligned}&\left( \sqrt{\frac{\overline{\mu _n a^2}}{\overline{\mu _n}}} - \nu _\infty \right) ^2 + \left( \sqrt{\frac{\overline{\mu _p b^2}}{\overline{\mu _p}}} - \pi _\infty \right) ^2 + \left( \sqrt{\overline{c^2}} - \nu _{tr,\infty } \right) ^2 \nonumber \\&\quad \le C \left( \left( \sqrt{\frac{\overline{\mu _n a^2}}{\overline{\mu _n}}} \sqrt{\overline{d^2}} - \sqrt{\overline{c^2}} \right) ^2 + \left( \sqrt{\frac{\overline{\mu _p b^2}}{\overline{\mu _p}}} \sqrt{\overline{c^2}} - \sqrt{\overline{d^2}} \right) ^2 \right) \end{aligned}$$

for all $\varepsilon \in (0, \varepsilon _0]$. Using an analogue expansion as before, we further deduce with $\overline{d^2} \le 1$,

$$\begin{aligned} \left( \sqrt{\frac{\overline{\mu _n a^2}}{\overline{\mu _n}}} \sqrt{\overline{d^2}} - \sqrt{\overline{c^2}} \right) ^2= & {} \left( \sqrt{\overline{a^2}} \sqrt{\overline{d^2}} - \sqrt{\overline{c^2}} + \left( \sqrt{\frac{\overline{\mu _n a^2}}{\overline{\mu _n}}} - \sqrt{\overline{a^2}} \right) \sqrt{\overline{d^2}} \right) ^2 \\\le & {} c(\kappa , n_0, p_0, M_1, V) \left( \left( \sqrt{\overline{a^2}} \sqrt{\overline{d^2}} - \sqrt{\overline{c^2}} \right) ^2 + \Vert \nabla a \Vert ^2 \right) . \end{aligned}$$

As a corresponding estimate holds true also for the other expression on the right hand side of (48), we have shown that there exists a constant $C_1(\kappa , \varepsilon _0, n_0, p_0, M, M_1, V)>0$ independent of $\varepsilon$ for $\varepsilon \in (0, \varepsilon _0]$ such that

$$\begin{aligned}&\left( \sqrt{\overline{a^2}} - \nu _\infty \right) ^2 + \left( \sqrt{\overline{b^2}} - \pi _\infty \right) ^2 + \left( \sqrt{\overline{c^2}} - \nu _{tr,\infty } \right) ^2 \\&\quad \le C_1 \left( \left( \sqrt{\overline{a^2}} \sqrt{\overline{d^2}} - \sqrt{\overline{c^2}} \right) ^2 + \left( \sqrt{\overline{b^2}} \sqrt{\overline{c^2}} - \sqrt{\overline{d^2}} \right) ^2 + \Vert \nabla a \Vert ^2 + \Vert \nabla b \Vert ^2 \right) . \end{aligned}$$

Choosing $C_2>0$ now sufficiently large, Eq. (47) holds true.

*Case 2*: $\overline{a^2}< \kappa ^2 \vee \overline{b^2}< \kappa ^2 \vee \overline{c^2}< \kappa ^2 \vee \overline{d^2} < \kappa ^2$: In this case, we will not need Proposition 5.3 and we shall directly prove (42) employing only the result of Step 1. In fact, for $\kappa$ chosen sufficiently small, the states considered in Case 2 are necessarily bounded away from the equilibrium and the following arguments show that consequentially the right hand side of (42) is also bounded away from zero, which allows to close the estimate (42). As a result of the hypotheses $\overline{a^2}, \, \overline{b^2} \le C(n_0, p_0, M_1, V)$ and $\overline{c^2}, \, \overline{d^2} \le 1$, we use Young’s inequality to estimate $\overline{a}, \overline{b}, \overline{c}, \overline{d} \le c(n_0, p_0, M_1, V)$ and

$$\begin{aligned} \left( \sqrt{\overline{a^2}} - \nu _\infty \right) ^2 + \left( \sqrt{\overline{b^2}} - \pi _\infty \right) ^2 + \Vert c - \nu _{tr,\infty } \Vert ^2 \le C(\varepsilon _0, n_0, p_0, M, M_1, V) \end{aligned}$$

with $C > 0$ uniformly for $\varepsilon \in (0, \varepsilon _0]$. We stress that the subsequent cases are not necessarily exclusive.

*Case 2.1*: $\overline{c^2} < \kappa ^2$: First, $\overline{c} = \sqrt{\overline{c}^2} \le \sqrt{\overline{c^2}} < \kappa$. This yields

$$\begin{aligned} \overline{d^2}= & {} 1 - \overline{c^2}> 1 - \kappa ^2 \Rightarrow \overline{d}^2 = \overline{d^2} - \overline{\delta _4^2}> 1 - \overline{\delta _4^2} - \kappa ^2\\\Rightarrow & {} \big ( \overline{b} \, \overline{c} - \overline{d} \big )^2 \ge \overline{d}^2 - 2 \overline{b} \, \overline{c} \, \overline{d} > 1 - \overline{\delta _4^2} - \kappa ^2 - 2 \overline{b} \, \overline{d} \, \kappa \\\ge & {} 1 - \overline{\delta _4^2} - \kappa ^2 - C(n_0, p_0, M_1, V) \, \kappa . \end{aligned}$$

For $\kappa > 0$ sufficiently small, we then have $0 < 1 - C(n_0, p_0, M_1, V) \kappa - \kappa ^2 \le ( \overline{b} \, \overline{c} - \overline{d} )^2 + \overline{\delta _4^2}$ and, hence,

$$\begin{aligned}&\left( \sqrt{\overline{a^2}} - \nu _\infty \right) ^2 + \left( \sqrt{\overline{b^2}} - \pi _\infty \right) ^2 + \Vert c - \nu _{tr,\infty } \Vert ^2 \\ &\quad\le C(\varepsilon _0, n_0, p_0, M, M_1, V) \le K( \overline{b} \, \overline{c} - \overline{d} )^2 + K \overline{\delta _4^2} \\ &\quad\le 2 K \Vert bc - d\Vert ^2 + (2 K c_1(n_0, p_0, M_1, V) + K) \left( \overline{\delta _1^2} + \overline{\delta _2^2} + \overline{\delta _3^2} + \overline{\delta _4^2} \right) \end{aligned}$$

by (45) with some $K(\kappa , \varepsilon _0, n_0, p_0, M, M_1, V) > 0$. Let us call the parameter $\kappa$ from above $\kappa _c$.

*Case 2.2*: $\overline{d^2} < \kappa ^2$: Now $\overline{d} = \sqrt{\overline{d}^2} \le \sqrt{\overline{d^2}} < \kappa$ and

$$\begin{aligned} \overline{c^2}= & {} 1 - \overline{d^2}> 1 - \kappa ^2 \Rightarrow \overline{c}^2 = \overline{c^2} - \overline{\delta _3^2}> 1 - \overline{\delta _3^2} - \kappa ^2\\\Rightarrow & {} \big ( \overline{a} \, \overline{d} - \overline{c} \big )^2 \ge \overline{c}^2 - 2 \overline{a} \, \overline{c} \, \overline{d} > 1 - \overline{\delta _3^2} - \kappa ^2 - 2 \overline{a} \, \overline{c} \, \kappa \\\ge & {} 1 - \overline{\delta _3^2} - \kappa ^2 - C(n_0, p_0, M_1, V) \, \kappa . \end{aligned}$$

Again $\kappa > 0$ sufficiently small gives rise to $0 < 1 - C(n_0, p_0, M_1, V) \kappa - \kappa ^2 \le ( \overline{a} \, \overline{d} - \overline{c} )^2 + \overline{\delta _3^2}$ and

$$\begin{aligned}&\Big ( \sqrt{\overline{a^2}} - \nu _\infty \Big )^2 + \Big ( \sqrt{\overline{b^2}} - \pi _\infty \Big )^2 + \Vert c - \nu _{tr,\infty } \Vert ^2 \\ &\quad\le C(\varepsilon _0, n_0, p_0, M, M_1, V) \le K( \overline{a} \, \overline{d} - \overline{c} )^2 + K \overline{\delta _3^2} \\ &\quad\le 2 K \Vert ad - c\Vert ^2 + (2 K c_1(n_0, p_0, M_1, V) + K) \left( \overline{\delta _1^2} + \overline{\delta _2^2} + \overline{\delta _3^2} + \overline{\delta _4^2} \right) \end{aligned}$$

for some constant $K(\kappa , \varepsilon _0, n_0, p_0, M, M_1, V) > 0$ using (44). This $\kappa > 0$ shall be denoted by $\kappa _d$.

*Case 2.3*: $\overline{a^2} < \kappa ^2$: We first notice that $\overline{a} < \kappa$ and $2 \, \overline{c} \, \overline{d} \le \overline{c}^2 + \overline{d}^2 \le \overline{c^2} + \overline{d^2} = 1$. Now, we choose $\kappa _a := \kappa > 0$ sufficiently small such that $2 \kappa < \kappa _c^2$. Then, if $\overline{c^2} < 2 \kappa$, we have $\overline{c^2} < \kappa _c^2$, and the estimate

$$\begin{aligned}&\left( \sqrt{\overline{a^2}} - \nu _\infty \right) ^2 + \left( \sqrt{\overline{b^2}} - \pi _\infty \right) ^2 + \Vert c - \nu _{tr,\infty } \Vert ^2 \\&\quad \le 2 K \Vert bc - d\Vert ^2 + (2 K c_1(n_0, p_0, M_1, V) + K) \left( \overline{\delta _1^2} + \overline{\delta _2^2} + \overline{\delta _3^2} + \overline{\delta _4^2} \right) \end{aligned}$$

with $K(\kappa , \varepsilon _0, n_0, p_0, M, M_1, V) > 0$ immediately follows from Case 2.1. And if $\overline{c^2} \ge 2 \kappa$, then

$$\begin{aligned} \overline{c}^2 = \overline{c^2} - \overline{\delta _3^2} \ge 2 \kappa - \overline{\delta _3^2} \Rightarrow \big ( \overline{a} \, \overline{d} - \overline{c} \big )^2 \ge \overline{c}^2 - 2 \, \overline{a} \, \overline{c} \, \overline{d} \ge 2 \kappa - \overline{\delta _3^2} - \kappa = \kappa - \overline{\delta _3^2}. \end{aligned}$$

Consequently, $$0 < \kappa \le ( \overline{a} \, \overline{d} - \overline{c} )^2 + \overline{\delta _3^2}$$ and

$$\begin{aligned}&\left( \sqrt{\overline{a^2}} - \nu _\infty \right) ^2 + \left( \sqrt{\overline{b^2}} - \pi _\infty \right) ^2 + \Vert c - \nu _{tr,\infty } \Vert ^2 \\ &\quad\le C(\varepsilon _0, n_0, p_0, M, M_1, V) \le K( \overline{a} \, \overline{d} - \overline{c} )^2 + K \overline{\delta _3^2} \\ &\quad\le 2 K \Vert ad - c\Vert ^2 + (2 K c_1(n_0, p_0, M_1, V) + K) \left( \overline{\delta _1^2} + \overline{\delta _2^2} + \overline{\delta _3^2} + \overline{\delta _4^2} \right) \end{aligned}$$

due to (44) with a constant $K(\kappa , \varepsilon _0, n_0, p_0, M, M_1, V) > 0$.

*Case 2.4*: $\overline{b^2} < \kappa ^2$: Again $\overline{b} < \kappa$ and $2 \, \overline{c} \, \overline{d} \le \overline{c}^2 + \overline{d}^2 \le \overline{c^2} + \overline{d^2} = 1$. Here, we choose $\kappa _b := \kappa > 0$ sufficiently small such that $2\kappa < \kappa _d^2$. If $\overline{d^2} < 2\kappa$, we have $\overline{d^2} < \kappa _d^2$, and due to Case 2.2 there exists some $K(\kappa , \varepsilon _0, n_0, p_0, M, M_1, V) > 0$ such that

$$\begin{aligned}&\left( \sqrt{\overline{a^2}} - \nu _\infty \right) ^2 + \left( \sqrt{\overline{b^2}} - \pi _\infty \right) ^2 + \Vert c - \nu _{tr,\infty } \Vert ^2 \\ &\quad\le 2 K \Vert ad - c\Vert ^2 + (2 K c_1(n_0, p_0, M_1, V) + K) \Bigl ( \overline{\delta _1^2} + \overline{\delta _2^2} + \overline{\delta _3^2} + \overline{\delta _4^2} \Bigr ). \end{aligned}$$

If $$\overline{d^2} \ge 2 \kappa$$, then

$$\begin{aligned} \overline{d}^2 = \overline{d^2} - \overline{\delta _4^2} \ge 2 \kappa - \overline{\delta _4^2} \Rightarrow \big ( \overline{b} \, \overline{c} - \overline{d} \big )^2 \ge \overline{d}^2 - 2 \, \overline{b} \, \overline{c} \, \overline{d} \ge 2 \kappa - \overline{\delta _4^2} - \kappa = \kappa - \overline{\delta _4^2}. \end{aligned}$$

This implies $0 < \kappa \le ( \overline{b} \, \overline{c} - \overline{d} )^2 + \overline{\delta _4^2}$ and

$$\begin{aligned}&\left( \sqrt{\overline{a^2}} - \nu _\infty \right) ^2 + \left( \sqrt{\overline{b^2}} - \pi _\infty \right) ^2 + \Vert c - \nu _{tr,\infty } \Vert ^2 \\ &\quad\le C(\varepsilon _0, n_0, p_0, M, M_1, V) \le K( \overline{b} \, \overline{c} - \overline{d} )^2 + K \overline{\delta _4^2} \\ &\quad\le 2 K \Vert bc - d\Vert ^2 + (2 K c_1(n_0, p_0, M_1, V) + K) \left( \overline{\delta _1^2} + \overline{\delta _2^2} + \overline{\delta _3^2} + \overline{\delta _4^2} \right) \end{aligned}$$

with $$K(\kappa , \varepsilon _0, n_0, p_0, M, M_1, V) > 0$$ employing (45).

All arguments within Step 2 remain valid, if we finally set $\kappa := \min (\kappa _a, \kappa _b, \kappa _c, \kappa _d)$. We also observe that the constants $K > 0$ above are independent of $\varepsilon \in (0, \varepsilon _0]$. And since $\kappa$ only depends on $n_0$, $p_0$, $M_1$ and *V*, we may skip the explicit dependence of $C_2$ on $\kappa$ at the end of Case 1. This finishes the proof. $\square$

We already pointed out that $\Vert ad - c\Vert ^2$ and $\Vert bc - d\Vert ^2$ can be controlled by the reaction terms of the entropy production, if we replace *a*, *b*, *c*, *d* by $\sqrt{n/(n_0 \mu _n)}$, $\sqrt{p/(p_0 \mu _p)}$, $\sqrt{n_{tr}}$ and $\sqrt{n_{tr}'}$ (see the proof of Theorem 1.5 in Sect. 6 for details). In this proof, also $\Vert \nabla a\Vert ^2$, $\Vert \nabla b\Vert ^2$, $\Vert a - \overline{a}\Vert ^2$ and $\Vert b - \overline{b}\Vert ^2$ may be bounded by the entropy production. However, $\Vert c - \overline{c}\Vert ^2$ and $\Vert d - \overline{d}\Vert ^2$ may not be estimated with the help of Poincaré’s inequality since this would yield terms involving $\nabla n_{tr}$, which do not appear in the entropy production.

Instead, we are able to derive the following estimates for $\Vert c - \overline{c}\Vert ^2$ and $\Vert d - \overline{d}\Vert ^2$, which describe an indirect diffusion transfer from *c* to *b* and from *d* to *a*, respectively: Even if *c* and *d* are lacking an explicit diffusion term in the dynamical equations, they do experience indirect diffusive effects thanks to the reversible reaction dynamics and the diffusivity of *a* and *b*. This is the interpretation of the following functional inequalities.

### Proposition 5.6

(Indirect diffusion transfer) Let $a,b,c,d:\Omega \rightarrow {\mathbb {R}}$ be non-negative functions such that

$$\begin{aligned} c^2 + d^2 = 1 \end{aligned}$$

holds true a.e. in $\Omega$. Then,

$$\begin{aligned} \Vert c - \overline{c} \Vert ^2 \le 4 \big ( \Vert b c - d \Vert ^2 + \Vert b - \overline{b} \Vert ^2 \big ) \quad \text{ and } \quad \Vert d - \overline{d} \Vert ^2 \le 4 \big ( \Vert a d - c \Vert ^2 + \Vert a - \overline{a} \Vert ^2 \big ). \end{aligned}$$

### Proof

We only verify the second inequality; the first one can be checked along the same lines. First, we notice that

49 $$\begin{aligned} \Vert \overline{a} d - c \Vert = \Vert a d - c + (\overline{a} - a) d \Vert \le \Vert a d - c \Vert + \Vert a - \overline{a} \Vert \end{aligned}$$

because of the bound $0 \le d \le 1$. Besides, we deduce

$$\begin{aligned} \Vert \overline{a}^2 d^2 - c^2 \Vert = \Vert (\overline{a} d + c)(\overline{a} d - c) \Vert \le (1 + \overline{a}) \Vert \overline{a} d - c \Vert \end{aligned}$$

employing $0 \le c,d \le 1$. For the subsequent estimates, we need two auxiliary inequalities: For every function $f: \Omega \rightarrow {\mathbb {R}}$ and all $\lambda \in {\mathbb {R}}$, we have

50 $$\begin{aligned} \Vert f - \overline{f} \Vert ^2&= \int _\Omega (f - \lambda + \lambda - \overline{f})^2 dx\nonumber \\&= \int _\Omega \Big ( (f - \lambda )^2 - 2 (f - \lambda ) (\overline{f} - \lambda ) + (\overline{f} - \lambda )^2 \Big ) dx \nonumber \\&= \int _\Omega (f - \lambda )^2 dx - (\overline{f} - \lambda )^2 \le \Vert f - \lambda \Vert ^2. \end{aligned}$$

And for all $x \ge 0$, one has

$$\begin{aligned} \frac{1+x}{\sqrt{1 + x^2}} = \frac{\sqrt{1 + 2x + x^2}}{\sqrt{1 + x^2}} \le \frac{\sqrt{2 (1 + x^2)}}{\sqrt{1 + x^2}} = \sqrt{2}. \end{aligned}$$

Since $c^2 + d^2 = 1$, we obtain

$$\begin{aligned} \Vert \overline{a}^2 d^2 - c^2 \Vert= & {} \Vert \overline{a}^2 d^2 + d^2 - 1 \Vert = \Vert (1 + \overline{a}^2) d^2 - 1 \Vert \\= & {} \big \Vert \big ( \sqrt{1 + \overline{a}^2} \, d + 1 \big ) \big ( \sqrt{1 + \overline{a}^2} \, d - 1 \big ) \big \Vert \\\ge & {} \big \Vert \sqrt{1 + \overline{a}^2} \, d - 1 \big \Vert = \sqrt{1 + \overline{a}^2} \, \bigg \Vert d - \frac{1}{\sqrt{1 + \overline{a}^2}} \bigg \Vert \ge \sqrt{1 + \overline{a}^2} \Vert d - \overline{d} \Vert . \end{aligned}$$

where we applied (50) in the last step. Consequently,

$$\begin{aligned} \Vert d - \overline{d} \Vert \le \frac{1}{\sqrt{1 + \overline{a}^2}} \Vert \overline{a}^2 d^2 - c^2 \Vert \le \frac{1 + \overline{a}}{\sqrt{1 + \overline{a}^2}} \Vert \overline{a} d - c \Vert \le \sqrt{2} \, \Vert \overline{a} d - c \Vert \end{aligned}$$

and

$$\begin{aligned} \Vert d - \overline{d} \Vert ^2 \le 2 \Vert \overline{a} d - c \Vert ^2 \le 4 \left( \Vert a d - c \Vert ^2 + \Vert a - \overline{a} \Vert ^2 \right) \end{aligned}$$

using (49). $$\square$$

## EEP-inequality and convergence to the equilibrium

We are now prepared to prove Theorem 1.5.

### Proof of Theorem 1.5

Let $(n,p,n_{tr}) \in L^1(\Omega )^3$ be non-negative functions satisfying $n_{tr} \le 1$, the conservation law $\overline{n} - \overline{p} + \varepsilon \overline{n_{tr}} = M$ and the $L^1$-bound $\overline{n}, \overline{p} \le M_1$. Keeping in mind that $\nu _\infty = \sqrt{n_*/n_0}$ and $\pi _\infty = \sqrt{p_*/p_0}$ (cf. Notation 5.1), Proposition 4.4 guarantees that there exists a positive constant $C_1(\gamma , \Gamma , M_1) > 0$ such that

51 $$\begin{aligned}&E(n,p,n_{tr}) - E(n_\infty , p_\infty , n_{tr,\infty }) \le C_1 \, \left( \int _\Omega \left( \frac{|J_n|^2}{n} + \frac{|J_p|^2}{p} \right) dx \right. \nonumber \\&\quad \left. + n_0 \left( \sqrt{\overline{\frac{n}{n_0 \mu _n}}} - \nu _\infty \right) ^2 + p_0 \left( \sqrt{\overline{\frac{p}{p_0 \mu _p}}} - \pi _\infty \right) ^2 + \varepsilon \int _\Omega \left( \sqrt{n_{tr}} - \sqrt{n_{tr,\infty }} \right) ^2 \, dx \right) . \end{aligned}$$

Next, we have to bound the second line of (51) in terms of the entropy production. To this end, we apply Proposition 5.5 with the choices $a := \sqrt{n/(n_0 \mu _n)}$, $b := \sqrt{p/(p_0 \mu _p)}$, $c := \sqrt{n_{tr}}$ and $d := \sqrt{n_{tr}'}$ (as always $n_{tr}' = 1 - n_{tr}$). The hypotheses of this proposition are fulfilled as a consequence of the conservation law $\overline{n} - \overline{p} + \varepsilon \overline{n_{tr}} = M$ and the $L^1$-bound $\overline{n}, \overline{p} \le M_1$. As a result, we obtain

$$\begin{aligned}&\left( \sqrt{\overline{\frac{n}{n_0 \mu _n}}} - \nu _\infty \right) ^2 + \left( \sqrt{\overline{\frac{p}{p_0 \mu _p}}} - \pi _\infty \right) ^2 + \Vert \sqrt{n_{tr}} - \sqrt{n_{tr,\infty }} \Vert ^2 \\&\quad \le C_2 \Bigg ( \left\| \sqrt{\frac{n n_{tr}'}{n_0 \mu _n}} - \sqrt{n_{tr}} \right\| ^2 + \left\| \sqrt{\frac{p n_{tr}}{p_0 \mu _p}} - \sqrt{n_{tr}'} \right\| ^2 \\&\qquad + \left\| \nabla \sqrt{\frac{n}{n_0 \mu _n}} \right\| ^2 + \left\| \nabla \sqrt{\frac{p}{p_0 \mu _p}} \right\| ^2 \\&\qquad + \left\| \sqrt{\frac{n}{n_0 \mu _n}} - \overline{\sqrt{\frac{n}{n_0 \mu _n}}} \right\| ^2 + \left\| \sqrt{\frac{p}{p_0 \mu _p}} - \overline{\sqrt{\frac{p}{p_0 \mu _p}}} \right\| ^2 \\&\qquad + \big \Vert \sqrt{n_{tr}} - \overline{\sqrt{n_{tr}}} \big \Vert ^2 + \big \Vert \sqrt{n_{tr}'} - \overline{\sqrt{n_{tr}'}} \big \Vert ^2 \Bigg ) \end{aligned}$$

for all $\varepsilon \in (0, \varepsilon _0]$ with a constant $C_2(\varepsilon _0, n_0, p_0, M, M_1, V) > 0$. Thanks to Poincaré’s inequality, we are able to bound the second and third line on the right hand side from above:

$$\begin{aligned}&\left\| \sqrt{\frac{n}{n_0 \mu _n}} - \overline{\sqrt{\frac{n}{n_0 \mu _n}}} \right\| ^2 \le C_P \left\| \nabla \sqrt{\frac{n}{n_0 \mu _n}} \right\| ^2 \\&\quad = C_P \int _{\Omega } \left| \frac{1}{2} \sqrt{\frac{\mu _n}{n_0 n}} \nabla \Big ( \frac{n}{\mu _n} \Big ) \right| ^2 dx \le \frac{C_P}{4 n_0 \inf _\Omega \mu _n} \int _{\Omega } \frac{|J_n|^2}{n} \, dx \end{aligned}$$

and

$$\begin{aligned} \left\| \sqrt{\frac{p}{p_0 \mu _p}} - \overline{\sqrt{\frac{p}{p_0 \mu _p}}} \right\| ^2 \le C_P \left\| \nabla \sqrt{\frac{p}{p_0 \mu _p}} \right\| ^2 \le \frac{C_P}{4 p_0 \inf _\Omega \mu _p} \int _{\Omega } \frac{|J_p|^2}{p} \, dx. \end{aligned}$$

Moreover, the elementary inequality $(\sqrt{x} - 1)^2 \le (x-1) \ln (x)$ for $x>0$ gives rise to

$$\begin{aligned} \left\| \sqrt{\frac{n n_{tr}'}{n_0 \mu _n}} - \sqrt{n_{tr}} \right\| ^2= & {} \int _\Omega n_{tr} \left( \sqrt{\frac{n n_{tr}'}{n_0 \mu _n n_{tr}}} - 1 \right) ^2 dx \\\le & {} \int _\Omega n_{tr} \left( \frac{n n_{tr}'}{n_0 \mu _n n_{tr}} - 1 \right) \ln \left( \frac{n n_{tr}'}{n_0 \mu _n n_{tr}} \right) dx \\= & {} \int _\Omega \left( \frac{n (1-n_{tr})}{n_0 \mu _n} - n_{tr} \right) \ln \left( \frac{n (1-n_{tr})}{n_0 \mu _n n_{tr}} \right) dx \\= & {} \tau _n \int _\Omega (-R_n) \ln \left( \frac{n (1-n_{tr})}{n_0 \mu _n n_{tr}} \right) dx \end{aligned}$$

and similarly

$$\begin{aligned} \left\| \sqrt{\frac{p n_{tr}}{p_0 \mu _p}} - \sqrt{n_{tr}'} \right\| ^2 \le \tau _p \int _\Omega (-R_p) \ln \left( \frac{p n_{tr}}{p_0 \mu _p (1-n_{tr})} \right) dx. \end{aligned}$$

Proposition 5.6 further implies that

$$\begin{aligned}&\big \Vert \sqrt{n_{tr}} - \overline{\sqrt{n_{tr}}} \big \Vert ^2 + \big \Vert \sqrt{n_{tr}'} - \overline{\sqrt{n_{tr}'}} \big \Vert ^2 \\&\quad \le 4 \left( \left\| \sqrt{\frac{n}{n_0 \mu _n}} - \overline{\sqrt{\frac{n}{n_0 \mu _n}}} \right\| ^2 + \left\| \sqrt{\frac{p}{p_0 \mu _p}} - \overline{\sqrt{\frac{p}{p_0 \mu _p}}} \right\| ^2 \right. \\&\qquad \left. + \left\| \sqrt{\frac{n n_{tr}'}{n_0 \mu _n}} - \sqrt{n_{tr}} \right\| ^2 + \left\| \sqrt{\frac{p n_{tr}}{p_0 \mu _p}} - \sqrt{n_{tr}'} \right\| ^2 \right) . \end{aligned}$$

Combining the above estimates, we arrive at

$$\begin{aligned}&\left( \sqrt{\overline{\frac{n}{n_0 \mu _n}}} - \nu _\infty \right) ^2 + \left( \sqrt{\overline{\frac{p}{p_0 \mu _p}}} - \pi _\infty \right) ^2 + \Vert \sqrt{n_{tr}} - \sqrt{n_{tr,\infty }} \Vert ^2 \\&\quad \le C_3 \int _{\Omega } \left( \frac{|J_n|^2}{n} + \frac{|J_p|^2}{p} - R_n \ln \left( \frac{n(1-n_{tr})}{n_0 \mu _n n_{tr}} \right) - R_p \ln \left( \frac{p n_{tr}}{p_0 \mu _p (1-n_{tr})} \right) \right) \end{aligned}$$

with a constant $C_3(\varepsilon _0, \tau _n, \tau _p, n_0, p_0, M, M_1, V) > 0$ uniformly for $\varepsilon \in (0, \varepsilon _0]$. With respect to (51), we now find

$$\begin{aligned} n_0&\left( \sqrt{\overline{\frac{n}{n_0 \mu _n}}} - \nu _\infty \right) ^2 + p_0 \left( \sqrt{\overline{\frac{p}{p_0 \mu _p}}} - \pi _\infty \right) ^2 + \varepsilon \int _\Omega \big ( \sqrt{n_{tr}} - \sqrt{n_{tr,\infty }} \big )^2 \, dx \\&\quad \le \max \{n_0, p_0, \varepsilon _0\} \left( \left( \sqrt{\overline{\frac{n}{n_0 \mu _n}}} - \nu _\infty \right) ^2 \right. \\&\qquad \left. + \left( \sqrt{\overline{\frac{p}{p_0 \mu _p}}} - \pi _\infty \right) ^2 + \Vert \sqrt{n_{tr}} - \sqrt{n_{tr,\infty }} \Vert ^2 \right) \\&\quad \le C_3 \max \{n_0, p_0, \varepsilon _0\} \int _{\Omega } \left( \frac{|J_n|^2}{n} + \frac{|J_p|^2}{p} - R_n \ln \left( \frac{n(1-n_{tr})}{n_0 \mu _n n_{tr}} \right) \right. \\&\qquad \left. - R_p \ln \left( \frac{p n_{tr}}{p_0 \mu _p (1-n_{tr})} \right) \right) . \end{aligned}$$

And since the constant $C_1$ in (51) only depends on $\varepsilon _0$, $n_0$, $p_0$, *M*, $M_1$ and *V* (via the constants $\gamma$ and $\Gamma$), we have finally proven that

$$\begin{aligned}&E(n,p,n_{tr}) - E(n_\infty , p_\infty , n_{tr,\infty }) \\&\quad \le C_4 \int _{\Omega } \left( \frac{|J_n|^2}{n} + \frac{|J_p|^2}{p} - R_n \ln \left( \frac{n(1-n_{tr})}{n_0 \mu _n n_{tr}} \right) - R_p \ln \left( \frac{p n_{tr}}{p_0 \mu _p (1-n_{tr})} \right) \right) dx \end{aligned}$$

for a constant $C_4(\varepsilon _0, \tau _n, \tau _p, n_0, p_0, M, M_1, V) > 0$ independent of $\varepsilon \in (0, \varepsilon _0]$. $\square$

Theorem 1.5 provides an upper bound for the relative entropy in terms of the entropy production. This already implies exponential convergence of the relative entropy. The subsequent proposition now yields a lower bound for the relative entropy involving the $L^1$-distance of the solution to the equilibrium. This will allow us to establish exponential convergence in $L^1$.

### Proposition 6.1

(Csiszár–Kullback–Pinsker inequality) Let $\varepsilon _0$, $n_0$, $p_0$, *M*, $M_1$ and *V* be positive constants. Then, there exists an explicit constant $C_{\mathrm {CKP}} > 0$ such that for all $\varepsilon \in (0, \varepsilon _0]$, the equilibrium $(n_\infty , p_\infty , n_{tr,\infty }) \in X$ from Theorem 2.1 and all non-negative functions $(n,p,n_{tr}) \in L^1(\Omega )^3$ satisfying $n_{tr} \le 1$, the conservation law

$$\begin{aligned} \overline{n} - \overline{p} + \varepsilon \overline{n_{tr}} = M \end{aligned}$$

and the $L^1$-bound

$$\begin{aligned} \overline{n}, \overline{p} \le M_1, \end{aligned}$$

the following Csiszár–Kullback–Pinsker-type inequality holds true:

$$\begin{aligned} &E(n,p,n_{tr}) - E(n_\infty , p_\infty , n_{tr,\infty }) \\ &\quad\ge C_{\mathrm {CKP}} \big ( \Vert n - n_\infty \Vert _{L^1(\Omega )}^2 + \Vert p - p_\infty \Vert _{L^1(\Omega )}^2 + \varepsilon \Vert n_{tr} - n_{tr,\infty } \Vert _{L^2(\Omega )}^2 \big ). \end{aligned}$$

### Proof

Due to Lemma 3.2, we know that the relative entropy reads

$$\begin{aligned}&E(n,p,n_{tr}) - E(n_\infty , p_\infty , n_{tr,\infty }) \\&\quad = \int _{\Omega } \left( n \ln \frac{n}{n_\infty } - (n-n_\infty ) + p \ln \frac{p}{p_\infty } - (p-p_\infty ) \right. \\&\qquad \left. + \varepsilon \int _{n_{tr,\infty }}^{n_{tr}} \left( \ln \left( \frac{s}{1-s} \right) - \ln \left( \frac{n_{tr,\infty }}{1-n_{tr,\infty }} \right) \right) ds \right) dx. \end{aligned}$$

Similar to Proposition 4.4, we employ the mean-value theorem and observe that

$$\begin{aligned} \frac{d}{ds} \ln \left( \frac{s}{1-s} \right) = \frac{1}{s(1-s)} \ge 4 \end{aligned}$$

for all $s \in (0,1)$. Thus, there exists some $\sigma (s)$ between $n_{tr,\infty }$ and *s* such that

$$\begin{aligned}&\varepsilon \int _\Omega \int _{n_{tr,\infty }}^{n_{tr}} \left( \ln \left( \frac{s}{1-s} \right) - \ln \left( \frac{n_{tr,\infty }}{1 - n_{tr,\infty }} \right) \right) ds \, dx \\&\quad = \varepsilon \int _\Omega \int _{n_{tr,\infty }}^{n_{tr}} \frac{1}{\sigma (s)(1 - \sigma (s))} (s - n_{tr,\infty }) \, ds \, dx \\&\quad \ge 4 \varepsilon \int _\Omega \int _{n_{tr,\infty }}^{n_{tr}} (s - n_{tr,\infty }) \, ds \, dx = 2 \varepsilon \int _\Omega (n_{tr} - n_{tr,\infty })^2 \, dx \end{aligned}$$

Moreover, we utilise the Csiszár–Kullback–Pinsker-inequality from Lemma 3.3 to estimate

$$\begin{aligned} \int _\Omega \left( n \ln \left( \frac{n}{n_\infty } \right) - (n - n_\infty ) \right) dx \ge \frac{3}{2 \overline{n} + 4 \overline{n_\infty }} \Vert n - n_\infty \Vert _{L^1(\Omega )}^2 \ge c \Vert n - n_\infty \Vert _{L^1(\Omega )}^2 \end{aligned}$$

where $c(\varepsilon _0, n_0, p_0, M, M_1, V) > 0$ is a positive constant independent of $\varepsilon \in (0, \varepsilon _0]$. As a corresponding estimate holds true also for *p*, we have verified that

$$\begin{aligned} &E(n,p,n_{tr}) - E(n_\infty , p_\infty , n_{tr,\infty }) \\ &\quad\ge C \big ( \Vert n - n_\infty \Vert _{L^1(\Omega )}^2 + \Vert p - p_\infty \Vert _{L^1(\Omega )}^2 + \varepsilon \Vert n_{tr} - n_{tr,\infty } \Vert _{L^2(\Omega )}^2 \big ) \end{aligned}$$

for some $C(\varepsilon _0, n_0, p_0, M, M_1, V) > 0$ uniformly for $\varepsilon \in (0, \varepsilon _0]$. $\square$

Now, we are able to prove exponential convergence in relative entropy and $L^1$.

### Proof of Theorem 1.3

We first prove exponential convergence of the relative entropy

$$\begin{aligned} \psi (t) := E(n,p,n_{tr})(t) - E(n_\infty , p_\infty , n_{tr,\infty }) \end{aligned}$$

using a Gronwall argument as stated in [25]. To this end, we choose $0< t_0 \le t_1 \le t < T$ and rewrite the entropy production law as

52 $$\begin{aligned} \psi (t_1) - \psi (t) = \int _{t_1}^{t} P(n, p, n_{tr})(s) \, ds \ge K \int _{t_1}^{t} \psi (s) \, ds \end{aligned}$$

where we applied Theorem 1.5 with $K := C_{\mathrm {EEP}}^{-1}$ in the second step. Furthermore, we set

$$\begin{aligned} \Psi (t_1) := \int _{t_1}^{t} \psi (s) \, ds = - \int _{t}^{t_1} \psi (s) \, ds \end{aligned}$$

and obtain from (52) the estimate $K \Psi (t_1) \le \psi (t_1) - \psi (t)$ which yields

$$\begin{aligned} \frac{d}{dt_1} \Big ( \Psi (t_1) e^{K t_1} \Big ) = - \psi (t_1) e^{K t_1} + K \Psi (t_1) e^{K t_1} \le - \psi (t) e^{K t_1}. \end{aligned}$$

Integrating this inequality from $t_1 = t_0$ to $t_1 = t$ and observing that $\Psi (t) = 0$ gives rise to

$$\begin{aligned} -\Psi (t_0) e^{K t_0} \le - \frac{\psi (t)}{K} \big ( e^{K t} - e^{K t_0} \big ). \end{aligned}$$

As a consequence of (52) with $t_1 = t_0$, one has $- \Psi (t_0) \ge (\psi (t) - \psi (t_0)) / K$ and, hence,

$$\begin{aligned} - \psi (t_0) e^{K t_0} \le - \psi (t) e^{K t}. \end{aligned}$$

But this is equivalent to

53 $$\begin{aligned} E(n,p,n_{tr})(t) - E(n_\infty , p_\infty , n_{tr,\infty }) \le (E(n,p,n_{tr})(t_0) - E_\infty ) e^{-K (t-t_0)}, \end{aligned}$$

for all $t\ge t_0>0$. In order to conclude that

$$\begin{aligned} E(n,p,n_{tr})(t) - E(n_\infty , p_\infty , n_{tr,\infty }) \le (E_I - E_\infty ) e^{-K t}, \end{aligned}$$

for all $t \ge 0$, we observe that the rate *K* is independent of $t_0$ and that the entropy $E(n,p,n_{tr})(t_0)$ extends in (53) continuously to $t_0 \rightarrow 0$ since $n,p \in C([0,T);L^2(\Omega ))$ for all $T>0$ by Theorem 1.1. This results in the announced exponential decay of the relative entropy, while the exponential convergence in $L^1$ follows from Proposition 6.1. $\square$

### Proof of Corollary 1.8

We first prove that the linearly growing $L^{\infty }$-bounds together with parabolic regularity for system (1) and assumption (4) entail polynomially growing $W^{1,q}$-bounds, $q \in (1,\infty )$, for *n* and *p*. To this end, we consider

$$\begin{aligned} \partial _t n = \nabla \cdot J_n + \frac{1}{\tau _n} \left( n_{tr} - \frac{n}{n_0 e^{-V_n}} \bigl (1-n_{tr}\bigr ) \right) ,\quad J_n=e^{-V_n} \nabla \big (n\, e^{V_n} \bigr ), \end{aligned}$$

and introduce the variable $w=n \, e^{V_n}$. We observe that $\nabla \cdot J_n = \nabla \cdot \big (e^{-V_n} \nabla w \big )=e^{-V_n}\left( \Delta w - \nabla V_n \cdot \nabla w\right)$ and thus,

54 $$\begin{aligned} \partial _t w = \Delta w - \nabla V_n \cdot \nabla w + \frac{e^{V_n}}{\tau _n} \left( n_{tr} - \frac{1-n_{tr}}{n_0} w \right) . \end{aligned}$$

Under the assumptions of Corollary 1.8, this equation is of the form

$$\begin{aligned} \partial _t w - \Delta w = f_1 + f_2 w + f_3 \cdot \nabla w \end{aligned}$$

where $f_i \in C([0, \infty ), L^\infty (\Omega ))$ for $i \in \{1,2\}$, $f_3 \in C([0, \infty ), L^\infty (\Omega )^m)$ and ${\hat{n}} \cdot \nabla w = 0$ on $\partial \Omega$. Testing this equation with $-(q-1) |\nabla w|^{q-2} \Delta w$ yields

$$\begin{aligned}&\frac{1}{q} \frac{d}{dt} \int _\Omega |\nabla w|^q \, dx = \int _\Omega |\nabla w|^{q-2} \nabla w \cdot \nabla \partial _t w \, dx \\&\quad = - \int _\Omega \bigg ( (q-2) |\nabla w|^{q-4} \nabla w \Delta w \cdot \nabla w + |\nabla w|^{q-2} \Delta w \bigg ) \partial _t w \, dx\\&\quad = - \int _\Omega (q-1) |\nabla w|^{q-2} \Delta w \, \partial _t w \, dx \\&\quad = - \int _\Omega (q-1) |\nabla w|^{q-2} |\Delta w|^2 dx \\&\qquad - \int _\Omega (q-1) |\nabla w|^{q-2} \Delta w \big ( f_1 + f_2 w + f_3 \cdot \nabla w \big ) dx. \end{aligned}$$

Using the inequalities $|ab| \le (a^2 + b^2)/2$ and $(a+b+c)^2 \le 3(a^2 + b^2 + c^2)$ for $a,b,c \in {\mathbb {R}}$, we find

$$\begin{aligned}&\frac{1}{q} \frac{d}{dt} \int _\Omega |\nabla w|^q \, dx \le -\frac{1}{2} \int _\Omega (q-1) |\nabla w|^{q-2} |\Delta w|^2 \, dx \\&\quad + \frac{3}{2} \int _\Omega (q-1) |\nabla w|^{q-2} \big ( f_1^2 + f_2^2 w^2 + f_3^2 |\nabla w|^2 \big ) dx. \end{aligned}$$

Together with $C > 0$ satisfying $|f_i(t,x)^2| \le C$ for all $i \in \{1,2,3\}$, $t \ge 0$ and a.e. $x \in \Omega$, we derive

$$\begin{aligned}&\frac{1}{q} \frac{d}{dt} \int _\Omega |\nabla w|^q \, dx \le -\frac{1}{2} \int _\Omega (q-1) |\nabla w|^{q-2} |\Delta w|^2 \, dx \\&\quad + \frac{3C}{2} \int _\Omega (q-1) |\nabla w|^{q-2} \big ( 1 + w^2 + |\nabla w|^2 \big ) dx. \end{aligned}$$

An integration by parts and Young’s inequality with $C_1(C, q) > 0$ give rise to

$$\begin{aligned}&\int _\Omega (q-1) |\nabla w|^{q-2} \nabla w \cdot \nabla w \, dx \\&\quad = - \int _{\Omega } \bigg ( (q-1)(q-2) |\nabla w|^{q-4} \nabla w \Delta w \cdot \nabla w + (q-1) |\nabla w|^{q-2} \Delta w \bigg ) w \, dx \\&\quad = - \int _\Omega (q-1)^2 |\nabla w|^{q-2} \Delta w \, w \, dx \\ &\quad\le \frac{1}{3C} \int _\Omega (q-1)^1 |\nabla w|^{q-2} |\Delta w|^2 \, dx + C_1 \int _\Omega |\nabla w|^{q-2} w^2 \, dx. \end{aligned}$$

Hence, there exists a constant $C_2(C, q) > 0$ such that

$$\begin{aligned} \frac{d}{dt} \int _\Omega |\nabla w|^q \, dx \le C_2 \int _\Omega |\nabla w|^{q-2} (1 + w^2) \, dx \le (A + B \, t^2) \int _\Omega |\nabla w|^{q-2} \, dx, \end{aligned}$$

where $A, B > 0$ result from the linearly growing $L^\infty$-bounds from (11). For any fixed $t_0 > 0$ and all $t \ge t_0$, we now have

$$\begin{aligned} \Vert \nabla w(t) \Vert _{L^q(\Omega )}^q \le \Vert \nabla w(t_0) \Vert _{L^q(\Omega )}^q + \int _{t_0}^t (A + B \, s^2) \Vert \nabla w(s) \Vert _{L^q(\Omega )}^{q-2} \, ds. \end{aligned}$$

A Gronwall lemma (see e.g. [2]) now proves the desired polynomial growth of $\Vert \nabla w \Vert _{L^q(\Omega )}$ and $\Vert \nabla n \Vert _{L^q(\Omega )}$:

55 $$\begin{aligned} \Vert \nabla w(t) \Vert _{L^q(\Omega )} \le \bigg ( \Vert \nabla w(t_0) \Vert _{L^q(\Omega )}^2 + A (t-t_0) + \frac{B}{3} (t^3 - t_0^3) \bigg )^{\frac{1}{2}}. \end{aligned}$$

Next, we use the Gagliardo–Nirenberg–Moser interpolation inequality in ${\mathbb {R}}^m$, $m \ge 1$ (see e.g. [23]):

56 $$\begin{aligned} \Vert n-n_\infty \Vert _{L^{\infty }(\Omega )} \le G(\Omega ) \Vert n-n_\infty \Vert _{W^{1,2m}(\Omega )}^{\frac{1}{2}} \Vert n-n_\infty \Vert _{L^{2m}(\Omega )}^{\frac{1}{2}} \,. \end{aligned}$$

Then, interpolating with the exponentially decaying $L^1$-norm of $n-n_\infty$, we obtain

$$\begin{aligned}&\Vert n(t,\cdot )\Vert _{L^{\infty }(\Omega )} \le \Vert n_{\infty }\Vert _{L^{\infty }(\Omega )} + \Vert n-n_{\infty }\Vert _{L^{\infty }(\Omega )} \\&\quad \le \Vert n_{\infty }\Vert _{L^{\infty }(\Omega )} + G\Vert n-n_\infty \Vert _{W^{1,2m}(\Omega )}^{\frac{1}{2}} \Vert n-n_{\infty }\Vert _{L^{\infty }(\Omega )}^{\frac{1}{2} - \frac{1}{4m}} \Vert n-n_{\infty }\Vert _{L^1(\Omega )}^{\frac{1}{4m}}\le Z \end{aligned}$$

due to the exponential convergence to equilibrium (15). The estimate for *p* follows in the same way. $\square$

### Proof of Corollary 1.9

We first notice that exponential convergence of *n* and *p* in $L^q(\Omega )$, $1< q < \infty$, immediately follows from Theorem 1.3 and Corollary 1.8 via

$$\begin{aligned} \Vert n - n_\infty \Vert _{L^q(\Omega )}^q \le \Vert n - n_\infty \Vert _{L^\infty (\Omega )}^{q-1} \Vert n - n_\infty \Vert _{L^1(\Omega )} \le C_q e^{-K_q t} \end{aligned}$$

and an analogous estimate for *p* where $0< C_q, K_q < \infty$ are constants independent of $\varepsilon \in (0, \varepsilon _0]$. Reusing the Gagliardo–Nirenberg–Moser interpolation inequality in ${\mathbb {R}}^m$, $m \ge 1$, from (56) and the polynomial bound on the growth of $\Vert \nabla n (t) \Vert _{L^{2m}(\Omega )}$ from (55), we derive

57 $$\begin{aligned} \Vert n-n_\infty \Vert _{L^{\infty }(\Omega )} \le G(\Omega ) \Vert n-n_\infty \Vert _{W^{1,2m}(\Omega )}^{\frac{1}{2}} \Vert n-n_\infty \Vert _{L^{2m}(\Omega )}^{\frac{1}{2}} \le C_m e^{-K_m t}, \end{aligned}$$

thus, establishing exponential convergence of *n* and *p* in $L^\infty (\Omega )$. Concerning the convergence of $n_{tr}$, we recall the following identities from (25) and (22):

58 $$\begin{aligned} 1 - n_{tr,\infty } =\frac{p_\infty }{p_0 \mu _p} n_{tr,\infty } , \quad n_ {tr,\infty } =\frac{n_\infty }{n_0 \mu _n}(1 - n_{tr,\infty }), \quad \frac{p_\infty }{\mu _p} = p_*, \quad \frac{n_\infty }{\mu _n} = n_*. \end{aligned}$$

We abbreviate $u {:}{=}n_{tr} - n_{tr,\infty }$ and calculate (pointwise in *x*) by adding and subtracting $n_{tr,\infty }$ and $n_{tr}$ multiple times and by using the relations (58):

$$\begin{aligned} \varepsilon \, \partial _t u&= R_p - R_n = \frac{1}{\tau _p} \bigg ( 1 - n_{tr} - \frac{p}{p_0 \mu _p} n_{tr} \bigg ) - \frac{1}{\tau _n} \bigg ( n_{tr} - \frac{n}{n_0 \mu _n} (1 - n_{tr}) \bigg ) \\&= \frac{1}{\tau _p} \bigg ( - u + \frac{p_\infty }{p_0 \mu _p} n_{tr,\infty } - \frac{p}{p_0 \mu _p} n_{tr} \bigg )\\&\quad - \frac{1}{\tau _n} \bigg ( u + \frac{n_\infty }{n_0 \mu _n}(1 - n_{tr,\infty }) - \frac{n}{n_0 \mu _n} (1 - n_{tr}) \bigg ) \\&= - u \underbrace{\left( \frac{1}{\tau _p} + \frac{p_*}{\tau _p p_0} + \frac{1}{\tau _n} + \frac{n_*}{\tau _n n_0}\right) }_{=:K} \\&\quad - \frac{n_{tr}}{\tau _p p_0 \mu _p} \left( p-p_\infty \right) + \frac{(1 - n_{tr})}{\tau _n n_0 \mu _n} \left( n-n_\infty \right) . \end{aligned}$$

Hence, due to $0\le n_{tr}(t, x) \le 1$ for all $t \ge 0$ and a.e. $x \in \Omega$, $\mu _n = e^{-V_n}$, $\mu _p = e^{-V_p}$, and $V = \max ( \Vert V_n \Vert _{L^\infty (\Omega )}, \Vert V_p \Vert _{L^\infty (\Omega )})$, we estimate with (57)

$$\begin{aligned} \frac{d}{dt} \Vert u(t, \cdot )\Vert _{L^\infty (\Omega )}&\le - \frac{K}{\varepsilon } \Vert u(t, \cdot )\Vert _{L^\infty (\Omega )} + \frac{e^V}{\varepsilon \tau _p p_0}\left\| p(t, \cdot )-p_\infty \right\| _{L^\infty (\Omega )} \\&\qquad + \frac{e^V}{\varepsilon \tau _n n_0} \left\| n(t, \cdot )-n_\infty \right\| _{L^\infty (\Omega )}\\&\le - \frac{K}{\varepsilon } \Vert u(t, \cdot )\Vert _{L^\infty (\Omega )} + \frac{C}{\varepsilon } e^{-K_m t} \end{aligned}$$

where $C {:}{=}C_m e^V \left( (\tau _p p_0)^{-1} + (\tau _n n_0)^{-1}\right)$. For the following calculation, we assume w.l.o.g. $2 \varepsilon _0 K_m \le K$. Hence, using $\Vert u(0, \cdot )\Vert _{L^\infty (\Omega )} \le 1$, we arrive at

$$\begin{aligned}&\Vert n_{tr}(t, \cdot ) - n_{tr,\infty } \Vert _{L^\infty (\Omega )} \le e^{-K t/\varepsilon } + \frac{C}{\varepsilon } \int _0^t e^{ -K (t-s) /\varepsilon - K_m s}\,ds \\&\quad \le e^{-K t/\varepsilon } + e^{-K t /\varepsilon } \frac{C}{K- \varepsilon K_m} \left( e^{ (K/\varepsilon - K_m)t} - 1\right) \le e^{-K t/\varepsilon _0} + \frac{C}{K- \varepsilon _0 K_m} e^{ - K_m t}, \end{aligned}$$

where the last bound is independent of $\varepsilon$. $\square$

## A limiting entropy method for system (20)

The existence theory of the Shockley–Read–Hall model applies classical methods (see e.g. [18]) and can be carried out analogously to Theorem 1.1. Therefore, we state here the corresponding results without proof.

### Theorem 7.1

(Shockley–Read–Hall for $\varepsilon = 0$) Under the assumptions of Theorem 1.1, there exists a unique non-negative global weak solution $(n, p) \in \left( C([0, T], L^2(\Omega ))\cap W_2(0,T) \cap L^\infty ((0, T), L^\infty (\Omega ))\right) ^2,$ of system (20) for all $T \in (0, \infty )$ satisfying the boundary conditions (2).

Moreover, there exist positive constants $C_n(\Vert n_I\Vert _{L^\infty (\Omega )},V_n)$, $C_p(\Vert p_I\Vert _{L^\infty (\Omega )},V_p)$ and $K_n(V_n)$, $K_p(V_p)$ such that

59 $$\begin{aligned} \Vert n(t,\cdot )\Vert _{L^\infty (\Omega )} \le C_n + K_nt,\quad \Vert p(t,\cdot )\Vert _{L^\infty (\Omega )} \le C_p + K_pt,\qquad \text {for all } t\ge 0. \end{aligned}$$

Finally, there exist positive constants $\mu$, $\Gamma$, $\theta >0$ (depending on $\tau$, $C_n$, $C_p$, $K_n$, $K_p$, $V_n$, $V_p$) such that

60 $$\begin{aligned} n(t,x), \, p(t,x) \ge \min \Bigl \{\mu t, \frac{\Gamma }{1+\theta t}\Bigr \} \quad \text {for all } t\ge 0 \text { and a.e. } x\in \Omega \end{aligned}$$

where $\mu \tau = \frac{\Gamma }{1+\theta \tau }$ such that the bounds $\mu t$ and $\Gamma /(1 + \theta t)$ intersect at time $\tau$.

The entropy functional (5) extends continuously to the limit $\varepsilon = 0$:

$$\begin{aligned} E_0(n, p) {:}{=}\int _{\Omega } \left( n \ln \frac{n}{n_0 \mu _n} - (n-n_0\mu _n) + p \ln \frac{p}{p_0 \mu _p} - (p-p_0\mu _p) \right) dx, \end{aligned}$$

which is again an entropy (the free energy) functional of the Shockley–Read–Hall model (20). The corresponding entropy production (free energy dissipation) functional reads as

61 $$\begin{aligned} P_0(n,p) {:}{=}-\frac{d}{dt} E_0(n, p) = \int _{\Omega } \left( \frac{|J_n|^2}{n} + \frac{|J_p|^2}{p} - R \ln \left( \frac{np}{n_0 \mu _n p_0\mu _p}\right) \right) dx \ge 0. \end{aligned}$$

Next, we recall from the introduction $n^{qssa}_{tr}=n^{qssa}_{tr}(n,p)$ such that $R_n(n, n_{tr}^{qssa}) = R_p(p, n_{tr}^{qssa})$, i.e.

62 $$\begin{aligned} n^{qssa}_{tr} {:}{=}\frac{\tau _n + \tau _p \frac{n}{n_0 \mu _n}}{\tau _n + \tau _p + \tau _n \frac{p}{p_0 \mu _p} + \tau _p \frac{n}{n_0 \mu _n}}. \end{aligned}$$

$n^{qssa}_{tr}(n,p)$ denotes the pointwise equilibrium value of the trapped states in (1) for fixed *n* and *p*, which corresponds to the quasi-steady-state approximation $\varepsilon = 0$.

Moreover, we observe that the Shockley–Read–Hall entropy production functional (61) can be identified as the entropy production functional $P(n, p, n^{qssa}_{tr})$ as given in (7) along trajectories of (1) with $\varepsilon = 0$ when $n_{tr}\equiv n^{qssa}_{tr}(n,p)$:

$$\begin{aligned} P(n, p, n^{qssa}_{tr})&= \int _{\Omega } \left( \frac{|J_n|^2}{n} + \frac{|J_p|^2}{p} - R_n \ln \left( \frac{n(1-n^{qssa}_{tr})}{n_0 \mu _n n^{qssa}_{tr}} \right) \right. \\&\quad \left. - R_p \ln \left( \frac{p n^{qssa}_{tr}}{p_0 \mu _p (1-n^{qssa}_{tr})} \right) \right) dx \\&= \int _{\Omega } \left( \frac{|J_n|^2}{n} + \frac{|J_p|^2}{p} - R \ln \left( \frac{np}{n_0 \mu _n p_0 \mu _p } \right) \right) dx= P_0(n,p) \end{aligned}$$

where one uses $R=R_n=R_p$ at $n_{tr}=n^{qssa}_{tr}$ and that the involved integrals are finite.

Analogously to Theorem 2.1, there exists a unique equilibrium $(n_{\infty ,0}, p_{\infty ,0}) \in X_0$ in the case $\varepsilon = 0$, where

$$\begin{aligned} X_0:= & {} \{ (n, p) \in H^1(\Omega )^2 \, \big | \, \overline{n} - \overline{p} = M \\&\quad \wedge (\exists \, \gamma > 0) \, n, p \ge \gamma \, \text{ a.e. } \wedge\ n_{tr}^{qssa} \in [\gamma , 1-\gamma ] \, \text{ a.e. }\}. \end{aligned}$$

This equilibrium reads

63 $$\begin{aligned} n_{\infty ,0} = n_{*,0} e^{-V_n}, \quad p_{\infty ,0} = p_{*,0} e^{-V_p}, \end{aligned}$$

where $n_{*,0}, p_{*,0}>0$ are uniquely determined by $n_{*,0} p_{*,0} = n_0 p_0$ and $n_{*,0} \overline{\mu _n} - p_{*,0} \overline{\mu _p} = M$.

We are now in the position to formulate the EEP-inequality.

### Theorem 7.2

(Entropy–Entropy Production Inequality for $\varepsilon = 0$) Let $\tau _n$, $\tau _p$, $n_0$, $p_0$, $M_1$ and *V* be positive constants. Consider $M\in {\mathbb {R}}$ and the correspondingly unique equilibrium $(n_{\infty ,0}, p_{\infty ,0}) \in X_0$.

Then, the following EEP-inequality holds true for all non-negative functions $(n,p) \in L^1(\Omega )^2$ satisfying the conservation law $\overline{n} - \overline{p} = M,$ the $L^1$-bound $\overline{n}, \overline{p} < M_1$ as well as the conditions $E_0(n, p)<\infty$, $P_0(n, p)<\infty$, $P(n, p, n^{qssa}_{tr}) < \infty$ for some $\varepsilon _0 > 0$:

64 $$\begin{aligned} E_0(n,p) - E_0(n_{\infty ,0}, p_{\infty ,0}) \le C_{\mathrm {EEP}} P_0(n,p), \end{aligned}$$

where $C_{\mathrm {EEP}} > 0$ is the same constant as in Theorem 1.5.

### Theorem 7.3

(Exponential convergence for $\varepsilon = 0$) Let (*n*, *p*) be a global weak solution of system (20) as given in Theorem 7.1 corresponding to the non-negative initial data $(n_I,p_I) \in L^\infty (\Omega )^2$. Then, this solution satisfies the entropy production law

65 $$\begin{aligned} E_0(n,p)(t_1) + \int _{t_0}^{t_1} P_0(n, p)(s) \, ds = E_0(n,p)(t_0) \end{aligned}$$

for all $$0< t_0 \le t_1 < \infty$$.

Moreover, the following versions of the exponential decay towards the equilibrium $(n_{\infty ,0}, p_{\infty ,0}) \in X_0$ hold true:

$$\begin{aligned} E_0(n,p)(t) - E_\infty \le (E_I - E_\infty ) e^{-K t} \end{aligned}$$

and

66 $$\begin{aligned} \Vert n - n_{\infty ,0} \Vert _{L^1(\Omega )}^2 + \Vert p - p_{\infty ,0} \Vert _{L^1(\Omega )}^2 \le C (E_I - E_\infty ) e^{-K t} \end{aligned}$$

where $C := C_{\mathrm {CKP}}^{-1}$ and $K := C_{\mathrm {EEP}}^{-1}$ are the same constants as in Theorem 1.3. Moreover, $E_I$ and $E_\infty$ denote the initial entropy of the system and the entropy in the equilibrium, respectively.

### Remark 7.4

We believe that the entropy–entropy production inequality (64) can also be proven by combining estimates of Sect. 5 with previous works on the entropy method for detailed balanced reaction–diffusion models, see e.g. [5, 7, 10, 19]. We emphasise, however, that our goal with Theorem 7.2 is to be able to derive an entropy–entropy production inequality via the fast-reaction parameter $\varepsilon \rightarrow 0$.

Finally, in the same way as for strictly positive $\varepsilon > 0$, we can derive uniform-in-time $L^\infty$-bounds for *n* and *p* also in the case $\varepsilon = 0$. As before, these bounds further improve the lower bounds on *n* and *p*.

### Corollary 7.5

There exists a constant $Z > 0$ such that

67 $$\begin{aligned} \Vert n(t,\cdot )\Vert _{L^\infty (\Omega )} , \Vert p(t,\cdot )\Vert _{L^\infty (\Omega )}\le Z \quad \text {for all } t\ge 0. \end{aligned}$$

And for all $\tau >0$ there exist sufficiently small constants $\mu , \Gamma > 0$ such that

68 $$\begin{aligned} n(t,x),p(t,x) \ge \min \left\{ \mu t, \Gamma \right\} \end{aligned}$$

for all $t\ge 0$ and a.e. $x\in \Omega$, where $\mu \tau =\Gamma$ such that the bounds $\mu t$ and $\Gamma$ intersect at time $\tau > 0$.

### Corollary 7.6

Under the hypotheses of Theorem 7.3, there exist constants $0< C, K < \infty$ such that

$$\begin{aligned} \Vert n - n_\infty \Vert _{L^\infty (\Omega )} + \Vert p - p_\infty \Vert _{L^\infty (\Omega )} \le C e^{-Kt} \end{aligned}$$

holds true for all $t \ge 0$.

### Proof of Theorem 1.5

Our goal is to derive an estimate of the form

$$\begin{aligned} E_0(n, p) - E_0(n_{\infty ,0}, p_{\infty ,0}) \le C_{EEP} P_0(n, p) \end{aligned}$$

by applying the EEP-inequality from Theorem 1.5 directly to the functions *n*, *p* and $n^{qssa}_{tr}$. However, since we assume that *n* and *p* satisfy

$$\begin{aligned} \overline{n} - \overline{p} = M, \end{aligned}$$

the triple $(n, p, n^{qssa}_{tr})$ does not satisfy the conservation law with right hand side *M* but

$$\begin{aligned} \overline{n} - \overline{p} + \varepsilon \overline{n^{qssa}_{tr}} = M + \varepsilon \overline{n^{qssa}_{tr}}. \end{aligned}$$

In order to resolve this issue, we shall apply the EEP-inequality from Theorem 1.5 to a suitably defined sequence of functions $(n_\varepsilon , p_\varepsilon , n_{tr,\varepsilon }) \in L^1(\Omega )^3$ which fulfil $\Vert n_{tr,\varepsilon } \Vert _{L^\infty (\Omega )} \le 1$, the $L^1$-bound $\overline{n_\varepsilon }, \overline{p_\varepsilon } \le M_1$ and the conservation law

$$\begin{aligned} \overline{n_\varepsilon } - \overline{p_\varepsilon } + \varepsilon \overline{n_{tr,\varepsilon }} = M. \end{aligned}$$

A convenient choice is $n_\varepsilon {:}{=}n$, $p_\varepsilon {:}{=}p + \varepsilon \overline{n_{tr}^{qssa}}$ and $n_{tr,\varepsilon } {:}{=}n^{qssa}_{tr}$, where $n^{qssa}_{tr}=n^{qssa}_{tr}(n,p)$ as defined in (62). For this choice, we derive the stated EEP-estimate for the case $\varepsilon = 0$ via the following steps, which are proven below:

69 $$\begin{aligned} E_0(n, p) - E_0(n_{\infty ,0}, p_{\infty ,0})&= \lim \limits _{\varepsilon \rightarrow 0} \big ( E(n_\varepsilon , p_\varepsilon , n_{tr,\varepsilon }) - E(n_\infty , p_\infty , n_{tr,\infty }) \big ) \end{aligned}$$

70 $$\begin{aligned}&\le \lim \limits _{\varepsilon \rightarrow 0} \big ( C_{EEP} P(n_\varepsilon , p_\varepsilon , n_{tr,\varepsilon }) \big ) \end{aligned}$$

71 $$\begin{aligned}&= C_{EEP} P(n, p, n^{qssa}_{tr}) = C_{EEP} P_0(n, p) \end{aligned}$$

We recall that *n* and *p* are assumed to satisfy $E_0(n, p)<\infty$ and $P_0(n, p),P(n, p, n^{qssa}_{tr})<\infty$, which implies that $P_0(n, p)=P(n, p, n^{qssa}_{tr})$ as discussed in the introduction.

*Step 1. Proof of* (69): We first show, that with $(n_\varepsilon , p_\varepsilon , n_{tr,\varepsilon })=(n, p_\varepsilon , n^{qssa}_{tr})$

72 $$\begin{aligned} E_0(n, p) = \lim \limits _{\varepsilon \rightarrow 0} E(n_\varepsilon , p_\varepsilon , n_{tr,\varepsilon }). \end{aligned}$$

Recalling that

$$\begin{aligned} E(n,p_\varepsilon ,n^{qssa}_{tr})= & {} \int _{\Omega } \left( n \ln \frac{n}{n_0 \mu _n} - (n-n_0\mu _n)+ p_\varepsilon \ln \frac{p_\varepsilon }{p_0 \mu _p} \right. \\&\left. - (p_\varepsilon -p_0\mu _p) + \varepsilon \int _{1/2}^{n^{qssa}_{tr}} \ln \left( \frac{s}{1-s} \right) ds \right) dx, \end{aligned}$$

we first notice that $p_\varepsilon =p + \varepsilon \overline{n_{tr}^{qssa}} \rightarrow p$ monotonically decreasing for $\varepsilon \rightarrow 0$ for all $x\in \Omega$. Thus, by using $\overline{n_{tr}^{qssa}} \le 1$ and the elementary estimate $p_\varepsilon \ln p_\varepsilon \le 2 p\, (\ln p + \ln 2)$ for $p\ge \max \{\varepsilon _0,1\}$, the Lebesgue dominated convergence theorem, the $L^1$-bounds $\overline{n},\overline{n_\varepsilon }, \overline{p},\overline{p_\varepsilon } \le M_1$ and $E_0(n, p)<\infty$ imply the convergence of the $p_\varepsilon$-integral in (72). The convergence of the third integral follows directly from

$$\begin{aligned} \left| \varepsilon \int _{1/2}^{n^{qssa}_{tr}(x)} \ln \frac{s}{1-s} \, ds \right| \le \varepsilon \int _{1/2}^1 \ln \frac{s}{1-s} \, ds \xrightarrow {\varepsilon \rightarrow 0} 0. \end{aligned}$$

Using analogue arguments, the convergence

$$\begin{aligned} E_0(n_{\infty ,0}, p_{\infty ,0}) = \lim \limits _{\varepsilon \rightarrow 0} E(n_\infty , p_\infty , n_{tr,\infty }) \end{aligned}$$

follows from observing the monotone convergence $n_*\rightarrow n_{*,0}$ and $p_*\rightarrow p_{*,0}$ for $\varepsilon \rightarrow 0$ due to (27) in the proof of Theorem 2.1, which directly implies the monotone convergence $n_\infty \rightarrow n_{\infty ,0}$ and $p_\infty \rightarrow p_{\infty ,0}$ for all $x\in \Omega$, where $(n_\infty , p_\infty , n_{tr,\infty })$ and $(n_{\infty ,0}, p_{\infty ,0})$ are defined in (22) and (63), respectively.

*Step 2. Proof of* (70): We observe that the functions $(n_\varepsilon , p_\varepsilon , n_{tr,\varepsilon }) = (n, p + \varepsilon \overline{n_{tr}^{qssa}}, n^{qssa}_{tr}) \in L^1(\Omega )^3$ satisfy $\Vert n_{tr,\varepsilon } \Vert _{L^\infty (\Omega )} \le 1$, the conservation law

$$\begin{aligned} \overline{n}_{\varepsilon } - \overline{p_\varepsilon } + \varepsilon \overline{n_{tr,\varepsilon }} = \overline{n} - \overline{p} = M \end{aligned}$$

as well as the $L^1$-bounds $\overline{n}_{\varepsilon } \le M_1$ and $\overline{p_\varepsilon } \le \overline{p} + \varepsilon '$ where $\varepsilon \in (0, \varepsilon '] \subset (0, \varepsilon _0]$. Because of $\overline{p} < M_1$, we have $\overline{p_\varepsilon } \le M_1$ for $\varepsilon ' > 0$ sufficiently small. As a consequence,

$$\begin{aligned} E(n_\varepsilon , p_\varepsilon , n_{tr,\varepsilon }) - E(n_\infty , p_\infty , n_{tr,\infty }) \le C_{EEP} P(n_\varepsilon , p_\varepsilon , n_{tr,\varepsilon }) \end{aligned}$$

where $C_{EEP} > 0$ is the same constant as in Theorem 1.5.

*Step 3. Proof of* (71): As the constant $C_{EEP} > 0$ is independent of $\varepsilon \in (0, \varepsilon _0]$, it suffices to show that

$$\begin{aligned} \lim \limits _{\varepsilon \rightarrow 0} P(n_\varepsilon , p_\varepsilon , n_{tr,\varepsilon }) = P(n, p, n^{qssa}_{tr}). \end{aligned}$$

To this end, we consider the representation

$$\begin{aligned} P(n_\varepsilon , p_\varepsilon , n_{tr,\varepsilon })= & {} \int _{\Omega } \bigg (\frac{|J_n|^2}{n} + \frac{|\nabla p|^2}{p_\varepsilon } + 2 \nabla p \cdot \nabla V_p + p_\varepsilon |\nabla V_p|^2 \\&- R_n \ln \left( \frac{n(1-n^{qssa}_{tr})}{n_0 \mu _n n^{qssa}_{tr}} \right) + \frac{1}{\tau _p} \biggl ( \frac{p_\varepsilon }{p_0 \mu _p} n^{qssa}_{tr} - (1 - n^{qssa}_{tr}) \biggr ) \\&\times \biggl ( \ln \frac{p_\varepsilon n^{qssa}_{tr}}{p_0 \mu _p} - \ln (1 - n^{qssa}_{tr}) \biggr ) \bigg ) \, dx, \end{aligned}$$

where we have already taken into account that $n_\varepsilon =n$, $\nabla p_\varepsilon = \nabla p$ and $n_{tr,\varepsilon }=n^{qssa}_{tr}$ for all $\varepsilon > 0$.

We note first that the convergence of the second, third and forth integral follows from the pointwise convergence of $p_{\varepsilon }$ for all $x\in \Omega$ and from the Lebesgue dominated convergence theorem by estimating

$$\begin{aligned} 0\le & {} \frac{|\nabla p|^2}{p_\varepsilon } + 2 \nabla p \cdot \nabla V_p + p_\varepsilon |\nabla V_p|^2 \le \frac{|\nabla p|^2}{p} + 2 \nabla p \cdot \nabla V_p + p |\nabla V_p|^2 \\&\quad + (p_\varepsilon - p) |\nabla V_p|^2 \le \frac{|J_p|^2}{p} + \varepsilon _0 \overline{n^{qssa}_{tr}} |\nabla V_p|^2, \end{aligned}$$

where the function on the right hand side is integrable due to the finiteness of $P(n, p, n^{qssa}_{tr})$.

Secondly, the product

$$\begin{aligned}&\left( \frac{p_\varepsilon }{p_0 \mu _p} n^{qssa}_{tr} - (1 - n^{qssa}_{tr}) \right) \left( \ln \frac{p_\varepsilon n^{qssa}_{tr}}{p_0 \mu _p} -\ln (1 - n^{qssa}_{tr}) \right) \\&\quad \longrightarrow \left( \frac{p}{p_0 \mu _p} n^{qssa}_{tr} - (1 - n^{qssa}_{tr}) \right) \!\left( \ln \frac{p n^{qssa}_{tr}}{p_0 \mu _p} -\ln (1 - n^{qssa}_{tr}) \right) \end{aligned}$$

converges pointwise for all $x\in \Omega$ as $\varepsilon \rightarrow 0$. In order to conclude the convergence of the corresponding integral via the Lebesgue dominated convergence theorem, we use similar to Step 1 the elementary inequality $p_\varepsilon \ln p_\varepsilon \le 2 p\, (\ln p + \ln 2)$ for $p\ge \max \{\varepsilon _0,1\}$ and the finiteness of $P(n, p, n^{qssa}_{tr})$. This yields

$$\begin{aligned}&\lim \limits _{\varepsilon \rightarrow 0} \int _\Omega \frac{1}{\tau _p} \left( \frac{p_\varepsilon }{p_0 \mu _p} n^{qssa}_{tr}\!-\!(1 - n^{qssa}_{tr}) \right) \!\left( \ln \frac{p_\varepsilon n^{qssa}_{tr}}{p_0 \mu _p} -\ln (1 - n^{qssa}_{tr}) \right) \! dx \\&\quad = -\int _\Omega R_p \ln \left( \frac{p n^{qssa}_{tr}}{p_0 \mu _p (1-n^{qssa}_{tr})} \right) \! dx \end{aligned}$$

and therefore, $P(n_\varepsilon , p_\varepsilon , n_{tr,\varepsilon }) \rightarrow P(n, p, n^{qssa}_{tr})$ for $\varepsilon \rightarrow 0$. $\square$

### Proof of Theorem 1.3

We only have to check that the assumptions on the finiteness of the entropy $E_0$ and the entropy production functionals $P_0$ and *P* within Theorem 2 are satisfied. The claim of this theorem then follows from the same arguments as in the proof of Theorem 1.3.

Due to the uniform $L^\infty$-bounds (59) of *n*(*t*) and *p*(*t*) for all $t \ge 0$, we know that $E_0(n, p) < \infty$ for all $t \ge 0$. Similarly, we deduce that $P(n, p, n^{qssa}_{tr})$ and $P_0(n, p)$ are finite for all strictly positive $t > 0$ since *n*, *p* are bounded away from zero and $n^{qssa}_{tr}$ is bounded away from zero and one uniformly in $\Omega$.

Finally, the lower bounds (60) guarantee similar to Theorem 1.5 that solutions satisfy the weak entropy production law (65) for all $t_0 > 0$. $\square$

## Conclusion

We have investigated the drift–diffusion–recombination system (1) modelling the transport, generation and annihilation of negatively charged electrons and positively charged holes (vacancies of electrons) in certain types of semiconductors. As depicted in Fig. 1, we have considered a two-level system augmented by an additional intermediate energy level, the so-called trap level, which results from the presence of foreign atoms inside the crystal of the semiconductor. We have derived an entropy–entropy production (EEP) inequality (cf. Theorem 1.5) which bounds the entropy functional (5) from above in terms of the entropy production functional (7). This EEP-inequality has then be used to show that the concentrations of electrons and holes converge to their equilibrium distributions at an exponential rate as time tends to infinity (cf. Theorem 1.3).

A novel achievement of our studies is the fact that the entropy method has been applied uniformly in a small time-related parameter. More precisely, the constant $C_\mathrm {EEP}$ in Theorem 1.5 is independent of the lifetime $\varepsilon$ of electrons on the trap level (cf. (1)) provided $\varepsilon \in (0, \varepsilon _0]$ for some $\varepsilon _0 > 0$. The $\varepsilon$-independence of $C_\mathrm {EEP}$ transfers to the constants appearing in the exponential decay estimate in Theorem 1.3. This proves that the exponential convergence rate is independent of a quasi-steady-state approximation of the electrons on the trap level, which leads to the famous Shockley–Read–Hall recombination model [16, 21].

In particular, we were able to derive an EEP-inequality and the convergence estimate for the limiting Shockley–Read–Hall model. This fact is notable from a conceptual point of view as we transfer the results for $\varepsilon > 0$ to the case $\varepsilon = 0$ by performing the limit $\varepsilon \rightarrow 0$. We believe that our limiting approach to the Shockley–Read–Hall model may serve as an example for possible applications of this technique to fast-reaction limits and quasi-steady-state approximations.

In view of the technicalities of the proofs and the resulting length of the current paper, our results are still limited by not taking into account the self-consistent potential generated by electrons and holes, which is required by a physically more precise model. However, this leads to an additional coupling of (1) to Poisson’s equation and a further increase in complexity of the problem. We expect however to resolve these issues in a future work by combining techniques and results presented in the current paper with ideas in [9], which considered a self-consistent Shockley–Read–Hall model without trapped states.

## Appendix: Proof of the existence theorem

### Proof of Theorem 1.1

In order to simplify the notation, we set the parameter $\tau _n := \tau _p := 1$ and $n_0 := p_0 := 1$ throughout the proof. All arguments also apply in the case of arbitrary values for $\tau _n$, $\tau _p$, $n_0$ and $p_0$. The structure of system (1) can be further simplified via introducing new variables

$$\begin{aligned} u := e^\frac{V_n}{2}n, \quad v := e^\frac{V_p}{2}p. \end{aligned}$$

One obtains

$$\begin{aligned} \nabla u= & {} \frac{1}{2} e^\frac{V_n}{2} \nabla V_n n + e^\frac{V_n}{2} \nabla n \quad \text{ and } \\ \Delta u= & {} e^\frac{V_n}{2} \Big ( \Delta n + \nabla n \cdot \nabla V_n + \frac{1}{4} n |\nabla V_n|^2 + \frac{1}{2} n \Delta V_n \Big ) \end{aligned}$$

which results in

$$\begin{aligned} \partial _t u&= e^\frac{V_n}{2} \partial _t n = e^\frac{V_n}{2} \Big ( \Delta n + \nabla n \cdot \nabla V_n + n \Delta V_n + R_n \Big ) \\&= \Delta u - e^\frac{V_n}{2} \left( \frac{1}{4} n |\nabla V_n|^2 - \frac{1}{2} n \Delta V_n \right) + e^\frac{V_n}{2} R_n \\&= \Delta u + \left( \frac{1}{2} \Delta V_n - \frac{1}{4} |\nabla V_n|^2 \right) u + e^\frac{V_n}{2} n_{tr} - e^{V_n} u (1 - n_{tr}). \end{aligned}$$

Analogously, we derive

$$\begin{aligned} \partial _t v = \Delta v + \left( \frac{1}{2} \Delta V_p - \frac{1}{4} |\nabla V_p|^2 \right) v + e^\frac{V_p}{2} (1-n_{tr}) - e^{V_p} v n_{tr}. \end{aligned}$$

For convenience, we also introduce the abbreviations

$$\begin{aligned} A_n := \frac{1}{2} \Delta V_n - \frac{1}{4} |\nabla V_n|^2 \in L^\infty (\Omega ), \quad A_p := \frac{1}{2} \Delta V_p - \frac{1}{4} |\nabla V_p|^2 \in L^\infty (\Omega ) \end{aligned}$$

as well as $\alpha , \beta > 0$ such that the following estimates hold true a.e. in $\Omega$:

$$\begin{aligned} |A_n|, |A_p| \le \alpha \quad \text{ and } \quad e^\frac{V_n}{2}, e^\frac{V_p}{2}, e^{V_n}, e^{V_p} \le \beta . \end{aligned}$$

Next, we introduce the new variable

73 $$\begin{aligned} n_{tr}' := 1 - n_{tr} \end{aligned}$$

for reasons of symmetry. In fact, we can prove the positivity of $n_{tr}'$ in the same way as for $n_{tr}$, which then implies the desired bound $0 \le n_{tr} \le 1$. A further ingredient for establishing the positivity of the variables *u*, *v*, $n_{tr}$ and $n_{tr}'$ is to project them onto $[0, \infty )$ and [0, 1], respectively, on the right hand side of the PDE-system. In this context, we use $X^+\! := \max (X, 0)$ to denote the positive part of an arbitrary function *X* and $X^{[0,1]} := \min (\max (X, 0), 1)$ for the projection of *X* to the interval [0, 1]. The modified system now reads

74 $$\begin{aligned} {\left\{ \begin{array}{ll} \begin{aligned} \partial _t u - \Delta u &{}= A_n u^+ + e^\frac{V_n}{2} n_{tr}^{[0,1]} - e^{V_n} u^+ n_{tr}'^{[0,1]}, \\ \partial _t v - \Delta v &{}= A_p v^+ + e^\frac{V_p}{2} n_{tr}'^{[0,1]} - e^{V_p} v^+ n_{tr}^{[0,1]}, \\ \varepsilon \, \partial _t n_{tr} &{}= n_{tr}'^{[0,1]} - e^\frac{V_p}{2} v^+ n_{tr}^{[0,1]} - n_{tr}^{[0,1]} + e^\frac{V_n}{2} u^+ n_{tr}'^{[0,1]}, \\ \varepsilon \, \partial _t n_{tr}' &{}= n_{tr}^{[0,1]} - e^\frac{V_n}{2} u^+ n_{tr}'^{[0,1]} - n_{tr}'^{[0,1]} + e^\frac{V_p}{2} v^+ n_{tr}^{[0,1]}. \end{aligned} \end{array}\right. } \end{aligned}$$

The no-flux boundary conditions of (1) transfer to similar conditions on *u* and *v*. In detail, we have

$$\begin{aligned} e^{-\frac{V_n}{2}} \nabla u = \nabla n + \frac{1}{2} n \nabla V_n \end{aligned}$$

and, hence,

$$\begin{aligned} \nabla n + n \nabla V_n = e^{-\frac{V_n}{2}} \Big ( \nabla u + \frac{1}{2} u \nabla V_n \Big ). \end{aligned}$$

Therefore, the corresponding boundary conditions for *u* and *v* read

75 $$\begin{aligned} {\hat{n}} \cdot \Big (\nabla u + \frac{1}{2} u \nabla V_n \Big ) = {\hat{n}} \cdot \Big (\nabla v + \frac{1}{2} v \nabla V_p \Big ) = 0. \end{aligned}$$

Furthermore, we assume that the corresponding initial states satisfy

76 $$\begin{aligned} (u_I, v_I, n_{tr,I}, n_{tr,I}') \in L_+^\infty (\Omega )^4, \quad n_{tr,I} + n_{tr,I}' = 1. \end{aligned}$$

In this situation, $\Vert n_{tr,I}\Vert _{L^\infty (\Omega )} + \Vert n_{tr,I}'\Vert _{L^\infty (\Omega )} \ge 1$ and we set

$$\begin{aligned} I := \Vert u_I\Vert _{L^\infty (\Omega )} + \Vert v_I\Vert _{L^\infty (\Omega )} + \Vert n_{tr,I}\Vert _{L^\infty (\Omega )} + \Vert n_{tr,I}'\Vert _{L^\infty (\Omega )} \ge 1. \end{aligned}$$

We now aim to apply Banach’s fixed-point theorem to obtain a solution of (74)–(76).

*Step 1: Definition of the fixed-point iteration.* For any time $T > 0$ (to be chosen sufficiently small in the course of the fixed-point argument), we introduce the space

$$\begin{aligned} X_T := C([0, T], L^2(\Omega ))^4 \end{aligned}$$

and the closed subspace

$$\begin{aligned} M_T &:= \big \{ (u, v, n_{tr}, n_{tr}') \in X_T \, \big | \, (u(0), v(0), n_{tr}(0), n_{tr}'(0)) = (u_I, v_I, n_{tr,I}, n_{tr,I}'), \\&\qquad \max _{0 \le t \le T} \Vert u(t) \Vert _{L^2(\Omega )},\max _{0 \le t \le T} \Vert v(t) \Vert _{L^2(\Omega )}, \, \max _{0 \le t \le T} \Vert n_{tr}(t) \Vert _{L^2(\Omega )}, \\&\qquad \max _{0 \le t \le T} \Vert n_{tr}'(t) \Vert _{L^2(\Omega )}, \Vert u \Vert _{L^\infty ((0, T) \times \Omega )}, \Vert v \Vert _{L^\infty ((0, T) \times \Omega )} \le 2I \big \} \subset X_T. \end{aligned}$$

The fixed-point mapping ${\mathcal {S}}: X_T \rightarrow X_T$ is now defined via

$$\begin{aligned} {\mathcal {S}}(\widetilde{u}, \widetilde{v}, \widetilde{n}_{tr}, \widetilde{n}_{tr}') := (u, v, n_{tr}, n_{tr}') \end{aligned}$$

where $(u, v, n_{tr}, n_{tr}')$ is the solution of the following PDE-system subject to the boundary and initial conditions specified above:

77 $$\begin{aligned} {\left\{ \begin{array}{ll} \begin{aligned} \partial _t u - \Delta u &{}= A_n \widetilde{u}^+ + e^\frac{V_n}{2} \widetilde{n}_{tr}^{[0,1]} - e^{V_n} \widetilde{u}^+ \widetilde{n}_{tr}'^{[0,1]} =: \widetilde{f}_1, \\ \partial _t v - \Delta v &{}= A_p \widetilde{v}^+ + e^\frac{V_p}{2} \widetilde{n}_{tr}'^{[0,1]} - e^{V_p} \widetilde{v}^+ \widetilde{n}_{tr}^{[0,1]} =: \widetilde{f}_2, \\ \varepsilon \, \partial _t n_{tr} &{}= \widetilde{n}_{tr}'^{[0,1]} - e^\frac{V_p}{2} \widetilde{v}^+ \widetilde{n}_{tr}^{[0,1]} - \widetilde{n}_{tr}^{[0,1]} + e^\frac{V_n}{2} \widetilde{u}^+ \widetilde{n}_{tr}'^{[0,1]} =: \widetilde{f}_3, \\ \varepsilon \, \partial _t n_{tr}' &{}= \widetilde{n}_{tr}^{[0,1]} - e^\frac{V_n}{2} \widetilde{u}^+ \widetilde{n}_{tr}'^{[0,1]} - \widetilde{n}_{tr}'^{[0,1]} + e^\frac{V_p}{2} \widetilde{v}^+ \widetilde{n}_{tr}^{[0,1]} =: \widetilde{f}_4. \end{aligned} \end{array}\right. } \end{aligned}$$

We first show that $(u, v, n_{tr}, n_{tr}') = {\mathcal {S}}(\widetilde{u}, \widetilde{v}, \widetilde{n}_{tr}, \widetilde{n}_{tr}') \in X_T$ provided $(\widetilde{u}, \widetilde{v}, \widetilde{n}_{tr}, \widetilde{n}_{tr}') \in X_T$. Due to $\widetilde{f}_1$, $\widetilde{f}_2 \in L^2((0, T) \times \Omega )$, it is known from classical PDE-theory (see e.g. [3]) that

$$\begin{aligned} u, v \in W_2(0, T)= & {} \big \{ f \in L^2((0, T), H^1(\Omega )) \, | \, \partial _t f \in L^2((0, T), H^1(\Omega )^*) \big \} \\&\hookrightarrow C([0, T], L^2(\Omega )). \end{aligned}$$

And since

$$\begin{aligned} n_{tr}(t) = n_{tr}(0) + \frac{1}{\varepsilon } \int _0^t \widetilde{f}_3(s) \, ds \end{aligned}$$

for almost all $t \in [0, T]$, we deduce

$$\begin{aligned} \Vert n_{tr}(t)\Vert _{L^2(\Omega )}\le & {} \Vert n_{tr}(0)\Vert _{L^2(\Omega )} + \frac{1}{\varepsilon } \int _0^t \Vert \widetilde{f}_3(s)\Vert _{L^2(\Omega )} \, ds\\\le & {} I + \frac{T}{\varepsilon } \max _{0 \le t \le T} \Vert \widetilde{f}_3(s) \Vert _{L^2(\Omega )}. \end{aligned}$$

Hence, $n_{tr} \in L^\infty ((0, T), L^2(\Omega ))$. Moreover, we derive for $[0, T] \ni t_k \rightarrow t \in [0, T]$,

$$\begin{aligned} \Vert n_{tr}(t_k) - n_{tr}(t) \Vert _{L^2(\Omega )}\le & {} \frac{1}{\epsilon } \left| \int _t^{t_k} \Vert \widetilde{f}_3(s) \Vert _{L^2(\Omega )} \, ds \right| \\\le & {} \frac{|t_k - t|}{\epsilon } \max _{0 \le t \le T} \Vert \widetilde{f}_3(s) \Vert _{L^2(\Omega )} \xrightarrow {k\rightarrow \infty } 0. \end{aligned}$$

This proves $n_{tr} \in C([0, T], L^2(\Omega ))$. The same arguments can be applied to $n_{tr}'$.

*Step 2: Invariance of* $M_T$. Now, let $(\widetilde{u}, \widetilde{v}, \widetilde{n}_{tr}, \widetilde{n}_{tr}') \in M_T$. Similar to the strategy of e.g. [1, 15, 26], we perform the subsequent calculations for any $q \in 2 {\mathbb {N}}_+$ and every $t \in [0, T]$:

$$\begin{aligned} \int _0^t \frac{d}{ds} \int _\Omega \frac{u^q}{q} \, dx \, ds= & {} \int _0^t \int _\Omega u^{q-1} \partial _t u \, dx \, ds = \int _0^t \int _\Omega u^{q-1} \Delta u \, dx \, ds \\&+ \int _0^t \int _\Omega u^{q-1} \widetilde{f}_1 \, dx \, ds \le -(q-1) \int _0^t \int _\Omega u^{q-2} |\nabla u|^2 \, dx \, ds\\&- \frac{1}{2} \int _0^t \int _{\partial \Omega } u^q \, {\hat{n}} \cdot \nabla V_n \, d\sigma ds + \Vert \widetilde{f}_1 \Vert _{L^\infty ((0, T) \times \Omega )} \int _0^t \int _\Omega |u|^{q-1} dx ds \\\le & {}\ (2 \alpha I + \beta + 2 \beta I) \int _0^t \Vert u\Vert _{L^q(\Omega )}^{q-1} \, ds. \end{aligned}$$

Note that the last term in the second and the first term in the third line are both non-positive due to $q \in 2{\mathbb {N}}$ and assumption (4). Introducing $\gamma := 2 \alpha I + \beta + 2 \beta I$, we obtain

78 $$\begin{aligned} \Vert u(t) \Vert _{L^q(\Omega )}^q - \Vert u(0) \Vert _{L^q(\Omega )}^q \le q \gamma \int _0^t \Vert u(s) \Vert _{L^q(\Omega )}^{q-1} \, ds. \end{aligned}$$

This inequality already implies a linear bound on the $L^\infty$-norm of *u* as we shall see below (cf. [2]). We define

$$\begin{aligned} U(t) := q\gamma \int _0^t \Vert u(s) \Vert _{L^q(\Omega )}^{q-1} \, ds \end{aligned}$$

and note that $U(0) = 0$. Estimate (78) entails

$$\begin{aligned} U'(t) = q\gamma \Big ( \Vert u(t) \Vert _{L^q(\Omega )}^{q} \Big )^\frac{q-1}{q} \le q\gamma \Big ( \eta + \Vert u(0) \Vert _{L^q(\Omega )}^{q} + U(t) \Big )^\frac{q-1}{q} \end{aligned}$$

for all $t \in [0, T]$, where $\eta > 0$ is an arbitrary constant, which guarantees that the expression $X:=\eta + \Vert u(0) \Vert _{L^q(\Omega )}^{q} + U(t)$ is strictly positive. Multiplying both sides with $X^{(1-q)/q}$ and integrating from 0 to *t* gives

$$\begin{aligned} \int _0^t \Big ( \eta + \Vert u(0) \Vert _{L^q(\Omega )}^{q} + U(s) \Big )^\frac{1-q}{q} U'(s) \, ds \le \int _0^t q\gamma \, ds. \end{aligned}$$

We now substitute $\sigma := U(s)$ and deduce

$$\begin{aligned} q\gamma t &\ge \int _0^{U(t)} \Big ( \eta + \Vert u(0) \Vert _{L^q(\Omega )}^{q} + \sigma \Big )^{\frac{1}{q}-1} \, d\sigma = q \Big ( \eta + \Vert u(0) \Vert _{L^q(\Omega )}^{q} + \sigma \Big )^\frac{1}{q} \, \Big |_0^{U(t)} \\&= q \Big ( \eta + \Vert u(0) \Vert _{L^q(\Omega )}^{q} + U(t) \Big )^\frac{1}{q} - q \Big ( \eta + \Vert u(0) \Vert _{L^q(\Omega )}^{q} \Big )^\frac{1}{q} \\&\ge q \Big ( \Vert u(t) \Vert _{L^q(\Omega )}^{q} \Big )^\frac{1}{q} - q \Big ( \eta + \Vert u(0) \Vert _{L^q(\Omega )}^{q} \Big )^\frac{1}{q} \end{aligned}$$

where we have used (78) in the last step. Therefore,

$$\begin{aligned} \Vert u(t) \Vert _{L^q(\Omega )} \le \Big ( \eta + \Vert u(0) \Vert _{L^q(\Omega )}^{q} \Big )^\frac{1}{q} + \gamma t \end{aligned}$$

and, taking the limit $\eta \rightarrow 0$,

$$\begin{aligned} \Vert u(t) \Vert _{L^q(\Omega )} \le \Vert u(0) \Vert _{L^q(\Omega )} + \gamma t \le I + \gamma t. \end{aligned}$$

As the bound on the right hand side is independent of *q*, we even obtain

79 $$\begin{aligned} \Vert u(t) \Vert _{L^\infty (\Omega )} \le I + \gamma t, \end{aligned}$$

for all $t \in [0, T]$. This result naturally gives rise to

$$\begin{aligned} \Vert u\Vert _{L^\infty ((0, T) \times \Omega )} \le I + \gamma T. \end{aligned}$$

An analogous estimate is valid for *v*. As a result, we obtain

$$\begin{aligned} \Vert u \Vert _{L^\infty ((0, T) \times \Omega )}, \, \Vert v \Vert _{L^\infty ((0, T) \times \Omega )} \le 2I \end{aligned}$$

for $T > 0$ chosen sufficiently small.

Employing (79), we also derive

$$\begin{aligned} \max _{0 \le t \le T} \Vert u(t) \Vert _{L^2(\Omega )} \le \max _{0 \le t \le T} \Vert u(t) \Vert _{L^\infty (\Omega )} \le I + \gamma T. \end{aligned}$$

The same argument is applicable to *v*, which results in

$$\begin{aligned} \max _{0 \le t \le T} \Vert u(t) \Vert _{L^2(\Omega )}, \, \max _{0 \le t \le T} \Vert v(t) \Vert _{L^2(\Omega )} \le 2 I \end{aligned}$$

for sufficiently small $T>0$. The corresponding bounds on $n_{tr}$ and $n_{tr}'$ can be deduced from the formula

$$\begin{aligned} n_{tr}(t) = n_{tr}(0) + \frac{1}{\varepsilon } \int _0^t \widetilde{f}_3(s) \, ds \end{aligned}$$

and from an analogous one for $n_{tr}'$. In fact,

$$\begin{aligned} \Vert n_{tr}(t) \Vert _{L^2(\Omega )} \le \Vert n_{tr}(0) \Vert _{L^2(\Omega )} + \frac{1}{\varepsilon } \int _0^t \big \Vert \widetilde{f}_3(s) \big \Vert _{L^2(\Omega )} \, ds \le I + \frac{T}{\varepsilon } (2 + 4 \beta I) \end{aligned}$$

and, hence,

$$\begin{aligned} \max _{0 \le t \le T} \Vert n_{tr}(t) \Vert _{L^2(\Omega )}, \, \max _{0 \le t \le T} \Vert n_{tr}'(t) \Vert _{L^2(\Omega )} \le 2I \end{aligned}$$

for $T>0$ sufficiently small.

*Step 3: Contraction property of* ${\mathcal {S}}$. We consider $(\widetilde{u}_1, \widetilde{v}_1, \widetilde{n}_{tr,1}, \widetilde{n}_{tr,1}'), (\widetilde{u}_2, \widetilde{v}_2, \widetilde{n}_{tr,2}, \widetilde{n}_{tr,2}') \in M_T$ and the corresponding solutions $(u_1, v_1, n_{tr,1}, n_{tr,1}') = {\mathcal {S}}(\widetilde{u}_1, \widetilde{v}_1, \widetilde{n}_{tr,1}, \widetilde{n}_{tr,1}') \in M_T$ and $(u_2, v_2, n_{tr,2}, n_{tr,2}') = {\mathcal {S}}(\widetilde{u}_2, \widetilde{v}_2, \widetilde{n}_{tr,2}, \widetilde{n}_{tr,2}') \in M_T$. We further introduce the notation

$$\begin{aligned} u := u_1 - u_2, \quad \widetilde{u} := \widetilde{u}_1 - \widetilde{u}_2 \end{aligned}$$

and similarly *v*, $n_{tr}$, $n_{tr}'$, $\widetilde{v}$, $\widetilde{n}_{tr}$ and $\widetilde{n}_{tr}'$. Then, we have to show that

$$\begin{aligned} \Vert (u, v, n_{tr}, n_{tr}') \Vert _{X_T} \le c \Vert (\widetilde{u}, \widetilde{v}, \widetilde{n}_{tr}, \widetilde{n}_{tr}') \Vert _{X_T} \end{aligned}$$

with a constant $c \in (0, 1)$ on a time interval [0, *T*] small enough. The norm in $X_T$ is defined as

$$\begin{aligned} \Vert (u, v, n_{tr}, n_{tr}') \Vert _{X_T}:= & {} \max _{0 \le t \le T} \Vert u(t) \Vert _{L^2(\Omega )} + \max _{0 \le t \le T} \Vert v(t) \Vert _{L^2(\Omega )} \\&+ \max _{0 \le t \le T} \Vert n_{tr}(t) \Vert _{L^2(\Omega )} + \max _{0 \le t \le T} \Vert n_{tr}'(t) \Vert _{L^2(\Omega )}. \end{aligned}$$

We obtain the following system by taking the difference of corresponding equations of the system for the 1- and the 2-variables, respectively:

80 $$\begin{aligned} {\left\{ \begin{array}{ll} \begin{aligned} \partial _t u - \Delta u &{}= A_n \big ( \widetilde{u}_1^+ - \widetilde{u}_2^+ \big ) + e^\frac{V_n}{2} \Big ( \widetilde{n}_{tr,1}^{[0,1]} - \widetilde{n}_{tr,2}^{[0,1]} \Big ) \\ &{}\quad - e^{V_n} \Big ( \widetilde{u}_1^+ \widetilde{n}_{tr,1}'^{[0,1]} - \widetilde{u}_2^+ \widetilde{n}_{tr,2}'^{[0,1]} \Big ) =: \widetilde{f}_1, \\ \partial _t v - \Delta v &{}= A_p \big ( \widetilde{v}_1^+ - \widetilde{v}_2^+ \big ) + e^\frac{V_p}{2} \Big ( \widetilde{n}_{tr,1}'^{[0,1]} - \widetilde{n}_{tr,2}'^{[0,1]} \Big ) \\ &{}\quad - e^{V_p} \Big ( \widetilde{v}_1^+ \widetilde{n}_{tr,1}^{[0,1]} - \widetilde{v}_2^+ \widetilde{n}_{tr,2}^{[0,1]} \Big ) =: \widetilde{f}_2, \\ \varepsilon \, \partial _t n_{tr} &{}= \widetilde{n}_{tr,1}'^{[0,1]} - \widetilde{n}_{tr,2}'^{[0,1]} - e^\frac{V_p}{2} \Big ( \widetilde{v}_1^+ \widetilde{n}_{tr,1}^{[0,1]} - \widetilde{v}_2^+ \widetilde{n}_{tr,2}^{[0,1]} \Big ) \\ &{}\quad - \widetilde{n}_{tr,1}^{[0,1]} + \widetilde{n}_{tr,2}^{[0,1]} + e^\frac{V_n}{2} \Big ( \widetilde{u}_1^+ \widetilde{n}_{tr,1}'^{[0,1]} - \widetilde{u}_2^+ \widetilde{n}_{tr,2}'^{[0,1]} \Big ) =: \widetilde{f}_3, \\ \varepsilon \, \partial _t n_{tr}' &{}= \widetilde{n}_{tr,1}^{[0,1]} - \widetilde{n}_{tr,2}^{[0,1]} - e^\frac{V_n}{2} \Big ( \widetilde{u}_1^+ \widetilde{n}_{tr,1}'^{[0,1]} - \widetilde{u}_2^+ \widetilde{n}_{tr,2}'^{[0,1]} \Big ) \\ &{}\quad - \widetilde{n}_{tr,1}'^{[0,1]} + \widetilde{n}_{tr,2}'^{[0,1]} + e^\frac{V_p}{2} \Big ( \widetilde{v}_1^+ \widetilde{n}_{tr,1}^{[0,1]} - \widetilde{v}_2^+ \widetilde{n}_{tr,2}^{[0,1]} \Big ) =: \widetilde{f}_4. \end{aligned} \end{array}\right. } \end{aligned}$$

We mention that *u* and *v* are subject to the boundary conditions

$$\begin{aligned} {\hat{n}} \cdot \Big (\nabla u + \frac{1}{2} u \nabla V_n \Big ) = {\hat{n}} \cdot \Big (\nabla v + \frac{1}{2} v \nabla V_p \Big ) = 0 \end{aligned}$$

and the homogeneous initial conditions

$$\begin{aligned} u(0) = v(0) = n_{tr}(0) = n_{tr}'(0) = 0. \end{aligned}$$

First, one finds

$$\begin{aligned} \max _{0 \le t \le T} \Vert u(t) \Vert _{L^2(\Omega )} \le C_1 \Vert u \Vert _{W_2(0, T)} \le C_1 C_2 \Vert \widetilde{f}_1 \Vert _{L^2((0, T) \times \Omega )} \end{aligned}$$

where $C_1 > 0$ is the constant resulting from the embedding $W_2(0, T) \hookrightarrow C([0, T], L^2(\Omega ))$. The constant $C_2 > 0$ originates from well-known parabolic regularity estimates for $\Vert u \Vert _{W_2(0, T)}$ in terms of the $L^2$-norms of $\widetilde{f}_1$ and $u(0)=0$. Therefore,

$$\begin{aligned} \max _{0 \le t \le T} \Vert u(t) \Vert _{L^2(\Omega )}&\le C_1 C_2 \Big ( \alpha \big \Vert \widetilde{u}_1^+ - \widetilde{u}_2^+ \big \Vert _{L^2((0, T) \times \Omega )} + \beta \big \Vert \widetilde{n}_{tr,1}^{[0,1]} - \widetilde{n}_{tr,2}^{[0,1]} \big \Vert _{L^2((0, T) \times \Omega )} \\&\quad + \beta \big \Vert \widetilde{u}_1^+ - \widetilde{u}_2^+ \big \Vert _{L^2((0, T) \times \Omega )} \big \Vert \widetilde{n}_{tr,1}'^{[0,1]} \big \Vert _{L^\infty ((0, T) \times \Omega )} \\&\quad + \beta \big \Vert \widetilde{u}_2^+ \big \Vert _{L^\infty ((0, T) \times \Omega )} \big \Vert \widetilde{n}_{tr,1}'^{[0,1]} - \widetilde{n}_{tr,2}'^{[0,1]} \big \Vert _{L^2((0, T) \times \Omega )} \Big ) \\&\le C_1 C_2 \left( \beta \Vert \widetilde{n}_{tr} \Vert _{L^2((0, T) \times \Omega )} + (\alpha + \beta ) \Vert \widetilde{u} \Vert _{L^2((0, T) \times \Omega )}\right. \\&\quad \left. +\, 2 \beta I \Vert \widetilde{n}_{tr}' \Vert _{L^2((0, T) \times \Omega )} \right) . \end{aligned}$$

Moreover, every $f \in C([0, T], L^2(\Omega ))$ fulfils

$$\begin{aligned} \Vert f \Vert _{L^2((0, T) \times \Omega )}^2 = \int _0^T \! \int _\Omega f^2 dx dt \le \int _0^T dt \max _{0 \le t \le T} \Vert f(t) \Vert _{L^2(\Omega )}^2 = T \Vert f \Vert _{C([0, T], L^2(\Omega ))}^2 \end{aligned}$$

and we proceed with the previous estimates to derive

$$\begin{aligned} \max _{0 \le t \le T} \Vert u(t) \Vert _{L^2(\Omega )}\le & {} C_1 C_2 (\alpha + 2 \beta I) \sqrt{T} \Bigg ( \Vert \widetilde{n}_{tr} \Vert _{C([0, T], L^2(\Omega ))} \\&+ \Vert \widetilde{u} \Vert _{C([0, T], L^2(\Omega ))} + \Vert \widetilde{n}_{tr}' \Vert _{C([0, T], L^2(\Omega ))} \Bigg ). \end{aligned}$$

In a similar way, we arrive at

$$\begin{aligned} \max _{0 \le t \le T} \Vert v(t) \Vert _{L^2(\Omega )}\le & {} C_1 C_2 (\alpha + 2 \beta I) \sqrt{T} \Bigg ( \Vert \widetilde{n}_{tr}' \Vert _{C([0, T], L^2(\Omega ))} \\&+ \Vert \widetilde{v} \Vert _{C([0, T], L^2(\Omega ))} + \Vert \widetilde{n}_{tr} \Vert _{C([0, T], L^2(\Omega ))} \Bigg ). \end{aligned}$$

Due to $n_{tr}(0) = 0$, one obtains

$$\begin{aligned} n_{tr}(t) = \frac{1}{\varepsilon } \int _0^t \widetilde{f}_3 \, ds \end{aligned}$$

for $t \in [0, T]$ and, using similar techniques as above,

$$\begin{aligned} \max _{0 \le t \le T} \Vert n_{tr}(t) \Vert _{L^2(\Omega )}&\le \frac{1}{\varepsilon } \int _0^T \Vert \widetilde{f}_3 \Vert _{L^2(\Omega )} \, ds \le \frac{\sqrt{T}}{\varepsilon } \Vert \widetilde{f}_3 \Vert _{L^2((0, T) \times \Omega )} \\&\le \frac{1 + 2 \beta I}{\varepsilon } \sqrt{T} \Bigg ( \Vert \widetilde{u} \Vert _{L^2((0, T) \times \Omega )} + \Vert \widetilde{v} \Vert _{L^2((0, T) \times \Omega )} \\&\quad + \Vert \widetilde{n}_{tr} \Vert _{L^2((0, T) \times \Omega )} + \Vert \widetilde{n}_{tr}' \Vert _{L^2((0, T) \times \Omega )} \Bigg ) \\&\le \frac{1 + 2 \beta I}{\varepsilon } T \Bigg ( \Vert \widetilde{u} \Vert _{C([0, T], L^2(\Omega ))} + \Vert \widetilde{v} \Vert _{C([0, T], L^2(\Omega ))} \\&\quad + \Vert \widetilde{n}_{tr} \Vert _{C([0, T], L^2(\Omega ))} + \Vert \widetilde{n}_{tr}' \Vert _{C([0, T], L^2(\Omega ))} \Bigg ). \end{aligned}$$

Note that because of $\widetilde{f}_4 = -\widetilde{f}_3$, the last estimate equally serves as an upper bound for $\Vert n_{tr}'(t) \Vert _{L^2(\Omega )}$. Taking the sum of the above estimates and choosing $T > 0$ sufficiently small yields

$$\begin{aligned} \Vert (u, v, n_{tr}, n_{tr}') \Vert _{X_T} \le c\, \Vert (\widetilde{u}, \widetilde{v}, \widetilde{n}_{tr}, \widetilde{n}_{tr}') \Vert _{X_T} \end{aligned}$$

with some $$c \in (0, 1)$$.

*Step 4: Solution of* (1). Step 2 and Step 3 imply that for $T > 0$ sufficiently small the mapping ${\mathcal {S}}: M_T \rightarrow M_T$ is a contraction. Banach’s fixed point theorem, thus, guarantees that there exists a unique $(u, v, n_{tr}, n_{tr}') \in M_T$ such that ${\mathcal {S}}(u, v, n_{tr}, n_{tr}') = (u, v, n_{tr}, n_{tr}')$. Moreover, due to standard parabolic regularity for (*u*, *v*), the fixed-point $(u, v, n_{tr}, n_{tr}')$ is the unique weak solution of

81 $$\begin{aligned} {\left\{ \begin{array}{ll} \begin{aligned} \partial _t u - \Delta u &{}= A_n u^+ + e^\frac{V_n}{2} n_{tr}^{[0,1]} - e^{V_n} u^+ n_{tr}'^{[0,1]}, \\ \partial _t v - \Delta v &{}= A_p v^+ + e^\frac{V_p}{2} n_{tr}'^{[0,1]} - e^{V_p} v^+ n_{tr}^{[0,1]}, \\ \varepsilon \, \partial _t n_{tr} &{}= n_{tr}'^{[0,1]} - e^\frac{V_p}{2} v^+ n_{tr}^{[0,1]} - n_{tr}^{[0,1]} + e^\frac{V_n}{2} u^+ n_{tr}'^{[0,1]}, \\ \varepsilon \, \partial _t n_{tr}' &{}= n_{tr}^{[0,1]} - e^\frac{V_n}{2} u^+ n_{tr}'^{[0,1]} - n_{tr}'^{[0,1]} + e^\frac{V_p}{2} v^+ n_{tr}^{[0,1]}. \end{aligned} \end{array}\right. } \end{aligned}$$

In order to prove the non-negativity of *u*, *v*, $n_{tr}$ and $n_{tr}'$, we adapt an argument from [26]. First, we define

$$\begin{aligned} h := \min (0, u) \end{aligned}$$

on $[0, T] \times \Omega$ and notice that $h \le 0$ and $h(t=0) = 0$ a.e. since $u(0) \ge 0$ a.e. We now multiply the first equation in (81) with *h* and integrate over $(0, t) \times \Omega$ for $t \in [0, T]$. This yields

82 $$\begin{aligned} \int _0^t \int _\Omega \partial _s u \, h \, dx\,ds= & {} \int _0^t \int _\Omega \Delta u \, h \, dx\,ds + \int _0^t \int _\Omega A_n u^+ h \, dx\,ds \nonumber \\&+ \int _0^t \int _\Omega \Big ( e^\frac{V_n}{2} n_{tr}^{[0,1]} - e^{V_n} u^+ n_{tr}'^{[0,1]} \Big ) \, h \, dx\,ds. \end{aligned}$$

The first term on the right hand side of (82) can be seen to be non-positive using integration by parts and the boundary condition from (75):

$$\begin{aligned} \int _0^t \int _\Omega \Delta u \, h \, dx\,ds&= - \int _0^t \int _\Omega \nabla u \cdot \nabla h \, dx\,ds - \frac{1}{2} \int _0^t \int _{\partial \Omega } u\, h\, {\hat{n}} \cdot \nabla V_n \, d\sigma \, ds \\&\le - \int _0^t \int _\Omega \nabla u \cdot \nabla h \, dx\,ds = - \int _0^t \int _\Omega \nabla h \cdot \nabla h \, dx\,ds \le 0 \end{aligned}$$

due to $u h \ge 0$, ${\hat{n}} \cdot \nabla V_n \ge 0$, and since $\nabla h \ne 0$ holds true only in the case $u < 0$, where we have $\nabla u = \nabla h$ in $L^2$, see e.g. [14]. Moreover,

$$\begin{aligned} \int _0^t \int _\Omega A_n u^+ h \, dx\,ds = 0, \end{aligned}$$

and the third term in (82) is again non-positive as an integral over non-positive quantities:

$$\begin{aligned} \int _0^t \int _\Omega \Big ( e^\frac{V_n}{2} n_{tr}^{[0,1]} - e^{V_n} u^+ n_{tr}'^{[0,1]} \Big ) \, h \, dx\,ds = \int _0^t \int _\Omega e^\frac{V_n}{2} n_{tr}^{[0,1]} \, h \, dx\,ds \le 0 \end{aligned}$$

as a consequence of $u^+ h = 0$ in $L^2(\Omega )$. The left hand side of (82) can be reformulated as

$$\begin{aligned} \int _0^t \int _\Omega \partial _s u \, h \, dx\,ds = \int _0^t \int _\Omega \partial _s h \, h \, dx\,ds = \frac{1}{2} \int _\Omega \int _0^t \Big ( \frac{d}{ds} \, h^2 \Big ) \, ds\,dx = \frac{1}{2} \Vert h(t) \Vert _{L^2(\Omega )}^2. \end{aligned}$$

For the first step, we have used that the integrand $\partial _s u \, h$ only contributes to the integral if $h < 0$. But in this case, $u = h$ and, hence, $\partial _s u = \partial _s h$ in $L^2$, see e.g. [14]. This proves $\Vert h(t) \Vert _{L^2(\Omega )} \le 0$ for all $t \in [0, T]$, which establishes $h(t) = 0$ in $L^2(\Omega )$ for all $t \in [0, T]$, and thus $u(t, x) \ge 0$ for all $t \in [0, T]$ and a.e. $x \in \Omega$. In the same way, one can show that $v(t, x) \ge 0$ for all $t \in [0, T]$ and a.e. $x \in \Omega$.

The non-negativity of $n_{tr}$ follows from a similar idea using

$$\begin{aligned} h := \min (0, n_{tr}). \end{aligned}$$

Again, $h \le 0$ and $h(t=0) = 0$ due to $n_{tr}(0) \ge 0$. Multiplying the third equation of (81) with *h* and integrating over $(0, t) \times \Omega$, $t \in [0, T]$, we find

$$\begin{aligned}&\varepsilon \int _0^t \int _\Omega \partial _s n_{tr} \, h \, dx\,ds = \int _0^t \int _\Omega \Big ( n_{tr}'^{[0,1]} - e^\frac{V_p}{2} v^+ n_{tr}^{[0,1]} - n_{tr}^{[0,1]} + e^\frac{V_n}{2} u^+ n_{tr}'^{[0,1]} \Big ) h \, dx \, ds. \end{aligned}$$

As before, all terms under the integral on the right hand side involving $n_{tr}^{[0, 1]}$ vanish. Consequently,

$$\begin{aligned} \frac{\varepsilon }{2} \Vert h(t) \Vert _{L^2(\Omega )}^2 = \varepsilon \int _0^t \int _\Omega \partial _s h \, h \, dx\,ds = \int _0^t \int _\Omega \Big ( n_{tr}'^{[0,1]} + e^\frac{V_n}{2} u^+ n_{tr}'^{[0,1]} \Big ) h \, dx \, ds \le 0 \end{aligned}$$

for all $t \in [0, T]$. The same result holds true for $n_{tr}'$. Therefore, we have verified that $n_{tr}(t, x)$, $n_{tr}'(t, x) \ge 0$ for all $t \in [0, T]$ and a.e. $x \in \Omega$.

The non-negativity of $n_{tr}$ and $n_{tr}'$ together with $n_{tr}' = 1 - n_{tr}$ from (73) now even imply

$$\begin{aligned} n_{tr}(t, x), \, n_{tr}'(t, x) \in [0, 1], \quad \text {for all}\quad t \in [0, T] \quad \text {and a.e.}\quad x \in \Omega . \end{aligned}$$

This allows us to identify the unique weak solution $(u, v, n_{tr}, n_{tr}')$ of (81) to equally solve

83 $$\begin{aligned} {\left\{ \begin{array}{ll} \begin{aligned} \partial _t u - \Delta u &{}= A_n u + e^\frac{V_n}{2} n_{tr} - e^{V_n} u (1-n_{tr}), \\ \partial _t v - \Delta v &{}= A_p v + e^\frac{V_p}{2} (1-n_{tr}) - e^{V_p} v\, n_{tr}, \\ \varepsilon \, \partial _t n_{tr} &{}= 1-n_{tr} - e^\frac{V_p}{2} v\, n_{tr} - n_{tr} + e^\frac{V_n}{2} u (1-n_{tr}), \end{aligned} \end{array}\right. } \end{aligned}$$

which is the transform version of the original problem (1).

Up to now, we have proven that there exists a unique solution $(u, v, n_{tr}) \in C([0, T], L^2(\Omega ))^3$ such that $(u, v, n_{tr}, 1 - n_{tr}) \in M_T$ on a sufficiently small time interval [0, *T*].

*Step 5: Global solution.* We now fix $$T^*>0$$ in such a way that $$[0, T^*)$$ is the maximal time interval of existence for the solution $$(u, v, n_{tr}) \in C([0, T], L^2(\Omega ))^3$$ of (83). Moreover, we choose some arbitrary $$q \in {\mathbb {N}}_{\ge 2}$$ and multiply the first equation in (83) with $$u^{q-1}$$. Integrating over $$\Omega$$ at time $$t \in [0, T^*)$$ gives

$$\begin{aligned} \frac{d}{dt} \int _{\Omega } \frac{u^q}{q} \, dx= & {} \int _{\Omega } u^{q-1} \partial _t u \, dx = \int _{\Omega } u^{q-1} \Delta u \, dx + \int _{\Omega } A_n u^{q} \, dx \\&+ \int _{\Omega } u^{q-1} \Big ( e^\frac{V_n}{2} n_{tr} - e^{V_n} u (1-n_{tr}) \Big ) \, dx. \end{aligned}$$

Integration by parts and the estimates $|A_n| \le \alpha$, $\big |e^\frac{V_n}{2} n_{tr} - e^{V_n} u (1-n_{tr})\big | \le \beta (1 + u)$ further yield

$$\begin{aligned} \frac{d}{dt} \int _{\Omega } \frac{u^q}{q} \, dx\le & {} - (q-1) \int _\Omega u^{q-2} | \nabla u |^2 \, dx - \frac{1}{2} \int _{\partial \Omega } u^q \, {\hat{n}} \cdot \nabla V_n \, d\sigma \, ds \\&+ \alpha \int _{\Omega } u^{q} \, dx + \beta \int _\Omega (u^{q-1} + u^q) \, dx. \end{aligned}$$

Moreover, we derive

$$\begin{aligned} \int _\Omega u^{q-1} \, dx = \int _{\{ u \le 1 \}} u^{q-1} dx + \int _{\{ u > 1 \}} \frac{u^q}{u} dx \le \int _\Omega 1 dx + \int _\Omega u^q \, dx = 1 + \int _\Omega u^q \, dx \end{aligned}$$

where we used $|\Omega | = 1$. Hence,

84 $$\begin{aligned} \frac{d}{dt} \int _{\Omega } \frac{u^q}{q} \, dx \le \beta + (\alpha + 2 \beta ) \int _\Omega u^q \, dx \le \gamma \left( 1 + \int _\Omega u^q \, dx \right) \end{aligned}$$

after defining $\gamma := \alpha + 2 \beta$. This results in

$$\begin{aligned} \frac{d}{dt} \int _{\Omega } u^q \, dx \le \gamma q \left( 1 + \int _\Omega u^q \, dx \right) , \end{aligned}$$

which can be integrated over time from 0 to *t*:

$$\begin{aligned} \Vert u(t) \Vert _{L^q(\Omega )}^q \le \Vert u(0) \Vert _{L^q(\Omega )}^q + \gamma q \int _0^t \Big ( 1 + \Vert u(s) \Vert _{L^q(\Omega )}^q \Big ) \, ds. \end{aligned}$$

From this generalised Gronwall-type inequality, we deduce (cf. [2])

$$\begin{aligned} \Vert u(t) \Vert _{L^q(\Omega )}^q \le \Vert u(0) \Vert _{L^q(\Omega )}^q e^{\gamma qt} + e^{\gamma qt} - 1 < \left( 1 + \Vert u(0) \Vert _{L^q(\Omega )}^q \right) e^{\gamma qt} \end{aligned}$$

and

$$\begin{aligned} \Vert u(t) \Vert _{L^q(\Omega )} \le \big (1 + \Vert u(0) \Vert _{L^q(\Omega )} \big ) e^{\gamma t} \le I e^{\gamma t} \end{aligned}$$

since $1+\Vert u(0) \Vert _{L^q(\Omega )} \le 1+\Vert u(0) \Vert _{L^\infty (\Omega )} \le I$. As $I e^{\gamma t}$ is independent of *q*, we even arrive at

$$\begin{aligned} \Vert u(t) \Vert _{L^\infty (\Omega )} \le I e^{\gamma t}. \end{aligned}$$

In the same way, we can show that $\Vert v(t) \Vert _{L^\infty (\Omega )} \le I e^{\gamma t}$ for all $t \in [0, T^*)$. As a consequence, we obtain that the solution $(u, v, n_{tr}) \in C([0, T], L^2(\Omega ))^3$ can be extended for all times, i.e. $T^*= \infty$.

*Step 6:* $L^\infty$-*bounds for* *n* *and* *p*. We now prove the linearly growing $L^{\infty }$-bounds (11) for *n* and *p*. We only detail the bound for *p* and sketch how the bound for *n* follows in a similar fashion. After recalling (with $\tau _p=1$ w.l.o.g.)

$$\begin{aligned} \partial _t p = \nabla \cdot J_p + \left( 1 - n_{tr} - \frac{p}{p_0 e^{-V_p}} n_{tr} \right) ,\quad J_p=e^{-V_p} \nabla \left( p\, e^{V_p} \right) , \end{aligned}$$

we introduce the variable $w=p \,e^{V_p}$ and observe that $\nabla \cdot J_p = \nabla \cdot \left( e^{-V_p} \nabla w \right) =e^{-V_p}\left( \Delta w - \nabla V_p \cdot \nabla w\right)$ and thus,

85 $$\begin{aligned} \partial _t w = \Delta w - \nabla V_p \cdot \nabla w + e^{V_p}\left( 1 - n_{tr} - \frac{n_{tr}}{p_0} w \right) , \end{aligned}$$

while the no-flux boundary condition ${\hat{n}} \cdot J_p = 0$ on $\partial \Omega$ transforms to the homogeneous Neumann condition ${\hat{n}} \cdot \nabla w = 0$ on $\partial \Omega$.

Next, by testing (85) with the positive part $(w-r-st)_{+}:=\max \{0, w-r-st\}$ for two constant $r,s>0$ to be chosen, we calculate by integration by parts in the first two terms

$$\begin{aligned}&\frac{d}{dt} \frac{1}{2} \int _{\Omega } (w-r-st)_{+}^2\,dx \\&\quad = \int _{\Omega } (w-r-st)_{+} \left( \Delta w - \nabla V_p \cdot \nabla w + e^{V_p}\Bigl ( 1 - n_{tr} - \frac{n_{tr}}{p_0} w \Bigr ) -s \right) dx \\&\quad =- \int _{\Omega } \mathbb {1}_{w\ge r+st} |\nabla w|^2 \,dx - \int _{\Omega } \nabla V_p \cdot \nabla \frac{(w-r-st)_{+}^2}{2}\,dx\\&\qquad + \int _{\Omega } (w-r-st)_{+} \left( e^{V_p}\Bigl ( 1 - n_{tr} - \frac{n_{tr}}{p_0} w \Bigr ) -s\right) dx\\&\quad \le \frac{\Vert \Delta V_p\Vert _{L^\infty (\Omega )}}{2} \int _{\Omega } (w-r-st)_{+}^2\,dx \\&\qquad + \int _{\Omega } (w-r-st)_{+} \left( e^{V_p}\Bigl ( 1 - n_{tr} - \frac{n_{tr}}{p_0} w \Bigr ) -s\right) dx, \end{aligned}$$

since ${\hat{n}} \cdot V_p\ge 0$ by assumption (4). Moreover, since $n_{tr}\in [0,1]$ and $w\ge 0$, we have

$$\begin{aligned} \frac{d}{dt} \frac{1}{2} \int _{\Omega } (w-r-st)_{+}^2\,dx\le & {} \frac{\Vert \Delta V_p\Vert _{L^\infty (\Omega )}}{2} \int _{\Omega } (w-r-st)_{+}^2\,dx \\&+ \int _{\Omega } (w-r-st)_{+} \Bigl (\Vert e^{V_p}\Vert _{L^\infty (\Omega )} -s\Bigr )dx. \end{aligned}$$

Thus, by choosing $s {:}{=}\Vert e^{V_p}\Vert _{L^\infty (\Omega )}$ and $r {:}{=}\Vert w(\tau ,\cdot )\Vert _{L^\infty (\Omega )}$ for some time $\tau \ge 0$, we conclude that

$$\begin{aligned} \frac{d}{dt} \int _{\Omega } (w-r-st)_{+}^2\,dx \le \Vert \Delta V_p\Vert _{L^\infty (\Omega )} \int _{\Omega } (w-r-st)_{+}^2\,dx, \end{aligned}$$

and a Gronwall lemma implies

86 $$\begin{aligned} \Vert w(t,\cdot )\Vert _{L^\infty (\Omega )} \le \Vert w(\tau ,\cdot )\Vert _{L^\infty (\Omega )}+\Vert e^{V_p}\Vert _{L^\infty (\Omega )}\,t,\quad \text {for all } t\ge \tau \ge 0. \end{aligned}$$

Transforming back, this yields

87 $$\begin{aligned} \Vert p(t,\cdot )\Vert _{L^\infty (\Omega )}\le & {} \frac{1}{\inf \{e^{V_p}\}}\left( \Vert p(\tau ,\cdot )\Vert _{L^\infty (\Omega )}\Vert e^{V_p}\Vert _{L^\infty (\Omega )}+\Vert e^{V_p}\Vert _{L^\infty (\Omega )}\,t\right) ,\nonumber \\&\text {for all } t\ge \tau \ge 0. \end{aligned}$$

In order to deduce the analogue bound for *n* in (11), we consider (with $\tau _n=1$ w.l.o.g.)

$$\begin{aligned} \partial _t n = \nabla \cdot J_n + \left( n_{tr} - \frac{n}{n_0 e^{-V_n}} \bigl (1-n_{tr}\bigr ) \right) ,\quad J_n=e^{-V_n} \nabla \big (n\, e^{V_n} \bigr ). \end{aligned}$$

We introduce the variable $\omega =n \,e^{V_n}$ and obtain in the same way as in (54)

$$\begin{aligned} \partial _t \omega = \Delta \omega - \nabla V_n \cdot \nabla \omega + e^{V_n}\left( n_{tr} - \frac{1-n_{tr}}{n_0} \omega \right) . \end{aligned}$$

Following the same arguments as above,

88 $$\begin{aligned} \Vert \omega (t,\cdot )\Vert _{L^\infty (\Omega )} \le \Vert \omega (\tau ,\cdot )\Vert _{L^\infty (\Omega )}+\Vert e^{V_n}\Vert _{L^\infty (\Omega )}\,t,\quad \text {for all } t\ge \tau \ge 0. \end{aligned}$$

Transforming back, this yields

89 $$\begin{aligned} \Vert n(t,\cdot )\Vert _{L^\infty (\Omega )} \le \frac{1}{\inf \{e^{V_n}\}}\left( \Vert n(\tau ,\cdot )\Vert _{L^\infty (\Omega )}\Vert e^{V_n}\Vert _{L^\infty (\Omega )}+\Vert e^{V_n}\Vert _{L^\infty (\Omega )}\,t\right) ,\,\, \text {for all } t\ge \tau \ge 0 \end{aligned}$$

and thus (11).

*Step 7: Regularity and bounds for* $n_{tr}$. We still have to verify $n_{tr} \in C([0, T], L^\infty (\Omega ))$ for all $T > 0$. Now, let $T>0$ and recall that

$$\begin{aligned} n_{tr}(t) = n_{tr}(0) + \frac{1}{\varepsilon } \int _0^t \left( 1-n_{tr} - e^{V_p} p \, n_{tr} - n_{tr} + e^{V_n} n (1-n_{tr}) \right) \, ds \end{aligned}$$

in $L^2(\Omega )$ for all $t \in [0,T]$. Considering a sequence $(t_k)_{k \in {\mathbb {N}}} \subset [0, T]$, $t_k \rightarrow t$, we thus arrive at

$$\begin{aligned} \Vert n_{tr}(t_k) - n_{tr}(t) \Vert _{L^\infty (\Omega )}\le & {} \frac{1}{\epsilon } \left| \int _t^{t_k}\! \Vert 1-n_{tr} - e^{V_p} p \, n_{tr} - n_{tr}\right. \\&\left. + e^{V_n} n (1-n_{tr}) \Vert _{L^\infty (\Omega )} \, ds \right| \le \frac{|t_k\! -\! t|}{\epsilon } C_{T} \rightarrow 0 \end{aligned}$$

for $k \rightarrow \infty$. This proves the assertion.

The claim $\partial _t n_{tr} \in C([0, T], L^2(\Omega ))$ for all $T > 0$ is an immediate consequence of the last equation in (83) together with the $L^2$-continuity and $L^\infty$-bounds of *u*, *v* and $n_{tr}$.

Next, concerning the bounds (12), we recall system (1) and observe that for all $\varepsilon \in (0,\varepsilon _0]$

$$\begin{aligned} \varepsilon \partial _t n_{tr}&= h(n_{tr}):=R_p(p, n_{tr}) - R_n(n, n_{tr}), \end{aligned}$$

in the sense of $L^2(\Omega )$, where $h(n_{tr}=0)\ge \frac{1}{\tau _p}>0$ and $h(n_{tr}=1)\le -\frac{1}{\tau _n} < 0$ uniformly for all non-negative *n* and *p*. Therefore, wherever $n_{tr,I}(x)=0$ (or analogously $n_{tr,I}(x)=1$), an elementary argument proves that $n_{tr}(t,x)$ grows (or decreases) linearly in time and decays back to 0 (or 1) at most like $(a+bt)^{-1}$. More precisely, we reuse the transformed variable $w = p \, e^{V_p}$ and find

$$\begin{aligned} \varepsilon \partial _t n_{tr} \ge \frac{1}{\tau _p}\left[ 1 - \left( 1+\frac{\tau _p}{\tau _n} + \frac{\Vert w\Vert _{L^\infty (\Omega )}}{ p_0}\right) n_{tr}\right] \ge \frac{1}{\tau _p}\left[ 1 - \left( 1+\frac{\tau _p}{\tau _n} + \frac{r+st}{ p_0}\right) n_{tr}\right] \end{aligned}$$

for some constants *r* and *s* due to the estimate (86). Setting $\tau _n {:}{=}\tau _p {:}{=}1$ w.l.o.g., we have

$$\begin{aligned} \varepsilon \partial _t n_{tr} \ge 1 - (\widetilde{r} + \widetilde{s} t) n_{tr}. \end{aligned}$$

with appropriate $\widetilde{r}, \widetilde{s} > 0$ independent of $\varepsilon$. By observing that the ODE $\varepsilon _0{\dot{y}} = 1 - (\widetilde{r} + \widetilde{s} t) y$ features the positive nullcline $y_0(t) = 1/(\widetilde{r} + \widetilde{s} t)$, which moreover attracts all solution trajectories, standard comparison arguments (pointwise in $x\in \Omega$) imply that for all times $\tau > 0$, there exist positive constants $\eta = \eta (\varepsilon _0, \tau , \tau _n, \tau _p)$, $\theta = \theta (C_n,C_p,K_n,K_p)$ and a sufficiently small constant $\gamma (\tau ,C_n,C_p,K_n,K_p)>0$ such that

$$\begin{aligned} n_{tr}(t,x) \ge \min \Bigl \{\eta t, \frac{\gamma }{1+\theta t}\Bigr \} \quad \text {for all } t\ge 0 \text { and a.e. } x\in \Omega \end{aligned}$$

where $\eta \tau = \frac{\gamma }{1+\theta \tau }$ such that the linear and the inverse linear bound intersect at time $\tau$.

Finally, the upper bounds (12) follow from analogue arguments.

*Step 8: Lower bounds for* *n* *and* *p*. Finally, we prove the bounds (13). We will only detail the argument for the lower bound on *n*, as the bound for *p* follows in an analogue way. Recalling the transformed equation for $\omega =e^{V_n}n$ (satisfying ${\hat{n}} \cdot \nabla \omega = 0$ on $\partial \Omega$), we estimate

90 $$\begin{aligned} \partial _t \omega = \Delta \omega - \nabla V_n \cdot \nabla \omega + e^{V_n}\left( n_{tr} - \frac{1-n_{tr}}{n_0} \omega \right) \ge \Delta \omega -\nabla V_n \cdot \nabla \omega - \alpha \, \omega + c n_{tr}, \end{aligned}$$

where $\alpha > 0$ and $c>0$ are positive constants due to the assumptions (4) and $e^{V_n} \omega n_{tr}\ge 0$.

Next, we use (12), i.e. that for all $\tau >0$ fixed, there exist constants $\eta$, $\theta$ and $\gamma$ such that $n_{tr}(t,x) \ge \eta t$ for all $0\le t \le \tau$ and a.e. $x\in \Omega$, while $n_{tr}(t,x) \ge \gamma /(1 + \theta t)$ for all $t \ge \tau$ and a.e. $x\in \Omega$. Then, by introducing the negative part $(\omega )_{-}:= \min \{\omega ,0\}$ and testing (90) with $\bigl (\omega -\frac{\mu t^2}{2}\bigr )_{-}$ for a constant $\mu >0$ to be chosen below, we estimate

$$\begin{aligned}&\frac{d}{dt} \frac{1}{2} \int _{\Omega } \Bigl (\omega -\frac{\mu t^2}{2}\Bigr )_{-}^2 \,dx = \int _{\Omega } \Bigl (\omega -\frac{\mu t^2}{2}\Bigr )_{-}\left( \partial _t \omega -\mu t\right) dx\\&\quad = \int _{\Omega } \left| \Bigl (\omega -\frac{\mu t^2}{2}\Bigr )_{-}\right| \left( -\partial _t \omega +\mu t\right) dx\\&\quad \le \int _{\Omega } \left| \Bigl (\omega -\frac{\mu t^2}{2}\Bigr )_{-}\right| \left( - \Delta \omega + \nabla V_n \cdot \nabla \omega + \alpha \omega - c n_{tr} +\mu t\right) dx\\&\quad = \int _{\Omega } \Bigl (\omega -\frac{\mu t^2}{2}\Bigr )_{-} \left( \Delta \omega - \nabla V_n \cdot \nabla \omega \right) dx + \int _{\Omega } \left| \Bigl (\omega -\frac{\mu t^2}{2}\Bigr )_{-}\right| \left( \alpha \omega - c n_{tr} +\mu t\right) dx\\&\quad \le - \int _{\Omega } \mathbb {1}_{\omega \le \frac{\mu t^2}{2}} |\nabla \omega |^2\,dx - \frac{1}{2} \int _\Omega \nabla \Bigl (\omega -\frac{\mu t^2}{2}\Bigr )_{-}^2 \cdot \nabla V_n \, dx \\&\qquad + \int _{\Omega } \left| \Bigl (\omega -\frac{\mu t^2}{2}\Bigr )_{-}\right| \left( \alpha \omega -c n_{tr}+ \mu t\right) dx. \end{aligned}$$

Thus, for $0\le t \le \tau$ when $n_{tr}(t,x) \ge \eta t$, we have

$$\begin{aligned} \frac{d}{dt} \frac{1}{2} \int _{\Omega } \left( \omega -\frac{\mu t^2}{2}\right) _{-}^2 \,dx&\le \int _\Omega \left( \omega -\frac{\mu t^2}{2}\right) _{-}^2 \frac{\Delta V_n}{2} \, dx \\&\quad + \int _{\Omega } \left| \left( \omega -\frac{\mu t^2}{2}\right) _{-}\right| \left( \alpha \frac{\mu t^2}{2} -c \eta t + \mu t\right) dx. \end{aligned}$$

If we choose $\mu \bigl (\alpha \frac{\tau }{2}+1\bigr )\le c \eta$, we obtain

$$\begin{aligned} \frac{d}{dt} \int _{\Omega } \left( \omega -\frac{\mu t^2}{2}\right) _{-}^2 \,dx \le \Vert \Delta V_n\Vert _{L^\infty (\Omega )} \int _\Omega \left( \omega -\frac{\mu t^2}{2}\right) _{-}^2 \, dx. \end{aligned}$$

Hence, since $\int _{\Omega } \bigl (\omega (0,x)\bigr )_{-}^2dx=0$, we deduce from a Gronwall lemma

$$\begin{aligned} \int _{\Omega } \Bigl (\omega -\frac{\mu t^2}{2}\Bigr )_{-}^2 \,dx =0, \quad \text {for all } 0\le t \le \tau , \end{aligned}$$

which yields in particular $\omega (t,x)\ge \frac{\mu t^2}{2}$ for all $0 \le t \le \tau$ and a.e. $x\in \Omega$.

Moreover, for $t\ge \tau$ when $n_{tr}(t,x) \ge \frac{\gamma }{1+\theta t}$, we test (90) with $\bigl (\omega -\frac{\Gamma }{1+\theta t}\bigr )_{-}$ for a constant $\Gamma >0$ to be chosen below, and estimate similar to above

$$\begin{aligned} \frac{d}{dt} \frac{1}{2}&\int _{\Omega } \Bigl (\omega -\frac{\Gamma }{1+\theta t}\Bigr )_{-}^2 \,dx = \int _{\Omega } \Bigl (\omega -\frac{\Gamma }{1+\theta t}\Bigr )_{-} \left( \partial _t \omega +\frac{\Gamma \theta }{(1+\theta t)^2}\right) dx\\&\le \int _{\Omega } \Bigl (\omega -\frac{\Gamma }{1+\theta t}\Bigr )_{-} \left( \Delta \omega - \nabla V_n \cdot \nabla \omega - \alpha \omega + c n_{tr} + \frac{\Gamma \theta }{(1+\theta t)^2}\right) dx\\&\le - \int _{\Omega } \mathbb {1}_{\omega \le \frac{\Gamma }{1+\theta t}} |\nabla \omega |^2\,dx - \frac{1}{2} \int _\Omega \nabla \Bigl (\omega -\frac{\Gamma }{1+\theta t}\Bigr )_{-}^2 \cdot \nabla V_n \, dx \\&\quad + \int _{\Omega } \left| \Bigl (\omega -\frac{\Gamma }{1+\theta t}\Bigr )_{-}\right| \left( \alpha \omega - c n_{tr} - \frac{\Gamma \theta }{(1+\theta t)^2}\right) dx. \end{aligned}$$

And as $n_{tr}(t,x) \ge \frac{\gamma }{1+\theta t}$ for $t \ge \tau$, we find

$$\begin{aligned}&\frac{d}{dt} \frac{1}{2} \int _{\Omega } \Bigl (\omega -\frac{\Gamma }{1+\theta t}\Bigr )_{-}^2 \,dx \le \int _{\Omega } \Bigl (\omega -\frac{\Gamma }{1+\theta t}\Bigr )_{-}^2 \frac{\Delta V_n}{2} \, dx \\&\quad + \int _{\Omega } \left| \Bigl (\omega -\frac{\Gamma }{1+\theta t}\Bigr )_{-}\right| \left( \alpha \frac{\Gamma }{1+\theta t} - c \frac{\gamma }{1+\theta t}-\frac{\Gamma \theta }{(1+\theta t)^2}\right) dx. \end{aligned}$$

Choosing $\alpha \Gamma \le c \gamma$, we arrive at

$$\begin{aligned} \frac{d}{dt} \int _{\Omega } \Bigl (\omega -\frac{\Gamma }{1+\theta t}\Bigr )_{-}^2 \,dx \le \Vert \Delta V_n\Vert _{L^\infty (\Omega )} \int _{\Omega } \Bigl (\omega -\frac{\Gamma }{1+\theta t}\Bigr )_{-}^2 \, dx. \end{aligned}$$

By further reducing either $\Gamma$ or $\mu$, we are able to satisfy $\frac{\Gamma }{1+\theta \tau } = \frac{\mu \tau ^2}{2}$. On the one hand, this implies that $\int _{\Omega } \Bigl (\omega (\tau ,x) - \frac{\Gamma }{1+\theta \tau }\Bigr )_{-}^2 \,dx=0$, which results—by using a Gronwall argument—in

$$\begin{aligned} \int _{\Omega } \left( \omega (t,x)-\frac{\Gamma }{1+\theta t}\right) _{-}^2 \,dx=0, \quad \text {for all } t \ge \tau , \end{aligned}$$

and, hence, $\omega (t,x)\ge \frac{\Gamma }{1+\theta t}$ for all $t \ge \tau$ and a.e. $x\in \Omega$. On the other hand, the increasing and decreasing bounds now again intersect at time $\tau$ as desired. $\square$
